# Partner preferences for resources adapt to income and gender economic inequality

**DOI:** 10.1073/pnas.2527295123

**Published:** 2026-03-16

**Authors:** Macken Murphy, Sylvia K. Harmon-Jones, Auguste G. Harrington, Robert C. Brooks, Khandis R. Blake

**Affiliations:** ^a^Melbourne School of Psychological Sciences, University of Melbourne, Melbourne VIC 3010, Australia; ^b^School of Psychology, University of Wollongong, Wollongong, NSW 2522, Australia; ^c^Program in Psychology, New York University Abu Dhabi, Abu Dhabi P.O. Box 129188, United Arab Emirates; ^d^School of Biological, Earth and Environmental Sciences, University of New South Wales, Sydney NSW 2052, Australia

**Keywords:** mate preferences, gender inequality, human behavioral ecology

## Abstract

For over three decades, scholars have contested whether sex differences in ideal partner preferences for age, resources, and physical attractiveness result chiefly from evolution or cultural gender inequality. Here, we test a perspective compatible with each of these competing views: partner preferences for resources adapt strategically, such that they become stronger for the sex that is poorer and individuals who are poorer, as such preferences are more useful to these individuals. Our preregistered experiment placed 602 people from five countries in one of 45 virtual ecologies, varying participants’ personal income and their local gender economic inequality. Results show that people exhibit stronger ideal partner preferences for resources when they are poorer and when their sex is poorer. This pattern was evident in three different measures of stated partner preferences for resources, namely, ranked preferences and rated preferences for resource-relevant traits (e.g., whether a potential partner has a good job), and importance placed on dating or marrying up, financially. Preferences regarding age gaps were, surprisingly, unaffected, even though older age is associated with access to resources in naturally varying populations. Consistent with our theoretical perspective, which expects ideal partner preferences will adapt primarily to environmental variables that alter the fitness of those preferences, sex differences in stated preferences for physical attractiveness in romantic partners were largely unaffected. These results are discussed in the context of behavioral ecology, which offers a framework to interpret both our results and the mixed findings of past literature.

## Significance

Women, more than men, tend to say they prefer romantic partners with traits linked to access to resources. For 36 y, social scientists have debated whether this sex difference results from gender inequality or human evolution. Here, we experimentally manipulate gender economic inequality and income, finding that this sex difference reduces or disappears when women hold more resources while sex differences in ideal partner preferences for age and beauty remain unaffected. Further, we find that richer individuals relax their ideal partner preferences for resources. Our results suggest that sex differences in ideal partner preferences for resources are environmentally contingent, implying that traditional heterosexual preferences for hypergyny may erode with women’s economic advancement, despite public concerns to the contrary.

Some sex differences in heterosexual ideal partner preferences are almost cross-culturally universal (1). Multiple large, multinational studies have found that when selecting a long-term partner, women, relative to men, generally report a stronger desire for traits that signal access to material resources, whereas men, relative to women, are typically more concerned with physical attractiveness (1–4), but see refs. 5–7. Similarly, researchers have consistently found that women tend to desire men who are slightly older than themselves, whereas men desire women increasingly younger than themselves as they get older [(8, 9), but see ref. 10].^*^ For as long as these differences have been known, scholars have vigorously contested why these differences exist and the degree to which they are flexible, in a 36-y interdisciplinary debate that has attracted contributions from anthropologists (e.g., ref. 11), economists (e.g., ref. 12), biologists (e.g., ref. 13), and psychologists from multiple subfields (e.g., refs. 14 and 15).

One reason this debate has attracted sustained and diverse scholarly attention over this time period may be its escalating cultural stakes. In the mid-20th century, men had substantially more educational and career opportunities than women, and so, sex-differentiated ideal partner preferences for resources were easily satisfied, with hypergyny as a prevailing norm (16, 17). However, since then, socioeconomic and social gender differences have eroded substantially in many nations, with women obtaining more control of resources than their mothers and grandmothers (16, 17). In this context, the nature of these preferences has implications for the present and future of romantic relationships. Specifically, there is now a common concern—widely discussed on social media^†^ and in major news outlets (e.g., refs. 18–20)—that if women are inflexible in their desire to marry men at least as successful as themselves, then the more successful women become relative to men, the less likely women are to be content with their available potential partners. And given its assumptions, this concern is logically sound, as modeling work by Brooks et al. (21) has shown that if preferences for hypergyny were inflexible, then increased control of resources by women would lead to a steep reduction in pairing opportunities for rich women and poor men. So, whether or not hypergyny erodes with rising gender economic inequality implicates not only theory but the likely prevalence of romantic relationships in nations where women’s socioeconomic power advances.

While in this paper, we test a behavioral ecological perspective (to be discussed further), the debate over the influence of gender equality on romantic partner preferences has historically centered on two distinct theoretical perspectives: evolutionary psychology and biosocial role theory. Evolutionary psychologists generally argue that these sex differences reflect evolved psychological differences between men and women (1, 8). Accounts vary, but one perspective is that men’s investment in offspring is proportionately more material (e.g., food provisioning), whereas women’s is proportionately more physical (i.e., pregnancy, lactation), and so women evolved stronger attraction to cues to material resources (including older age, since older men tend to have more resources), whereas men evolved stronger attraction to physical cues, specifically, youth and beauty (1, 8). Youth is hypothesized to be more desirable for men than women because women’s fertility declines more steeply with age than men’s does (9). Beauty is commonly hypothesized to be more desirable for men than women because access to fertile mates is suggested to be the primary limit on men’s reproductive success (15), and cues to women’s beauty are traditionally hypothesized to be visual cues to fertility—though this connection is tenuous (22).^‡^ The scholars who make this adaptationist argument lean on the cross-cultural prevalence of sex differences in these mate preferences to make their case (1, 23). Rhetorically, to paraphrase Buss [(1), p. 40]: if these sex differences result from culture, why do they appear in almost all cultures?

Biosocial role theorists have argued, to the contrary, that these sex differences instead result from women’s culturally assigned place in society (14, 24). They explain the observed near-universality of certain sex-differentiated partner preferences with reference to evolved physical (and possibly temperamental) sex differences, which they say historically made men and women more likely to be assigned different social roles: men as resource providers and women as homemakers (14, 24). These roles incentivize women to feel more attracted to “provider” traits (ambition, good financial prospects, older age) and men to be more attracted to traits that fit a more socially submissive “homemaker” role (younger age). Explanations provided for the observed sex differentiation in ideal partner preferences for beauty are varied, including that beauty may be more useful for a homemaker as it helps with social interactions, or that perhaps people favor beauty for its “perceived association” with sexual pleasure and a handsome mate is less openly valued by women due to cultural stigmatization of women’s sexuality [(24), p. 419].

From biosocial role theory, it naturally follows that men and women in cultures with higher gender inequality should exhibit larger sex differences in partner preferences, as they have larger differences in social roles (24). As a result, much of this debate centers on whether sex differences in partner preferences positively covary with national gender inequality, an indicator of divergent gender roles (1, 3, 4, 24, 28, 29). When gender inequality has predicted sex differences in partner preferences, it has been cited as evidence for biosocial role theory (e.g., refs. 2 and 24). Null or inverse correlations between these variables have usually been interpreted as ratifications of evolutionary psychological science (e.g., refs. 3 and 27). In the latter case, this is validation only by process of elimination—a positive effect of gender inequality is also compatible with evolutionary psychological theory, it is just not generally predicted (30, 31).

Despite plentiful cross-cultural research, methodological inconsistencies make it challenging to draw a clear interpretation of the empirical support for each theory. Different research teams have used different indices of gender inequality (for instance the GGGI, the GGI, the GEM, the GII, the GRDI, and GDI; composite metrics that integrate multiple facets of gender inequality, economic and social), and then made radically different variable construction and analysis choices, often resulting in different teams reaching opposite conclusions from the same data (e.g., refs. 2, 24, and 26). The debate has also seen each side insinuate political motivations in the other (e.g., refs. 32, pp. 309–310; 33), damaging confidence that these investigations have been conducted impartially.

Despite these reasons for caution, there are common conclusions. As predicted by biosocial role theory, age gaps in heterosexual married couples consistently reduce with rising gender equality (2, 4, 24, 26). Also consistent with biosocial role theory, most studies have found some positive association between sex differences in stated partner preferences for resources and gender inequality (2–4, 24, 25). However, Zhang et al. (3) found an effect only before controlling for phylogenetic autocorrelation, Walter et al. (4) only with one of six measures of gender inequality, and two investigations found no effect (1, 26). Further, consistent with the standard evolutionary psychological interpretation, sex differences in stated partner preferences for beauty are not smaller in countries with higher gender equality (1, 3, 4, 24, 26) and, in fact, they may even become larger (2).

Individual-level cross-sectional analyses have also been conducted to test whether sex-differentiated stated partner preferences are downstream of culturally assigned provider-homemaker social roles. These typically test whether women who are more able or inclined to provide have reduced ideal partner preferences for resources and stronger desire for young, handsome men, and whether men who are less able or inclined to provide are more attracted to older women with resources [as predicted prima facie by sociocultural explanations; (32, 34)]. Consistent with biosocial role theory, this research suggests that richer and more economically independent women report caring proportionately less about a man’s finances compared to his other traits, including his beauty (35–37). Further, heterosexuals who anticipate that the woman in their relationship will fulfill a greater provisioning role tend to report less sex-typical stated partner preferences (28, 38).

In contrast to biosocial role theory, poorer men do not have stronger stated partner preferences for resources than richer men, and it appears poorer women actually have weaker stated partner preferences for resources than richer women [(1, 36, 39–45), but see ref. 46]. Biosocial role theorists have argued that there is an issue with causality, however, as richer women differ from poorer women in a variety of plausibly confounding ways (14, 24). For instance, richer women seem to express higher standards for romantic partners in general, not just with regard to wealth (35, 46). The same has been said by some evolutionary psychologists about the cross-cultural literature—for instance, the nations that tend to have small age-gaps and low gender inequality (e.g., the core Anglosphere) are culturally similar in far more ways than just these two variables, and these additional similarities may be insufficiently controlled for in the relevant analyses (26).

These criticisms suggest a place for experimental tests, but such tests include only three relevant articles of which we are aware (38, 47, 48), none of which manipulated gender economic inequality or individual income, the two main factors for which confounds are commonly argued to be likely. This need for experimental investigation has become even more pressing, recently, as Berggren and Bergh (29) have cast doubt on the inferences drawn from the cross-cultural literature on this topic. Specifically, they conclude that cross-cultural studies of gender inequality’s relationship with psychological sex differences do not sufficiently account for Simpson’s paradox, a phenomenon where confounding variables or aggregation choices cause associations between variables to differ within and between clusters of data (in this case, they find the association between gender equality and sex differences differs within and between cultural clusters). Here, we provide a needed experimental contribution to this literature by manipulating the key variables at play: gender economic inequality and personal income.

The lack of clear support for either evolutionary psychological or biosocial role theories of sex differences in partner preferences also suggests a need for theoretical revision, which we hope to contribute by utilizing ideas from another discipline: behavioral ecology. The core premise of human behavioral ecology is that human behavior flexibly adapts to its ecology in the service of fitness; inclusive genetic contribution to future generations. Since it is usually not practical to measure actual genetic contribution to future generations in humans, fitness is generally assessed through intermediary goals, such as producing offspring, acquiring resources, mating, and so on, as well as assisting kin with similar goals (49). Human behavioral ecology is distinct for its explicit expectation that humans flexibly adjust their behavior to modern environments in an adaptive way, and its agnosticism about mechanisms (49). Biosocial role theorists and evolutionary psychologists are more commital about mechanisms; for instance, evolutionary psychologists often predict variation caused by evolved psychological adaptations to variable environmental cues familiar to our ancestors (26), and biosocial role theorists put forward a highly complex historical-psychological mechanistic account of the origin of certain sex differences (24). Behavioral ecology’s agnosticism allows it to be simultaneously compatible, in principal, with either biosocial role theory or evolutionary psychology (which are incompatible with one another due to their contradictory mechanistic and historical claims) while remaining distinguishable for its focus on how behaviors make strategic sense in the present.

Under this view, if an environmental feature makes preferring a particular trait in a romantic partner more or less advantageous for fitness or its proxies (in this study, pairing with a mate and avoiding poverty), it is expected to be preferred more or less in parallel. In this case, modeling indicates that hypergynous partner preferences—where women prefer to partner with men who are richer than themselves, and vice versa—worsen the pairing prospects of high-income women and low-income men (21). It also seems reasonable to expect that the desire for a mate with resources would be more urgent for individuals who have less money, and more actionable for individuals whose desired sex has more money (21). These predicted effects could plausibly interact, as a person whose desired sex is rich enough might face no tradeoff for selecting for resources even if they themselves are rich, and a person whose desired sex is poor cannot pragmatically select for resources in mates regardless of the strength of their personal need. Another aspect of this view is that if a feature of the ecology has little bearing on an ideal partner preference’s adaptiveness, the preference is not predicted to be altered. This is relevant as while personal and gendered economics change the strategic value of many partner preferences (e.g., ambition, financial security, a good job, older age, and likely also intelligence and homemaking skills), from this view, unlike biosocial role theory, it is unclear why someone’s preference for beauty would adjust to the economy, except perhaps as a relative priority.

Recent evidence for this theoretical view comes from Malovicki-Yaffe et al.’s (6, 7) research on conservative Haredi Jews, a group where women are approximately 10.7% more likely to be breadwinners than men.^§^ This is the only market culture we are aware of in which gender economic inequality is reversed and mate preferences have been measured. Consistent with our strategic perspective, normal sex differences in mate preferences for resources are reversed in this culture, such that men have stronger ideal partner preferences for resources than women, whereas traditional sex differences in mate preferences for age and beauty remain. While Malovicki-Yaffe and her colleagues appear open to the strategic influence of women’s resource-control playing a role (6, p. 624, p. 629), they primarily argue that the reason sex differences in mate preferences for resources are reversed in this population is not because the current adaptive value of the preferences is reversed per se, but mainly because women have an innate evolved stronger mate preference for status, and men derive status in this community from religious scholarship rather than resource procurement (6, 7). Essentially, they argue the traditional evolved psychological sex differences all remain, but they are shaped by the local culture. This result and their interpretation further suggest a need for experimental work, as it could be mainly the different source of men’s status among Haredi Jews, rather than the reversal of gendered resource holdings itself, that has caused this reversal in sex differences in mate preferences for resources.

While our purely strategic view is uncommon in this debate, it is not without precedent. Similar ideas with partially overlapping predictions and theory (e.g., structural powerlessness, evoked culture) have been explored by other scholars (8, 11, 26, 35, 48, 50, 51). Indeed, human behavioral ecologists often state that sex differences in preferences for resource-relevant traits in romantic partners adapt based on women’s material productivity (51, 52). However, they base this conclusion on single-site ethnographic observations [e.g., of the Kwoma (53, p. 72)], and surveys (e.g., of the Shuar: 5) that document men desiring productive women in ecologies where women procure ample resources. These cases are often compelling, but they do not associate men’s romantic preferences with women’s resource procurement in a generalizable sense.

Here, we test this behavioral ecological view, which we believe combines the strengths of both adaptationist and sociocultural perspectives. In a preregistered experiment, we utilize Harmon-Jones et al.’s (54) Stamola paradigm to place participants in one of five different virtual environments that vary in local gender economic inequality, in which the participant is randomly assigned to one of five different income levels. We then test predictions informed by behavioral ecology regarding how these ecological variables predict individual ideal partner preferences. We chose to test this view using this experimental design for three reasons: First, none of the experimental papers we are aware of (38, 47, 48) manipulated income or gender economic inequality, two factors which have both been argued to be heavily confounded in naturally varying populations (24, 26, 29). Second, men have more money than women in almost every market culture and subculture (14), so an experimental design is the only way to test the effects of full reversals in gender economic inequality.

We preregistered the following hypotheses: when individuals of either sex are poorer or in environments where the opposite sex is relatively richer than their sex, they will exhibit a stronger ideal partner preference for resource-related traits (Hypothesis 1) and a preference for relatively older mates, as older mates generally have more resources (Hypothesis 2). They will also place higher importance on a mate being better off, financially, than themselves (i.e., hypergamy; Hypothesis 3). In addition to predicting main effects for income, and the interaction between gender and gender economic inequality as noted above, for all hypotheses we further predicted a three-way interaction between gender, income, and gender economic inequality, such that poor people in gender unequal environments that disadvantage them will be especially likely to exhibit these preferences. Finally, as a test of discriminant validity, we test whether these same patterns were evident in partner preferences for physical attractiveness.

## Methods

All aspects of our methodology are available on the OSF, including our preregistration (https://doi.org/10.17605/OSF.IO/C26BZ), survey, data, and code (https://osf.io/v95xd) (55). After providing informed consent, participants entered one of 45 versions of a virtual society called “Stamola,” which varied by gender economic inequality (the ratio of women’s earnings to men’s; five levels: 0.5, 0.6667, 1.0, 1.5, 2.0) and income (one’s earning percentile within one’s sex; five levels: 10th, 30th, 50th, 70th, 90th), and were specific to the participant’s sex (two levels: male, female). There were 45 versions, not 50, because the condition of perfect equality was the same across sexes. A table listing the manipulation combinations, including links to watch the video manipulations, is in *SI Appendix*, along with all of our survey questions in the order they appeared to participants.

**Fig. 1.**
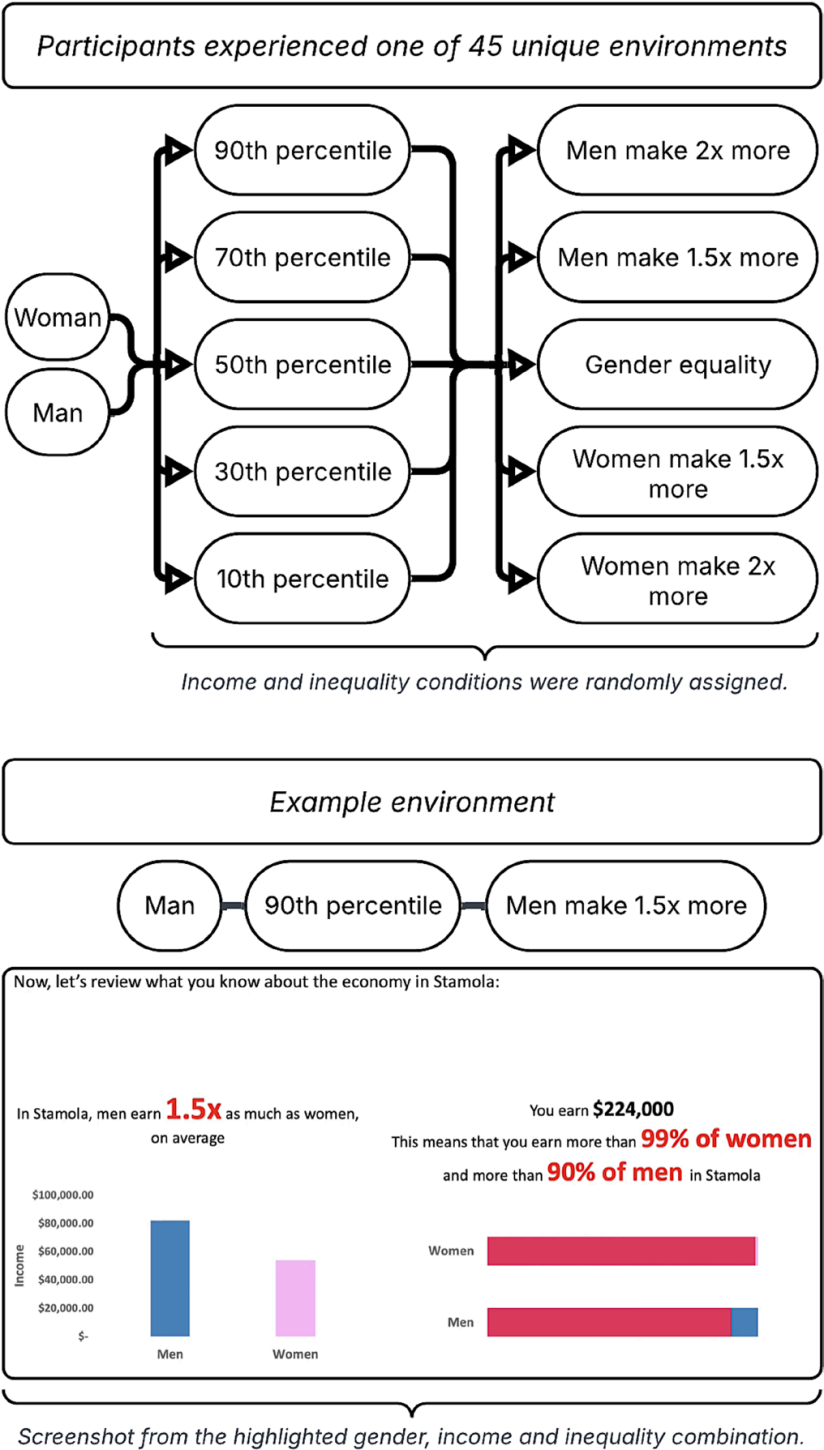
Chart depicting all gender, income, and gender economic inequality conditions, with a highlighted example of a man assigned to a 90th percentile income for his gender, and a gender economic inequality condition where men make 1.5 times more than women. *Note.* While there may appear to be 50 possible versions of Stamola, there are only 45 unique combinations, as the five gender equal conditions do not differ between men and women.

When participants entered Stamola, they were informed of the average income for women and the average income for men (rounded to the nearest thousand dollars), the degree of local gender economic inequality, their income, their income percentile within their sex, and their income percentile relative to the opposite sex. By design, our manipulation only varied participant income percentile within their sex and local gender economic inequality—by nature, though, this also varied average income for men and women, participant income, and participant income percentile relative to the opposite sex, as these variables were perfectly yoked to the combination of one’s personal within-sex income percentile and local gender economic inequality. After being given this information, they reflected qualitatively in an open-text box on what they would want in a partner in Stamola and were informed that their relationship status is single, before being surveyed quantitatively on their long-term partner preferences in Stamola. A chart depicting all gender, income, and gender economic inequality conditions with a screenshot from a highlighted example version of Stamola, is viewable in Fig. 1.

Eight-hundred and seven participants (*M*_age_ = 31.83, *SD* = 7.29, 399 men, 395 women, 13 other) were recruited on Prolific Academic and consented to participate for approximately £1.25. We recruited participants in the core Anglosphere, and prescreened participants for English fluency, as these participants were likely to be both comfortable with English and familiar with heterosexual relationships where the woman is the primary breadwinner. We also prescreened participants for age between 18 and 45 y old, as our hypotheses are connected to reproduction, and this approximates the natural reproductive window for humans (56).

After the experimental manipulation, participants completed attention and comprehension checks concerning their assigned income and gender economic inequality ratio. One-hundred participants who incorrectly answered one or more of these checks were excluded, leaving *n* = 707 (*M*_age_ = 31.74, *SD* = 7.29). Participants were then excluded from analyses if they were not cisgender and predominantly heterosexual, leaving *n* = 614 (*M*_age_ = 32.14, *SD* = 7.28). An additional 12 participants were excluded due to missing data for our key outcome variables, leaving *n =* 602 (*M*_age_ = 32.16, *SD* = 7.30, 312 men, 290 women). Our final sample included participants residing in the United Kingdom (67.6%, *n* = 406), Canada (20.10%, *n* = 121), the United States (9.15%, *n* = 55), Australia (2.33%, *n* = 14), and New Zealand (0.83%, *n* = 5).^¶^

For conceptual replication, we tested predictions concerning partner preferences for resources with four different outcome variables. First, participants indicated how important it would be for their long-term partner in Stamola to be better off than them financially, i.e., hypergamy (0 = “Not at all important”, 10 = “Extremely important”). Second, participants indicated their ideal age for their partner in Stamola as a function of their own age, ranging from 30 or more years younger (−30) to about the same age (0) to 30 or more years older (26). Third, participants rated 35 traits from Eastwick et al.’s (57) ideal partner preference inventory (0 = “Not at all desirable”, 10 = “Highly desirable”). Following Eastwick et al.’s (57) procedure, rated resource preferences were averaged ratings of “Ambitious,” “Financially secure,” and “Good job.” After these measures, participants were then reminded of their income and their local gender inequality, and asked to once again reflect on how this might affect their romantic relationships and preferences.

As our fourth preregistered outcome measure, participants ranked their resource preferences. They ranked the desirability of three resource-relevant traits, three beauty-relevant traits, and eight other traits found by Eastwick et al. (57) to be similarly desirable to these six traits, to maximize variability. This approach was necessary so we could test whether participants’ preference for resources represented a sacrifice of other preferences. We also operationalized rated and ranked preferences for physical attractiveness using the same methodology as outlined above, but for the traits “Attractive,” “Sexy,” and “Nice body.”

Participants next completed Buss’s (1) mate preference inventory. Its 4-point Likert design and traits are outdated, but several cross-cultural analyses have now used it, and so we included it for history-of-science purposes. Different research teams have made different choices, but for simplicity, we analyzed Buss’s (1) “good financial prospect” and “ambition-industriousness” items separately. Finally, participants answered four seven-point Likert items modified from Neel et al. (58), which we used to measure participants’ enthusiasm for finding a mate, as perhaps people become less enthusiastic when their baseline preferences for hypergyny are less realizable.

We used OpenAI’s ChatGPT for assistance with coding and created a custom GPT to discuss biosocial role theory and evolutionary psychology perspectives; this was to supplement our extensive discussions with evolutionary and social psychologists, in an effort to ensure that all perspectives were accurately discussed in our work. We reviewed, edited, and thoroughly checked any line of code written by AI, and take full responsibility for its content. No part of this manuscript was written by AI, and no analyses were performed directly by AI. This study was approved by the UNSW Human Research Ethics Committee.

### Analyses.

We preregistered to test hypotheses using linear regressions (i.e., a three-way interaction between gender, gender inequality, and income) and visualize them using nonparametric thin-plate splines. We then conducted ex-post-facto *t*-tests to document sex differences in partner preferences in each of the five gender economic inequality conditions, Pearson’s correlations to test our assumption that partner preferences for age relate to partner preferences for resources, and additional regressions to better understand our ranking results, as well as men’s and women’s results, separately. All analyses were run as preregistered unless explicitly noted otherwise.

## Results

### Do Partner Preferences for Resources Adapt to Income and Gender Economic Inequality?

In support of our first preregistered hypothesis, poorer participants found traits associated with access to resources more desirable in mates, both in rated (β = −0.09, *b* = −0.97, *SE* = 0.34, *t*(594) = −2.84, *P* = 0.005^**^) and ranked (β = −0.18, *b* = −1.86, *SE* = 0.51, *t*(593) = −3.62, *P* < 0.001^***^) preferences. Further, there was an interaction between gender and gender inequality, such that people felt a relatively stronger desire for resource-related traits in mates when gender economic inequality was less favorable toward their gender, again both in rated (β = −0.10, *b* = −0.68, *SE* = 0.26, *t*(594) = −2.58, *P* = 0.010^**^) and ranked (β = −0.15, *b* = −1.57, *SE* = 0.40, *t*(593) = −3.97, *P* < 0.001^***^) preferences. (For a comparison of mate preference rankings between maximally female-biased and male-biased gender economic inequality conditions, see *SI Appendix*.) However, the predicted three-way interaction between gender, gender inequality, and income, was found in neither rated (β = 0.01, *b* = 0.27, *SE* = 0.93, *t*(594) = 0.29, *P* = 0.77) nor ranked (β = 0.02, *b* = 0.77, *SE* = 1.39, *t*(593) = 0.55, *P* = 0.58) preferences.

**Fig. 2.**
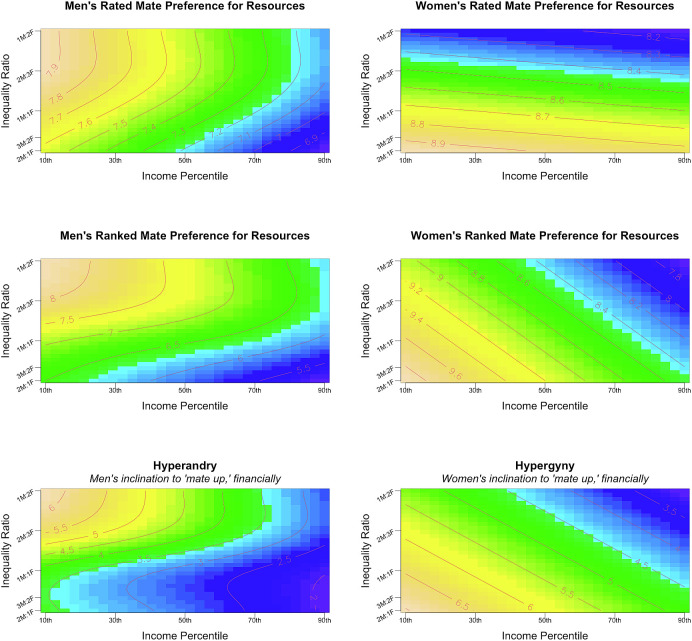
Thin-plate splines visualizing how men’s and women’s partner preferences for resources adapt to gender economic inequality and income. *Note.* These plots can be read like heat maps. Warmer tones represent higher ratings and rankings for the traits in question; cooler tones represent lower ratings and rankings for the traits in question. Contour lines represent the anticipated value of the trait along said contour (e.g., a value of “8” in rated partner preferences or hypergamy indicates that participants along this contour line rated it approximately seven out of 10; a value of “8” in rankings indicates participants along this contour line ranked resource-relevant traits approximately eighth lowest—that is, seventh highest—on average, out of the 14 traits). The y-axis displays gender economic inequality measured as a fraction of women’s relative to men’s income, such that higher values represent women being richer (e.g., “2 M:1F” is where women make half of what men make, “1 M:2F” is where women make twice as much as men).

Consistent with our preregistered hypothesis regarding hypergamy, poorer participants placed a higher importance on a partner being better off than themselves financially (β = −0.26, *b* = −2.84, *SE* = 0.52, *t*(594) = −5.41, *P* < 0.001^***^). Further, there was an interaction between gender and inequality ratio such that when men were richer, women placed a higher importance on dating/marrying up in socioeconomic status than they did when men were poorer, and when women were richer, men placed a higher importance on dating/marrying up in socioeconomic status than they did when women were poorer (β = −0.22, *b* = −2.47, *SE* = 0.41, *t*(594) = −6.08, *P* < 0.001^***^). But, once again, the predicted three-way interaction between gender, gender inequality ratio, and income was absent (β = 0.01, *b* = 0.39, *SE* = 1.43, *t*(594) = 0.27, *P* = 0.78). These results are visualized in Fig. 2.

**Fig. 3.**
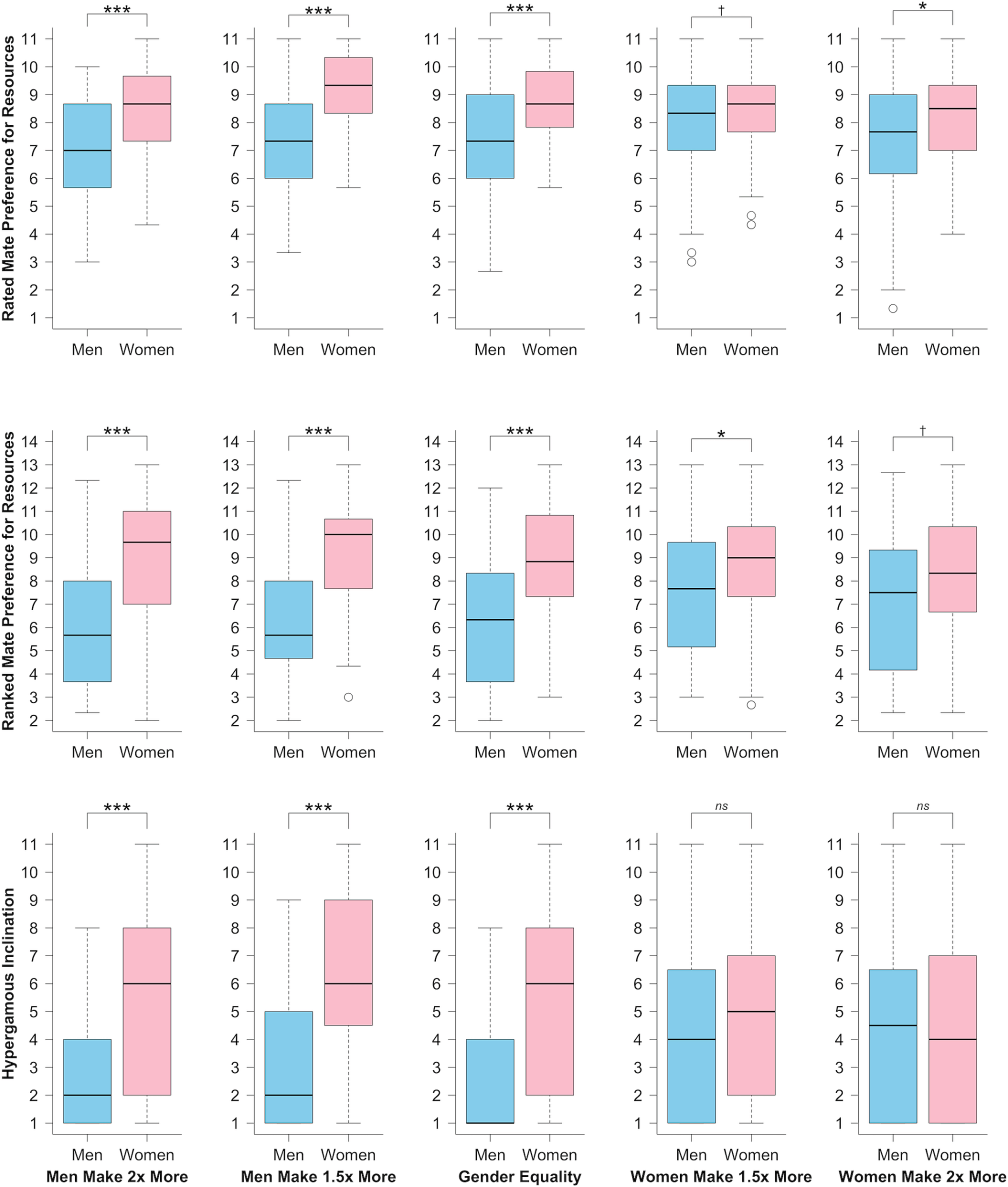
Boxplots visualizing how sex differences in partner preferences for resources lessen as women control more resources. ^†^*P* < 0.1, *P < 0.05, **P < 0.01, ***P < 0.001, significance values equate to ex post-facto *t* tests.

Ex post facto exploratory *t* tests—derived from the inequality by gender interaction—revealed that the normally expected sex differences in partner preferences for resources and hypergamous inclinations were less consistent and, in the latter case, absent where women made more money than men (Fig. 3). We preregistered that we would prioritize our Eastwick et al. (57) results over our Buss (1) results wherever results differed. Results exhibited the same pattern for Buss’s (1) “Good financial prospect” trait as for Eastwick et al.’s (57) resource composite, but with no significant effects for “Ambition-industriousness.” For detailed reporting on the Buss (1) measures, see *SI Appendix*.

### Do Partner Preferences for Age Adapt to Income and Gender Economic Inequality?

Contrary to our preregistered hypothesis regarding age preferences, there was no significant association between age gap preferences and income (β = −0.01, *b* = −0.22, *SE* = 0.59, *t*(594) = −0.37, *P* = 0.71), no interaction between gender and inequality ratio in predicting age gap preferences (β = 0.04, *b* = 0.51, *SE* = 0.46, *t*(594) = 1.10, *P* = 0.27), and no three-way interaction between gender, income, and inequality ratio (β = 0.00, *b* = 0.18, *SE* = 1.61, *t*(594) = 0.11, *P* = 0.91).

Due to these null results, we conducted ex post facto exploratory analyses to investigate whether age gap preferences statistically covaried with resource preferences in our sample. Given the theoretical logic underlying our hypotheses, age gap preferences were expected to be affected only insofar as older age was a proxy for resource holdings. Among men, there was no correlation between age preferences and rated resource preferences (*r*(310) = 0.00, *P* = 0.98), ranked resource preferences (*r*(310) = 0.01, *P* = 0.82), or hyperandry (*r*(310) = −0.02, *P* = 0.67). In women, there were small, significant positive correlations between rated resource preferences and preference for older age, as well as hypergyny and preference for older age (rated resource preferences: *r*(288) = 0.16, *P* = 0.007^**^; hypergamy, *r*(288) = 0.17, *P* = 0.004^**^), though the correlation between age gap preferences and ranked resource preferences was not significant (r(287) = 0.07, *P* = 0.25). Broadly, these results suggest that women’s partner preferences for older age and resources are somewhat related, but men’s partner preferences for age are not informed by their partner preferences for resources.

### Planned Exploratory Analyses.

Participants did not rate or rank physical attractiveness as more desirable in potential mates when they were richer (rated preferences: β = 0.03, *b* = 0.17, *SE* = 0.30, *t*(594) = 0.56, *P* = 0.58; ranked preferences: β = 0.04, *b* = 0.76, *SE* = 0.50, *t*(593) = 1.52, *P* = 0.13), and gender and gender economic inequality did not interact to predict rated preferences for beauty (β = 0.08, *b* = 0.45, *SE* = 0.23, *t*(594) = 1.95, *P* = 0.051^†^). While there was a significant interaction between gender and inequality ratio in predicting ranked beauty preferences (β = 0.12, *b* = 1.22, *SE* = 0.39, *t*(593) = 3.16, *P* = 0.002^**^), it was not robust to controlling for ranked resource preferences in an ex post facto regression (β = 0.06, *b* = 0.64, *SE* = 0.36, *t*(592) = 1.76, *P* = 0.079^†^). Conversely, the effect of income on ranked resource preferences remained significant after controlling for ranked beauty preferences (β = −0.16, *b* = −1.56, *SE* = 0.48, *t*(592) = −3.29, *P* = 0.001^**^), as did the interaction between gender and inequality ratio (β = −0.10, *b* = −1.10, *SE* = 0.37, *t*(592) = −2.97, *P* = 0.003^**^). This pattern suggests that the effect of gender and inequality ratio on ranked beauty preferences was mainly an artifact of real movement in ranked resource preferences. Last, there were no significant effects on our mate-seeking measure; for detailed reporting on these exploratory analyses, see *SI Appendix*.

## Discussion

For a generation, scholars from across the social sciences have debated whether observed sex differences in stated partner preferences are best explained primarily with reference to human nature or gender inequality. The perspective tested here—that humans will flexibly adjust their romantic preferences to the current environment in pursuit of fitness—focuses on how human nature interfaces with gender inequality. In this experiment, poorer individuals more strongly preferred mates with traits linked to resource access (e.g., having a good job) and placed a higher importance on obtaining a partner financially better off than themselves. The same pattern was also evident in gender economic inequality, with poorer individuals exhibiting stronger ideal partner preferences for resources. These effects were such that, in conditions where women had more resources, the normal sex differences between men and women became trivial, and in the case of hypergamy (for which manipulation effects were strongest), disappeared entirely. Yet, ideal partner preferences for age were surprisingly unaffected, and the predicted three-way interaction between income, gender, and gender economic inequality was not detected.

Experimental manipulation presents the surest way to circumvent three serious concerns with the past literature. Namely, i) that more financially successful women’s stronger ideal partner preferences for resources may be an artifact of natural confounds (24); ii) that the observed cross-cultural relationships between gender economic inequality and ideal partner preferences may be spurious, examples of autocorrelation and Simpson’s paradox (26, 29); and iii) that inequality-driven hypotheses cannot be fairly tested cross-culturally because gender inequality is male-biased worldwide (14). Such concerns cannot explain—nor explain away—our results.

The effects we report also open up the possibility that other observed psychological sex differences in humans may not be primarily due to sex-differentiated genetics but a downstream effect of an adaptive response by both sexes to environmental circumstances that alter the strategic valence of the psychological feature.^#^ In this case, it is possible observed sex differences in ideal partner preferences for resources are an adaptive response to widespread male-biased gender economic inequality. (Though, perhaps, ideal partner preferences for external resources are, at baseline, more likely to be adaptive for women to compensate the costs to material productivity inherent to pregnancy and lactation.) The observed adaptive pattern aligns well with predictions from theory and past formal modeling (21), but such predictions also seem reachable via general reasoning: if one is poor, money is a higher priority, and if one’s desired sex has more money, pairing becomes a more reasonable route to wealth. Conversely, if one is wealthy, or one’s desired sex has less money, a stubborn preference for mating-up financially would limit one’s pairing opportunities.

Illustrating this, many men and women in our sample seemed aware of the function of their strategic flexibility, consciously prioritizing resources in mates when they were in financial need and shifting to focus on other traits when they could afford to. As one low-income man in a virtual town where women were richer wrote: “I would want a partner who makes a lot of money to help support my sad wages.” And, as one high-income woman in a gender-egalitarian version of Stamola put it: “I think finances would matter less to me in a partner since I could easily support myself or both of us easily. I would be more concerned about a partner who actually loves me... someone who showed that in other ways that they contribute to the relationship and its success that aren’t money related.”

This willingness to recalibrate romantic desires can be read as good news in light of ongoing historical trends. As women steadily increase their command of resources relative to men across much of the world, preferences for hypergyny must weaken or pairing probability will decrease (21). In these data, the widely publicized concern (19, 20, 59) that people are obdurate in their preference for hypergyny—and, so, unlikely to select from their available romantic options in a more gender equal world—is empirically not supported.

This documented recalibration of partner preferences may also incentivize recalibration in behavior among those subject to these preferences. Buss [(1), p. 41] suggested that one reason why men stably obtain more resources across cultures may be because ancestral women stably preferred men with “external resources” to provide, and so there was “greater selective pressure placed on males than on females to acquire those resources.” Our study suggests some causality flows in the opposite direction and calls into further question the stability of sex-differentiation in these preferences. Nevertheless, the suggested causal connection between preferences and effort is reasonable and, we would expect, likely as flexible as the preferences themselves. If we are correct on this latter point, it stands to reason that as one sex calibrates their preferences to their ecology, those that desire said sex will calibrate their phenotype (their behavior, appearance, etc.) to these preferences in order to better attract them. While we would expect this preference-phenotype calibration to occur for any modifiable feature that mates might care about, in this case, the implication would be that women might compete more vigorously for resources—and signal them more ostentatiously—as their resources become more desirable to men. Further, men may compete less vigorously for resources as women reward them less for it, instead allocating more effort in other domains, such as beautification and child-rearing. For example, in the US, since the 1960s, men have dramatically upscaled their beautification (60, 61) and tripled their time spent on parenting efforts (62) over the same time period that American women have seen dramatic gains in their control of resources. In part, this may reflect an attempt by men to calibrate their offerings to women who no longer rely on their money (60–62).

Naturally, some scholars will wonder whether our experiment reflects what happens in real-world cash economies. For instance, while we think it is unlikely that participants would infer our theoretical model and upregulate and downregulate their partner preferences accordingly, some may worry there are demand effects, and others may be concerned that the preferences participants think they would have in theory are not the preferences they would actually have in practice. Here, it is worth noting that the patterns we observed align with past studies suggesting individuals report more interest in mates with resources i) where the desired gender has relatively more resources (2, 24, 25), ii) if the individual has less control of resources (36, 37), iii) where the local cost-of-living is higher (13, 41), iv) if the individual expects their mate to fulfill a provisioning role (46, 47, 63), and v) when the individual is experimentally manipulated to consider resource scarcity (35, 64, 65). They also align with vi) the limited longitudinal literature on partner preferences in gender egalitarian nations, which shows convergence in sex-differentiated stated partner preferences over time (66–68) and vii) data from the only real-world market culture we know of where women engage in more provisioning than men in which mate preferences have been measured. Among conservative Haredi Jews, where women are more often the breadwinner than men, men say they are more attracted to financially successful mates than women while sex differences in stated partner preferences for beauty and age remain unaffected (6, 7).

### Compatibility with Evolutionary Theory.

Evolutionary psychological scholars often argue that women’s hypergynous preferences are stable facets of our mating psychology, which would lead one to anticipate that successful women will struggle to find a partner, as there are less available men who are more successful than themselves who can satisfy their preference to mate up (69). So, in an experiment like ours, the conventional evolutionary psychological prediction might be opposed to our findings, with an expectation that richer women would upregulate—rather than downregulate—their mate preferences for resources.

However, the possibility that human mate preferences for resources adapt to the environment has been considered by past evolutionary psychological scholars (26, 31, 35, 36, 48, 70, 71), so we are optimistic our perspective will be seen as compatible, rather than competing, with evolutionary psychology. It is plausible some will find it dubious that humans would evolve or retain major flexibility in preferences for romantic partners with resources, on the supposition that mostly female provisioning is historically unusual. This supposition, however, is unsupported by the anthropological literature. Foragers, who are commonly used as loose proxies for our ancestors, show tremendous flexibility in gendered and individual resource-acquisition (26, 72–74). For instance, Marlowe’s (72) sample of 161 such societies reports high variability in gender caloric inequality, with women producing upward of 1.5 times as much food as men among Efé (DR Congo), G/Wi (Botswana), and Arrernte foragers (Australia), to take only a few examples. This variation appears due to ecology more so than culturally assigned roles. Across human societies, males typically hunt more than females, and females typically gather more than males, and these activities vary in their relative productivity across ecologies (72, 75). For instance, among the Paliyan of South India, 75% of food is procured by women, likely because they live in montane rainforests, where the primary food source—wild yams—is gathered (76).

The *Homo* genus evolved in Africa over a diverse range of habitats which differentially favor hunted vs. gathered nutrition (72, 77), and so the sexes likely varied in their relative productivity in our ancestry. So, given the anthropological literature, if we were to take a traditional evolutionary psychological view and assess our ancestral past’s likely selection pressures, we would argue that the anthropological literature on hunter-gatherers lends itself easily to adaptations for psychological flexibility in the sex-differentiation of mate preferences for resources (26). Though, to be clear, the human behavioral ecological approach is nested in the present relationships between humans and their environments, rather than the influence of the likely conditions of our ancestry on modern human psychology (for full discussion of the theoretical differences between these fields, see ref. 49).

### Compatibility with Biosocial Role Theory.

These data can also be interpreted as supportive of biosocial role theory, because they lend credence to the idea that sex differences in ideal partner preferences for resources are downstream of gender inequality. Further, they provide experimental support of their core prediction that sex differences in partner preferences for resources are due to gender inequality, in a way that circumvents common critiques of prior theoretical support. And, for clarity, our perspective is in principle compatible; we would agree with biosocial role theorists that social roles are relevant to partner preferences (especially insofar as they have implications for fitness). However, the behavioral ecological perspective does diverge from biosocial role theory in a few key ways.

First, the human behavioral ecological perspective makes no mechanistic or historical claims. Biosocial role theory posits that physiological differences led to the cultural siphoning of men and women into different social roles, which developed into a provider-homemaker dichotomy in market economies that incentivized different preferences for romantic partners (this could be true, but the argument for this is not made based on robust historical evidence, see refs. 14 and 24). Second, while we would argue these two perspectives are compatible in principle, biosocial role theorists have clarified that their theory is put forward in disagreement with behavioral ecology’s fundamental precept of fitness optimization [(24), p. 421]. They instead argue humans maximize “life outcomes,” “benefits,” “well-being,” and “utility” (14, 47), though it is unclear how the human mind could have evolved to maximize such things unless doing so increased fitness. Third, beauty preferences responded to our manipulation only relative to resources. One systemic issue in the biosocial role theory literature is that when sex differences in partner preferences for beauty reduce with gender-equality-relevant variables, it is highlighted as ratification [(14, p. 356), (78, p. 95)], but when they are amplified or unaffected, it is framed as a trivial surprise (e.g., ref. 2). One advantage of a behavioral ecological perspective is that it does not make this largely unsupported prediction.

### Discrepancies with Prior Literature and Predictions.

At first glance, our results appear contrary to findings that show richer women say they want even richer mates [(1, 36, 39–45), but see ref. 46], and that poorer men do not generally express more concern about a mate’s resources (1, 39–43, 45). However, our findings align well with the common claim that the reason richer women in organically varying populations state a preference for richer mates is not due to their wealth itself, but due to confounding variables (14, 47). For instance, in the wild, richer women differ from poorer women in a few psychological traits, including, notably, expressing higher standards for mates across the board (35, 46). They also have more proximity and social access to richer men, such that this preference is more realistic (68). Confounds like this are mostly nonissues for an experimental design like ours. While challenging to conceive, a natural experiment (e.g., a study of how a recession changes romantic partner preferences) might help further reveal the impact of resources *eo ipso*.

We did not find any of our predicted three-way interactions between gender economic inequality, income, and gender. This could suggest that income and gender income inequality exert largely separate, linear effects on partner preferences for resources, or that it was overly ambitious for us to expect to find a complex three-way interaction from a brief manipulation. More surprising, given that older age is associated with greater resources, and given how consistently age gaps reduce with gender equality in the real world (4), was that our predictions around age preferences were not upheld. It seems plausible that the reason age gaps decrease with rising gender equality in past research, but not ours, is that the documented relationship between age gaps and gender inequality is not caused by changes in preferences. Perhaps, instead, it is caused by changes in power (10), whereby men lose the ability to obtain the large downward age gaps they desire in old age (9).

Relevant to the above point, we were incorrect in an underlying assumption: men’s stated partner preferences for age did not correlate with their stated partner preferences for resources. This is consistent with men’s cross-culturally universal preference for young women being an evolved trait driven by age-related infertility, a stable strategic factor unrelated to resources (9). Women’s stated partner preferences for resources mostly correlated with their stated partner preferences for older age, as expected, but this correlation was quite small. Perhaps, since our manipulation made no mention of age’s relevance to resources, women did not consider the connection, or perhaps age preferences are not powerfully driven by age’s association with resources. It is also possible that there is no true discrepancy with past literature, and that the well-documented closing of age gaps with rising gender equality (4) is mostly downstream of women deprioritizing resources—they end up opting for younger men mainly as a second-order consequence of caring less about a mate’s money.

## Conclusion

Scholars from various disciplines have long debated whether and why gender economic inequality and income impact partner preferences, relying almost entirely on correlational data to make their arguments. These correlational methods have led to controversy and critique, with concerns on both sides regarding natural confounds leading to spurious conclusions. To advance this debate, we present an experimental test of the effects of gender economic inequality and income on ideal partner preferences. Informed by behavioral ecology, we proposed and found that ideal partner preferences for resource-relevant traits depend on the strategic utility of partner preferences for resources in one’s ecology. This was true in participants’ rated and ranked ideal partner preferences for resources and in their importance placed on “dating/marrying up,” financially (ideal partner preferences for physical attractiveness were largely unaffected, demonstrating discriminant validity). We encourage researchers to adopt and pioneer new experimental approaches to investigating the causes of psychological sex differences, and to consider romantic partner preferences as the results of simple, strategic responses to fitness-relevant environmental conditions.

## Supplementary Material

Appendix 01 (PDF)

## Data Availability

Anonymized .csv data have been deposited in the Open Science Framework (https://osf.io/v95xd) (55).

## Footnotes

^*^Sex differences in partner preferences for resources and physical attractiveness are generally not found in studies that measure revealed partner preferences, such as speed-dating outcomes (57, 79). While sex-differentiated age preferences are more consistently found across study designs (9), here, too, there are exceptions (10). Our study focuses on what people say they want, i.e., their stated or “ideal” partner preferences (57).

^†^For example, a podcast interview centered on this concern has 4 million views (59).

^‡^A recent meta-analysis (22) suggests that women’s fertility is not inferrable from feminine morphological characteristics, despite common assumption to the contrary within evolutionary psychology. Notably, Lassek and Gaulin (34) argue that while features associated with women’s attractiveness may not cue fertility directly, they often cue nubility and nulligravidity.

^§^This estimate is based on reporting from their sample that women were breadwinners in 38.4% of participant households, men in 34.7% (7).

^¶^We cannot confirm nationality for one participant who entered their Prolific ID incorrectly, but we know they were from one of these five countries—it was not available to participants elsewhere.

^#^For instance, maybe in environments where physically attractive partners of an individual’s desired gender are abundant and therefore obtainable without significant tradeoffs, they would exhibit a stronger preference for physical attractiveness in romantic partners (80).
