# Supplementary material for: Partner preferences for resources adapt to income and gender economic inequality
Source: Proc Natl Acad Sci U S A. 2026 Mar 16;123(12):e2527295123. doi: 10.1073/pnas.2527295123 (PMC13012117; doi:10.1073/pnas.2527295123)
Supplement: Supplementary file 1 — Appendix 01 (PDF) [file pnas.2527295123.sapp.pdf]

Supporting Information for
Partner preferences for resources adapt to income and gender economic inequality

Macken Murphy [a], Sylvia K Harmon-Jones [b], Auguste G Harrington [c], Robert C Brooks [d], Khandis R Blake [a]

[a] University of Melbourne, [b] University of Wollongong, [c] New York University Abu Dhabi, [d] UNSW

Macken Murphy
Email: macken.murphy@student.unimelb.edu.au

This PDF file includes:

Supporting text
Tables S1 to S4
SI References
Survey flow
Survey questions

Supporting Information Text

This document contains the results of certain planned exploratory analyses and *post hoc* analyses referred to in our manuscript (pp. 2–4), a table containing hyperlinks to all variants of our experimental manipulation (p. 5), a table displaying men's and women's trait rankings for the most extreme conditions (p. 6), an export containing our survey flow (pp. 7–57), and all of our survey questions (pp. 58–374), which were delivered to participants on Qualtrics. For those who would like to see our survey as it appeared to participants on Qualtrics, the .qsf file is available here, along with our data and code: https://osf.io/v95xd/?view_only=19fe79612a8a4f97bffde6efad3a1be9

Results

Our manipulation affected Buss's (1) measures largely as expected for "Good financial prospect," with poorer participants rating this as more important or desirable (b = -0.43, SE = 0.17, t(593) = -2.58, p = .010*). Further, there was an interaction between gender and inequality ratio such that the desired gender having relatively more resources led to this being rated as more important or desirable (b = -0.29, SE = 0.13, t(593) = -2.23, p = .027*). However, these effects were non-significant for "Ambition-industriousness," with neither an effect of income (b = 0.10, SE = 0.17, t(593) = 0.63, p = .53) nor an interaction between gender and inequality ratio (b = -0.14, SE = 0.13, t(593) = -1.13, p = .26). Similar to our main analyses, there were no three-way interactions for "Ambition-industriousness" (b = 0.80, SE = 0.45, t(593) = 1.79, p = .075†) nor "Good financial prospect" (b = 0.07, SE = 0.45, t(593) = 0.15, p = .88).

Our manipulation did not alter individuals' inclinations towards mate-seeking. There was neither a significant effect of income (b = 0.24, SE = 0.24, t(593) = 1.01, p = .31) nor a significant interaction between gender and inequality ratio (b = -0.02, SE = 0.18, t(593) = -0.12, p = .91). Finally, as was the case for all of our analyses, there was no significant three-way interaction (b = 0.09, SE = 0.64, t(593) = 0.15, p = .88).

For simple slopes analyses exploring how the strength of the effect of income varies across measures by gender and gender economic inequality, for the main analyses where we found effects, see Table 1. *Ex post facto* exploratory t-tests revealed that the normally expected sex differences in mate preferences for resources and hypergamous inclinations were less consistent and, in the latter case, absent where women made more money than men. These are reported in Table 2. All possible manipulation conditions are shown in Table 3.

Table S1. Simple slopes analyses showing how the strength of the effect of income varies across measures by gender and gender economic inequality.

Measure	Inequality Ratio (±SD)	Estimate	SE	t-value
Rated Resource Preferences				
Men	Men wealthier (-1 SD)	-0.82	0.53	-1.56^{ns}
	Gender equality (mean)	-0.97	0.37	**-2.60****
	Women wealthier (+1 SD)	-1.12	0.53	**-2.13***
Women	Men wealthier (-1 SD)	-0.16	0.47	-0.34^{ns}
	Gender equality (mean)	-0.16	0.33	-0.50^{ns}
	Women wealthier (+1 SD)	-0.17	0.45	-0.37^{ns}
Ranked Resource Preferences				
Men	Men wealthier (-1 SD)	-1.13	0.76	-1.49^{ns}
	Gender equality (mean)	-1.87	0.54	**-3.49*****
	Women wealthier (+1 SD)	-2.62	0.76	**-3.45*****
Women	Men wealthier (-1 SD)	-1.43	0.76	-1.88†
	Gender equality (mean)	-1.74	0.52	**-3.32****
	Women wealthier (+1 SD)	-2.05	0.72	**-2.84****
Hypergamy				
Men	Men wealthier (-1 SD)	-1.70	0.66	**-2.57***
	Gender equality (mean)	-2.87	0.47	**-6.12*****
	Women wealthier (+1 SD)	-4.03	0.66	**-6.10*****
Women	Men wealthier (-1 SD)	-1.77	0.90	-1.96†
	Gender equality (mean)	-2.69	0.63	**-4.31*****
	Women wealthier (+1 SD)	-3.62	0.86	**-4.19*****

† *p* < .1, * *p* < .05, ** *p* < .01, *** *p* < .001

Table S2. Exploratory t-tests checking for sex differences in mate preferences for resources across gender economic inequality ratios.

Measure	Inequality Ratio	Men	Women	*t*
**Rated Resource Preferences**	Men earn 2x more	7.04	8.50	**-4.45*** **
	Men earn 1.5x more	7.29	9.18	**-6.40*** **
	Gender equality	7.41	8.70	**-4.48*** **
	Women earn 1.5x more	7.86	8.45	-1.94†
	Women earn 2x more	7.32	8.14	**-2.26***
**Ranked Resource Preferences**	Men earn 2x more	5.82	8.91	**-5.95*** **
	Men earn 1.5x more	6.13	9.34	**-7.72*** **
	Gender equality	6.28	9.00	**-6.30*** **
	Women earn 1.5x more	7.64	8.70	**-2.18***
	Women earn 2x more	7.10	8.20	-1.92†
**Hypergamy**	Men earn 2x more	2.79	5.56	**-5.17*** **
	Men earn 1.5x more	2.94	6.32	**-7.09*** **
	Gender equality	2.51	5.19	**-5.85*** **
	Women earn 1.5x more	4.56	4.69	-0.24 *ns*
	Women earn 2x more	4.41	4.22	0.33 *ns*

† $p < .1$, * $p < .05$, ** $p < .01$, *** $p < .001$

Table S3. Participants' percentile of income relative to members of the same gender and the opposite gender, as a function of assigned gender inequality ratio, with hyperlinks for each unique manipulation.

	Male					Female				
	Percentile of Income Relative to Members of the Same Gender									
**GEI**	10th	30th	50th	70th	90th	10th	30th	50th	70th	90th
	Percentile of Income Relative to Members of the Opposite Gender									
**2M:1F**		55th	75th	95th	99th	<1st	5th	20th	40th	60th
**3M:2F**	25th	45th	65th	85th	99th	<1st	10th	30th	50th	70th
**1M:1F**	10th	30th	50th	70th	90th	10th	30th	50th	70th	90th
**2M:3F**	<1st	10th	30th	50th	70th	25th	45th	65th	85th	99th
**1M:2F**	<1st	5th	20th	40th	60th	35th	55th	75th	95th	99th

Note. Modified table reprinted with permission from Harmon-Jones et al. (2). Participants' percentile of income relative to members of the opposite gender varied as a function of the gender inequality ratio and their percentile of income relative to members of the same gender.

Table S4. Men's and women's average ranking of 14 traits of similar desirability (see: 3) in versions of Stamola with extreme gender economic inequality (men making twice as much money as women versus women making twice as much money as men).

If men make twice as much as women		If women make twice as much as men	
**Men's trait ranking**	**Women's trait ranking**	**Men's trait ranking**	**Women's trait ranking**
1. Attractive	1. Financially secure	1. Attractive	1. Confident
2. Confident	2. Confident	2. Confident	2. Attractive
3. Open to new experiences, complex	3. Attractive	3. Sensitive	3. Open to new experiences, complex
4. Sexy	4. Ambitious	4. Sexy	4. Financially secure
5. Adventurous	5. Successful	5. Smells good	5. Ambitious
6. Nice body	6. Sensitive	6. Financially secure	6. Adventurous
7. Smells good	7. Good job	7. Ambitious	7. Successful
8. Ambitious	8. Open to new experiences, complex	8. Open to new experiences, complex	8. Good job
9. Dresses well	9. Adventurous	9. Nice body	9. Sensitive
10. Sensitive	10. Smells good	10. Adventurous	10. Extraverted, enthusiastic
11. Extraverted, enthusiastic	11. Dresses well	11. Dresses well	11. Dresses well
12. Successful	12. Nice body	12. Successful	12. Smells good
13. Financially secure	13. Sexy	13. Good job	13. Nice body
14. Good job	14. Extraverted, enthusiastic	14. Extraverted, enthusiastic	14. Sexy

Note. Only these 14 traits were ranked. Because this inventory was selected partially for similarity in desirability, the above ranking inflates the apparent differences in desirability in traits; in reality, most of these traits were quite similar in desirability across conditions. E.g., for men, there was less than a single rank of actual average difference between "Sensitive" and "Dresses well" under conditions where women made twice as much as men, even though it looks like an eight rank difference in this table.

SI References

1. D. M. Buss, Sex differences in human mate preferences: Evolutionary hypotheses tested in 37 cultures. *Behavioral and Brain Sciences* **12**, 1–14 (1989).

2. S. K. Harmon-Jones, K. R. Blake, A. G. Harrington, R. C. Brooks, Experimentally Manipulating Gender Inequality Affects Explicit and Implicit Gendered Attitudes and Self-Perceptions. [Preprint] (2025). Available at: https://osf.io/preprints/psyarxiv/4hqex_v1/.

3. P. W. Eastwick, *et al.*, A worldwide test of the predictive validity of ideal partner preference matching. *Journal of Personality and Social Psychology* **128** (2024).

Standard: Pre-screening (4 Questions)

Branch: New Branch

If

If What is your age in years? 46 Is Selected
Or What is your age in years? 47 Is Selected
Or What is your age in years? 48 Is Selected
Or What is your age in years? 49 Is Selected
Or What is your age in years? 50 Is Selected
Or What is your age in years? 51 Is Selected
Or What is your age in years? 52 Is Selected
Or What is your age in years? 53 Is Selected
Or What is your age in years? 54 Is Selected
Or What is your age in years? 55 Is Selected
Or What is your age in years? 56 Is Selected
Or What is your age in years? 57 Is Selected
Or What is your age in years? 58 Is Selected
Or What is your age in years? 59 Is Selected
Or What is your age in years? 60 Is Selected
Or What is your age in years? 61 Is Selected
Or What is your age in years? 62 Is Selected
Or What is your age in years? 63 Is Selected
Or What is your age in years? 64 Is Selected
Or What is your age in years? 65 Is Selected
Or What is your age in years? 66 Is Selected
Or What is your age in years? 67 Is Selected
Or What is your age in years? 68 Is Selected
Or What is your age in years? 69 Is Selected
Or What is your age in years? 70 Is Selected
Or What is your age in years? 71 Is Selected
Or What is your age in years? 72 Is Selected
Or What is your age in years? 73 Is Selected
Or What is your age in years? 74 Is Selected
Or What is your age in years? 75 Is Selected
Or What is your age in years? 76 Is Selected
Or What is your age in years? 77 Is Selected
Or What is your age in years? 78 Is Selected
Or What is your age in years? 79 Is Selected
Or What is your age in years? 80 Is Selected
Or What is your age in years? 81 Is Selected
Or What is your age in years? 82 Is Selected
Or What is your age in years? 83 Is Selected
Or What is your age in years? 84 Is Selected
Or What is your age in years? 85 Is Selected

Or What is your age in years? 86 Is Selected
Or What is your age in years? 87 Is Selected
Or What is your age in years? 88 Is Selected
Or What is your age in years? 89 Is Selected
Or What is your age in years? 90 Is Selected
Or What is your age in years? 91 Is Selected
Or What is your age in years? 92 Is Selected
Or What is your age in years? 93 Is Selected
Or What is your age in years? 94 Is Selected
Or What is your age in years? 95 Is Selected
Or What is your age in years? 96 Is Selected
Or What is your age in years? 97 Is Selected
Or What is your age in years? 98 Is Selected
Or What is your age in years? 99 Is Selected
Or What is your age in years? 100 Is Selected
Or Are you fluent in English? Yes Is Not Selected

EndSurvey: Advanced

EmbeddedData
ScreenedOut = 1

EmbeddedData
PROLIFIC_PIDValue will be set from Panel or URL.
sourceValue will be set from Panel or URL.
idValue will be set from Panel or URL.
workerIdValue will be set from Panel or URL.

Standard: Consent (4 Questions)

Branch: New Branch
If
If Consent Form – Participant providing own consent Declaration by the participant By checking the I... I do not agree Is Selected

EndSurvey: Advanced

EmbeddedData
DidNotConsent = 1

Standard: Demographics (3 Questions)

Branch: New Branch
If
If Please indicate your biological sex at birth Female Is Selected
And Please indicate your gender identity Woman Is Selected
AndIf

If How would you describe your sexuality? Exclusively attracted to men Is Selected
Or How would you describe your sexuality? Predominantly attracted to men, only incidentally attracted to women Is Selected
Or How would you describe your sexuality? Predominantly attracted to men, but more than incidentally attracted to women Is Selected

EmbeddedData
ltr = a serious boyfriend or a husband
samegenpeople = women
opgenperson = man
opgenpeople = men
StraightCisHet = 1

Branch: New Branch
If
If Please indicate your biological sex at birth Male Is Selected
And Please indicate your gender identity Man Is Selected
AndIf
If How would you describe your sexuality? Exclusively attracted to women Is Selected
Or How would you describe your sexuality? Predominantly attracted to women, only incidentally attracted to men Is Selected
Or How would you describe your sexuality? Predominantly attracted to women, but more than incidentally attracted to men Is Selected

EmbeddedData
ltr = a serious girlfriend or a wife
samegenpeople = men
opgenperson = woman
opgenpeople = women
StraightCisHet = 1

Branch: New Branch
If
If Please indicate your biological sex at birth Male Is Not Selected
And Please indicate your gender identity Man Is Selected
AndIf
If How would you describe your sexuality? Exclusively attracted to women Is Selected
Or How would you describe your sexuality? Predominantly attracted to women, only incidentally attracted to men Is Selected
Or How would you describe your sexuality? Predominantly attracted to women, but more than incidentally attracted to men Is Selected

EmbeddedData

ltr = a serious girlfriend or a wife
samegenpeople = men
opgenperson = woman
opgenpeople = women
StraightCisHet = 0

Branch: New Branch
If
If Please indicate your biological sex at birth Female Is Selected
And Please indicate your gender identity Woman Is Selected
AndIf
If How would you describe your sexuality? Exclusively attracted to women Is Selected
Or How would you describe your sexuality? Predominantly attracted to women, only incidentally attracted to men Is Selected
Or How would you describe your sexuality? Predominantly attracted to women, but more than incidentally attracted to men Is Selected

EmbeddedData
ltr = a serious girlfriend or a wife
samegenpeople = women
opgenperson = woman
opgenpeople = women
StraightCisHet = 0

Branch: New Branch
If
If Please indicate your biological sex at birth Female Is Not Selected
And Please indicate your gender identity Woman Is Selected
AndIf
If How would you describe your sexuality? Exclusively attracted to women Is Selected
Or How would you describe your sexuality? Predominantly attracted to women, only incidentally attracted to men Is Selected
Or How would you describe your sexuality? Predominantly attracted to women, but more than incidentally attracted to men Is Selected

EmbeddedData
ltr = a serious girlfriend or a wife
samegenpeople = women
opgenperson = woman
opgenpeople = women
StraightCisHet = 0

Branch: New Branch
If

If Please indicate your biological sex at birth Male Is Selected
And Please indicate your gender identity Man Is Selected
AndIf
If How would you describe your sexuality? Exclusively attracted to men Is Selected
Or How would you describe your sexuality? Predominantly attracted to men, only incidentally attracted to women Is Selected
Or How would you describe your sexuality? Predominantly attracted to men, but more than incidentally attracted to women Is Selected

EmbeddedData
ltr = a serious boyfriend or a husband
samegenpeople = men
opgenperson = man
opgenpeople = men
StraightCisHet = 0

Branch: New Branch
If
If Please indicate your biological sex at birth Male Is Not Selected
And Please indicate your gender identity Man Is Selected
AndIf
If How would you describe your sexuality? Exclusively attracted to men Is Selected
Or How would you describe your sexuality? Predominantly attracted to men, only incidentally attracted to women Is Selected
Or How would you describe your sexuality? Predominantly attracted to men, but more than incidentally attracted to women Is Selected

EmbeddedData
ltr = a serious boyfriend or a husband
samegenpeople = men
opgenperson = man
opgenpeople = men
StraightCisHet = 0

Branch: New Branch
If
If Please indicate your biological sex at birth Female Is Not Selected
And Please indicate your gender identity Woman Is Selected
AndIf
If How would you describe your sexuality? Exclusively attracted to men Is Selected
Or How would you describe your sexuality? Predominantly attracted to men, only incidentally attracted to women Is Selected

Or How would you describe your sexuality? Predominantly attracted to men, but more than incidentally attracted to women Is Selected

EmbeddedData
ltr = a serious boyfriend or a husband
samegenpeople = women
opgenperson = man
opgenpeople = men
StraightCisHet = 0

Branch: New Branch
If
If Please indicate your gender identity Man Is Not Selected
And Please indicate your gender identity Woman Is Not Selected
ElseIf
If How would you describe your sexuality? Equally attracted to men and women Is Selected
Or How would you describe your sexuality? Asexual (No socio-sexual contacts or reactions) Is Selected
Or How would you describe your sexuality? Pansexual (Attracted toward people regardless of sex or gender identity) Is Selected
Or How would you describe your sexuality? Other (Please specify) Is Selected
Or How would you describe your sexuality? Text Response Is Not Empty

EmbeddedData
ltr = a serious partner or a spouse
samegenpeople = people
opgenperson = person
opgenpeople = people
StraightCisHet = 0

Standard: Pre-Stamola Wake-up Call (1 Question)
Standard: Stamola Intro (1 Question)

Branch: New Branch
If
If Please indicate your gender identity Man Is Selected

BlockRandomizer: 1 - Evenly Present Elements

Block: 2M:1F 90th Male (DC) (12 Questions)

Branch: New Branch
If
If The video below will give you some information about the economy and your own financial situation... Is Displayed
AndIf

If Which of the following options best describes the average gender gap in Stamola? Men earn 2x more than women Is Not Selected
Or How does your income compare to that of men in Stamola? I earn more than 90% of men Is Not Selected
Or How does your income compare to that of women in Stamola? I earn more than 99% of women Is Not Selected

EmbeddedData
FailedComprehension = 1

Block: 2M:1F 70th Male (DC) (12 Questions)

Branch: New Branch
If
If The video below will give you some information about the economy and your own financial situation... Is Displayed
AndIf
If Which of the following options best describes the average gender gap in Stamola? Men earn 2x more than women Is Not Selected
Or How does your income compare to that of men in Stamola? I earn more than 70% of men Is Not Selected
Or How does your income compare to that of women in Stamola? I earn more than 95% of women Is Not Selected

EmbeddedData
FailedComprehension = 1

Standard: 2M:1F 50th Male (DC) (12 Questions)

Branch: New Branch
If
If The video below will give you some information about the economy and your own financial situation... Is Displayed
AndIf
If Which of the following options best describes the average gender gap in Stamola? Men earn 2x more than women Is Not Selected
Or How does your income compare to that of men in Stamola? I earn more than 50% of men Is Not Selected
Or How does your income compare to that of women in Stamola? I earn more than 75% of women Is Not Selected

EmbeddedData
FailedComprehension = 1

Block: 2M:1F 30th Male (DC) (12 Questions)

Branch: New Branch
If

If The video below will give you some information about the economy and your own financial situation... Is Displayed
AndIf
If Which of the following options best describes the average gender gap in Stamola? Men earn 2x more than women Is Not Selected
Or How does your income compare to that of men in Stamola? I earn more than 30% of men Is Not Selected
Or How does your income compare to that of women in Stamola? I earn more than 55% of women Is Not Selected

EmbeddedData
FailedComprehension = 1

Standard: 2M:1F 10th Male (DC) (12 Questions)

Branch: New Branch
If
If The video below will give you some information about the economy and your own financial situation... Is Displayed
AndIf
If Which of the following options best describes the average gender gap in Stamola? Men earn 2x more than women Is Not Selected
Or How does your income compare to that of men in Stamola? I earn more than 10% of men Is Not Selected
Or How does your income compare to that of women in Stamola? I earn more than 35% of women Is Not Selected

EmbeddedData
FailedComprehension = 1

Standard: 3M:2F 90th Male (DC) (12 Questions)

Branch: New Branch
If
If The video below will give you some information about the economy and your own financial situation... Is Displayed
AndIf
If Which of the following options best describes the average gender gap in Stamola? Men earn 1.5x more than women Is Not Selected
Or How does your income compare to that of men in Stamola? I earn more than 90% of men Is Not Selected
Or How does your income compare to that of women in Stamola? I earn more than 99% of women Is Not Selected

EmbeddedData
FailedComprehension = 1

Block: 3M:2F 70th Male (DC) (12 Questions)

Branch: New Branch
If
If The video below will give you some information about the economy and your own financial situation... Is Displayed
AndIf
If Which of the following options best describes the average gender gap in Stamola? Men earn 1.5x more than women Is Not Selected
Or How does your income compare to that of men in Stamola? I earn more than 70% of men Is Not Selected
Or How does your income compare to that of women in Stamola? I earn more than 85% of women Is Not Selected

EmbeddedData
FailedComprehension = 1

Standard: 3M:2F 50th Male (DC) (12 Questions)

Branch: New Branch
If
If The video below will give you some information about the economy and your own financial situation... Is Displayed
AndIf
If Which of the following options best describes the average gender gap in Stamola? Men earn 1.5x more than women Is Not Selected
Or How does your income compare to that of men in Stamola? I earn more than 50% of men Is Not Selected
Or How does your income compare to that of women in Stamola? I earn more than 65% of women Is Not Selected

EmbeddedData
FailedComprehension = 1

Block: 3M:2F 30th Male (DC) (12 Questions)

Branch: New Branch
If
If The video below will give you some information about the economy and your own financial situation... Is Displayed
AndIf
If Which of the following options best describes the average gender gap in Stamola? Men earn 1.5x more than women Is Not Selected
Or How does your income compare to that of men in Stamola? I earn more than 30% of men Is Not Selected

Or How does your income compare to that of women in Stamola? I earn more than 45% of women Is Not Selected

EmbeddedData
FailedComprehension = 1

Standard: 3M:2F 10th Male (DC) (12 Questions)

Branch: New Branch
If
If The video below will give you some information about the economy and your own financial situation... Is Displayed
AndIf
If Which of the following options best describes the average gender gap in Stamola? Men earn 1.5x more than women Is Not Selected
Or How does your income compare to that of men in Stamola? I earn more than 10% of men Is Not Selected
Or How does your income compare to that of women in Stamola? I earn more than 25% of women Is Not Selected

EmbeddedData
FailedComprehension = 1

Standard: 1M:1F 90th (DC) (12 Questions)

Branch: New Branch
If
If The video below will give you some information about the economy and your own financial situation... Is Displayed
AndIf
If Which of the following options best describes the average gender gap in Stamola? There is no difference between the average earnings of men and women Is Not Selected
Or How does your income compare to that of men in Stamola? I earn more than 90% of men Is Not Selected
Or How does your income compare to that of women in Stamola? I earn more than 90% of women Is Not Selected

EmbeddedData
FailedComprehension = 1

Block: 1M:1F 70th (DC) (12 Questions)

Branch: New Branch
If
If The video below will give you some information about the economy and your own financial situation... Is Displayed
AndIf

If Which of the following options best describes the average gender gap in Stamola? There is no difference between the average earnings of men and women Is Not Selected
Or How does your income compare to that of men in Stamola? I earn more than 70% of men Is Not Selected
Or How does your income compare to that of women in Stamola? I earn more than 70% of women Is Not Selected

EmbeddedData
FailedComprehension = 1

Standard: 1M:1F 50th (DC) (12 Questions)

Branch: New Branch
If
If The video below will give you some information about the economy and your own financial situation... Is Displayed
AndIf
If Which of the following options best describes the average gender gap in Stamola? There is no difference between the average earnings of men and women Is Not Selected
Or How does your income compare to that of men in Stamola? I earn more than 50% of men Is Not Selected
Or How does your income compare to that of women in Stamola? I earn more than 50% of women Is Not Selected

EmbeddedData
FailedComprehension = 1

Block: 1M:1F 30th (DC) (12 Questions)

Branch: New Branch
If
If The video below will give you some information about the economy and your own financial situation... Is Displayed
AndIf
If Which of the following options best describes the average gender gap in Stamola? There is no difference between the average earnings of men and women Is Not Selected
Or How does your income compare to that of men in Stamola? I earn more than 30% of men Is Not Selected
Or How does your income compare to that of women in Stamola? I earn more than 30% of women Is Not Selected

EmbeddedData
FailedComprehension = 1

Standard: 1M:1F 10th (DC) (12 Questions)

Branch: New Branch
If
If The video below will give you some information about the economy and your own financial situation... Is Displayed
AndIf
If Which of the following options best describes the average gender gap in Stamola? There is no difference between the average earnings of men and women Is Not Selected
Or How does your income compare to that of men in Stamola? I earn more than 10% of men Is Not Selected
Or How does your income compare to that of women in Stamola? I earn more than 10% of women Is Not Selected

EmbeddedData
FailedComprehension = 1

Block: 2M:3F 90th Male (DC) (12 Questions)

Branch: New Branch
If
If The video below will give you some information about the economy and your own financial situation... Is Displayed
AndIf
If Which of the following options best describes the average gender gap in Stamola? Women earn 1.5x more than men Is Not Selected
Or How does your income compare to that of men in Stamola? I earn more than 90% of men Is Not Selected
Or How does your income compare to that of women in Stamola? I earn more than 70% of women Is Not Selected

EmbeddedData
FailedComprehension = 1

Block: 2M:3F 70th Male (DC) (12 Questions)

Branch: New Branch
If
If The video below will give you some information about the economy and your own financial situation... Is Displayed
AndIf
If Which of the following options best describes the average gender gap in Stamola? Women earn 1.5x more than men Is Not Selected
Or How does your income compare to that of men in Stamola? I earn more than 70% of men Is Not Selected

Or How does your income compare to that of women in Stamola? I earn more than 50% of women Is Not Selected

EmbeddedData
FailedComprehension = 1

Block: 2M:3F 50th Male (DC) (12 Questions)

Branch: New Branch
If
If The video below will give you some information about the economy and your own financial situation... Is Displayed
AndIf
If Which of the following options best describes the average gender gap in Stamola? Women earn 1.5x more than men Is Not Selected
Or How does your income compare to that of men in Stamola? I earn more than 50% of men Is Not Selected
Or How does your income compare to that of women in Stamola? I earn more than 30% of women Is Not Selected

EmbeddedData
FailedComprehension = 1

Block: 2M:3F 30th Male (DC) (12 Questions)

Branch: New Branch
If
If The video below will give you some information about the economy and your own financial situation... Is Displayed
AndIf
If Which of the following options best describes the average gender gap in Stamola? Women earn 1.5x more than men Is Not Selected
Or How does your income compare to that of men in Stamola? I earn more than 30% of men Is Not Selected
Or How does your income compare to that of women in Stamola? I earn more than 10% of women Is Not Selected

EmbeddedData
FailedComprehension = 1

Block: 2M:3F 10th Male (DC) (12 Questions)

Branch: New Branch
If
If The video below will give you some information about the economy and your own financial situation... Is Displayed
AndIf

If Which of the following options best describes the average gender gap in Stamola? Women earn 1.5x more than men Is Not Selected
Or How does your income compare to that of men in Stamola? I earn more than 10% of men Is Not Selected
Or How does your income compare to that of women in Stamola? I earn more than <1% of women Is Not Selected

EmbeddedData
FailedComprehension = 1

Block: 1M:2F 90th Male (DC) (12 Questions)

Branch: New Branch
If
If The video below will give you some information about the economy and your own financial situation... Is Displayed
AndIf
If Which of the following options best describes the average gender gap in Stamola? Women earn 2x more than men Is Not Selected
Or How does your income compare to that of men in Stamola? I earn more than 90% of men Is Not Selected
Or How does your income compare to that of women in Stamola? I earn more than 60% of women Is Not Selected

EmbeddedData
FailedComprehension = 1

Block: 1M:2F 70th Male (DC) (12 Questions)

Branch: New Branch
If
If The video below will give you some information about the economy and your own financial situation... Is Displayed
AndIf
If Which of the following options best describes the average gender gap in Stamola? Women earn 2x more than men Is Not Selected
Or How does your income compare to that of men in Stamola? I earn more than 70% of men Is Not Selected
Or How does your income compare to that of women in Stamola? I earn more than 40% of women Is Not Selected

EmbeddedData
FailedComprehension = 1

Block: 1M:2F 50th Male (DC) (12 Questions)

Branch: New Branch
If

If The video below will give you some information about the economy and your own financial situation... Is Displayed
AndIf
If Which of the following options best describes the average gender gap in Stamola? Women earn 2x more than men Is Not Selected
Or How does your income compare to that of men in Stamola? I earn more than 50% of men Is Not Selected
Or How does your income compare to that of women in Stamola? I earn more than 20% of women Is Not Selected

EmbeddedData
FailedComprehension = 1

Block: 1M:2F 30th Male (DC) (12 Questions)

Branch: New Branch
If
If The video below will give you some information about the economy and your own financial situation... Is Displayed
AndIf
If Which of the following options best describes the average gender gap in Stamola? Women earn 2x more than men Is Not Selected
Or How does your income compare to that of men in Stamola? I earn more than 30% of men Is Not Selected
Or How does your income compare to that of women in Stamola? I earn more than 5% of women Is Not Selected

EmbeddedData
FailedComprehension = 1

Block: 1M:2F 10th Male (DC) (12 Questions)

Branch: New Branch
If
If The video below will give you some information about the economy and your own financial situation... Is Displayed
AndIf
If Which of the following options best describes the average gender gap in Stamola? Women earn 2x more than men Is Not Selected
Or How does your income compare to that of men in Stamola? I earn more than 10% of men Is Not Selected
Or How does your income compare to that of women in Stamola? I earn more than <1% of women Is Not Selected

EmbeddedData
FailedComprehension = 1

Branch: New Branch
If
If Please indicate your gender identity Woman Is Selected

BlockRandomizer: 1 - Evenly Present Elements

Standard: 2M:1F 90th Female (DC) (12 Questions)

Branch: New Branch
If
If The video below will give you some information about the economy and your own financial situation... Is Displayed
AndIf
If Which of the following options best describes the average gender gap in Stamola? Men earn 2x more than women Is Not Selected
Or How does your income compare to that of men in Stamola? I earn more than 60% of men Is Not Selected
Or How does your income compare to that of women in Stamola? I earn more than 90% of women Is Not Selected

EmbeddedData
FailedComprehension = 1

Block: 2M:1F 70th Female (DC) (12 Questions)

Branch: New Branch
If
If The video below will give you some information about the economy and your own financial situation... Is Displayed
AndIf
If Which of the following options best describes the average gender gap in Stamola? Men earn 2x more than women Is Not Selected
Or How does your income compare to that of men in Stamola? I earn more than 40% of men Is Not Selected
Or How does your income compare to that of women in Stamola? I earn more than 70% of women Is Not Selected

EmbeddedData
FailedComprehension = 1

Standard: 2M:1F 50th Female (DC) (12 Questions)

Branch: New Branch
If
If The video below will give you some information about the economy and your own financial situation... Is Displayed
AndIf

If Which of the following options best describes the average gender gap in Stamola? Men earn 2x more than women Is Not Selected
Or How does your income compare to that of men in Stamola? I earn more than 20% of men Is Not Selected
Or How does your income compare to that of women in Stamola? I earn more than 50% of women Is Not Selected

EmbeddedData
FailedComprehension = 1

Block: 2M:1F 30th Female (DC) (12 Questions)

Branch: New Branch
If
If The video below will give you some information about the economy and your own financial situation... Is Displayed
AndIf
If Which of the following options best describes the average gender gap in Stamola? Men earn 2x more than women Is Not Selected
Or How does your income compare to that of men in Stamola? I earn more than 5% of men Is Not Selected
Or How does your income compare to that of women in Stamola? I earn more than 30% of women Is Not Selected

EmbeddedData
FailedComprehension = 1

Standard: 2M:1F 10th Female (DC) (12 Questions)

Branch: New Branch
If
If The video below will give you some information about the economy and your own financial situation... Is Displayed
AndIf
If Which of the following options best describes the average gender gap in Stamola? Men earn 2x more than women Is Not Selected
Or How does your income compare to that of men in Stamola? I earn more than <1% of men Is Not Selected
Or How does your income compare to that of women in Stamola? I earn more than 10% of women Is Not Selected

EmbeddedData
FailedComprehension = 1

Standard: 3M:2F 90th Female (DC) (12 Questions)

Branch: New Branch
If

If The video below will give you some information about the economy and your own financial situation... Is Displayed
AndIf
If Which of the following options best describes the average gender gap in Stamola? Men earn 1.5x more than women Is Not Selected
Or How does your income compare to that of men in Stamola? I earn more than 70% of men Is Not Selected
Or How does your income compare to that of women in Stamola? I earn more than 90% of women Is Not Selected

EmbeddedData
FailedComprehension = 1

Block: 3M:2F 70th Female (DC) (12 Questions)

Branch: New Branch
If
If The video below will give you some information about the economy and your own financial situation... Is Displayed
AndIf
If Which of the following options best describes the average gender gap in Stamola? Men earn 1.5x more than women Is Not Selected
Or How does your income compare to that of men in Stamola? I earn more than 50% of men Is Not Selected
Or How does your income compare to that of women in Stamola? I earn more than 70% of women Is Not Selected

EmbeddedData
FailedComprehension = 1

Standard: 3M:2F 50th Female (DC) (12 Questions)

Branch: New Branch
If
If The video below will give you some information about the economy and your own financial situation... Is Displayed
AndIf
If Which of the following options best describes the average gender gap in Stamola? Men earn 1.5x more than women Is Not Selected
Or How does your income compare to that of men in Stamola? I earn more than 30% of men Is Not Selected
Or How does your income compare to that of women in Stamola? I earn more than 50% of women Is Not Selected

EmbeddedData
FailedComprehension = 1

Block: 3M:2F 30th Female (DC) (12 Questions)

Branch: New Branch
If
If The video below will give you some information about the economy and your own financial situation... Is Displayed
AndIf
If Which of the following options best describes the average gender gap in Stamola? Men earn 1.5x more than women Is Not Selected
Or How does your income compare to that of men in Stamola? I earn more than 10% of men Is Not Selected
Or How does your income compare to that of women in Stamola? I earn more than 30% of women Is Not Selected

EmbeddedData
FailedComprehension = 1

Standard: 3M:2F 10th Female (DC) (12 Questions)

Branch: New Branch
If
If The video below will give you some information about the economy and your own financial situation... Is Displayed
AndIf
If Which of the following options best describes the average gender gap in Stamola? Men earn 1.5x more than women Is Not Selected
Or How does your income compare to that of men in Stamola? I earn more than <1% of men Is Not Selected
Or How does your income compare to that of women in Stamola? I earn more than 10% of women Is Not Selected

EmbeddedData
FailedComprehension = 1

Standard: 1M:1F 90th (DC) (12 Questions)

Branch: New Branch
If
If The video below will give you some information about the economy and your own financial situation... Is Displayed
AndIf
If Which of the following options best describes the average gender gap in Stamola? There is no difference between the average earnings of men and women Is Not Selected
Or How does your income compare to that of men in Stamola? I earn more than 90% of men Is Not Selected

Or How does your income compare to that of women in Stamola? I earn more than 90% of women Is Not Selected

EmbeddedData
FailedComprehension = 1

Block: 1M:1F 70th (DC) (12 Questions)

Branch: New Branch
If
If The video below will give you some information about the economy and your own financial situation... Is Displayed
AndIf
If Which of the following options best describes the average gender gap in Stamola? There is no difference between the average earnings of men and women Is Not Selected
Or How does your income compare to that of men in Stamola? I earn more than 70% of men Is Not Selected
Or How does your income compare to that of women in Stamola? I earn more than 70% of women Is Not Selected

EmbeddedData
FailedComprehension = 1

Standard: 1M:1F 50th (DC) (12 Questions)

Branch: New Branch
If
If The video below will give you some information about the economy and your own financial situation... Is Displayed
AndIf
If Which of the following options best describes the average gender gap in Stamola? There is no difference between the average earnings of men and women Is Not Selected
Or How does your income compare to that of men in Stamola? I earn more than 50% of men Is Not Selected
Or How does your income compare to that of women in Stamola? I earn more than 50% of women Is Not Selected

EmbeddedData
FailedComprehension = 1

Block: 1M:1F 30th (DC) (12 Questions)

Branch: New Branch
If
If The video below will give you some information about the economy and your own financial situation... Is Displayed

AndIf

If Which of the following options best describes the average gender gap in Stamola? There is no difference between the average earnings of men and women Is Not Selected

Or How does your income compare to that of men in Stamola? I earn more than 30% of men Is Not Selected

Or How does your income compare to that of women in Stamola? I earn more than 30% of women Is Not Selected

EmbeddedData

FailedComprehension = 1

Standard: 1M:1F 10th (DC) (12 Questions)

Branch: New Branch

If

If The video below will give you some information about the economy and your own financial situation... Is Displayed

AndIf

If Which of the following options best describes the average gender gap in Stamola? There is no difference between the average earnings of men and women Is Not Selected

Or How does your income compare to that of men in Stamola? I earn more than 10% of men Is Not Selected

Or How does your income compare to that of women in Stamola? I earn more than 10% of women Is Not Selected

EmbeddedData

FailedComprehension = 1

Block: 2M:3F 90th Female (DC) (12 Questions)

Branch: New Branch

If

If The video below will give you some information about the economy and your own financial situation... Is Displayed

AndIf

If Which of the following options best describes the average gender gap in Stamola? Women earn 1.5x more than men Is Not Selected

Or How does your income compare to that of men in Stamola? I earn more than 99% of men Is Not Selected

Or How does your income compare to that of women in Stamola? I earn more than 90% of women Is Not Selected

EmbeddedData

FailedComprehension = 1

Block: 2M:3F 70th Female (DC) (12 Questions)

Branch: New Branch
If
If The video below will give you some information about the economy and your own financial situation... Is Displayed
AndIf
If Which of the following options best describes the average gender gap in Stamola? Women earn 1.5x more than men Is Not Selected
Or How does your income compare to that of men in Stamola? I earn more than 85% of men Is Not Selected
Or How does your income compare to that of women in Stamola? I earn more than 70% of women Is Not Selected

EmbeddedData
FailedComprehension = 1

Block: 2M:3F 50th Female (DC) (12 Questions)

Branch: New Branch
If
If The video below will give you some information about the economy and your own financial situation... Is Displayed
AndIf
If Which of the following options best describes the average gender gap in Stamola? Women earn 1.5x more than men Is Not Selected
Or How does your income compare to that of men in Stamola? I earn more than 65% of men Is Not Selected
Or How does your income compare to that of women in Stamola? I earn more than 50% of women Is Not Selected

EmbeddedData
FailedComprehension = 1

Block: 2M:3F 30th Female (DC) (12 Questions)

Branch: New Branch
If
If The video below will give you some information about the economy and your own financial situation... Is Displayed
AndIf
If Which of the following options best describes the average gender gap in Stamola? Women earn 1.5x more than men Is Not Selected
Or How does your income compare to that of men in Stamola? I earn more than 45% of men Is Not Selected

Or How does your income compare to that of women in Stamola? I earn more than 30% of women Is Not Selected

EmbeddedData
FailedComprehension = 1

Block: 2M:3F 10th Female (DC) (12 Questions)

Branch: New Branch
If
If The video below will give you some information about the economy and your own financial situation... Is Displayed
AndIf
If Which of the following options best describes the average gender gap in Stamola? Women earn 1.5x more than men Is Not Selected
Or How does your income compare to that of men in Stamola? I earn more than 25% of men Is Not Selected
Or How does your income compare to that of women in Stamola? I earn more than 10% of women Is Not Selected

EmbeddedData
FailedComprehension = 1

Block: 1M:2F 90th Female (DC) (12 Questions)

Branch: New Branch
If
If The video below will give you some information about the economy and your own financial situation... Is Displayed
AndIf
If Which of the following options best describes the average gender gap in Stamola? Women earn 2x more than men Is Not Selected
Or How does your income compare to that of men in Stamola? I earn more than 99% of men Is Not Selected
Or How does your income compare to that of women in Stamola? I earn more than 90% of women Is Not Selected

EmbeddedData
FailedComprehension = 1

Block: 1M:2F 70th Female (DC) (12 Questions)

Branch: New Branch
If
If The video below will give you some information about the economy and your own financial situation... Is Displayed
AndIf

If Which of the following options best describes the average gender gap in Stamola? Women earn 2x more than men Is Not Selected
Or How does your income compare to that of men in Stamola? I earn more than 95% of men Is Not Selected
Or How does your income compare to that of women in Stamola? I earn more than 70% of women Is Not Selected

EmbeddedData
FailedComprehension = 1

Block: 1M:2F 50th Female (DC) (12 Questions)

Branch: New Branch
If
If The video below will give you some information about the economy and your own financial situation... Is Displayed
AndIf
If Which of the following options best describes the average gender gap in Stamola? Women earn 2x more than men Is Not Selected
Or How does your income compare to that of men in Stamola? I earn more than 75% of men Is Not Selected
Or How does your income compare to that of women in Stamola? I earn more than 50% of women Is Not Selected

EmbeddedData
FailedComprehension = 1

Block: 1M:2F 30th Female (DC) (12 Questions)

Branch: New Branch
If
If The video below will give you some information about the economy and your own financial situation... Is Displayed
AndIf
If Which of the following options best describes the average gender gap in Stamola? Women earn 2x more than men Is Not Selected
Or How does your income compare to that of men in Stamola? I earn more than 55% of men Is Not Selected
Or How does your income compare to that of women in Stamola? I earn more than 30% of women Is Not Selected

EmbeddedData
FailedComprehension = 1

Block: 1M:2F 10th Female (DC) (12 Questions)

Branch: New Branch
If

If The video below will give you some information about the economy and your own financial situation... Is Displayed
AndIf
If Which of the following options best describes the average gender gap in Stamola? Women earn 2x more than men Is Not Selected
Or How does your income compare to that of men in Stamola? I earn more than 35% of men Is Not Selected
Or How does your income compare to that of women in Stamola? I earn more than 10% of women Is Not Selected

EmbeddedData
FailedComprehension = 1

Branch: New Branch
If
If Please indicate your gender identity Man Is Not Selected
And Please indicate your gender identity Woman Is Not Selected

BlockRandomizer: 1 - Evenly Present Elements

Block: 2M:1F 90th Male (DC) (12 Questions)

Branch: New Branch
If
If The video below will give you some information about the economy and your own financial situation... Is Displayed
AndIf
If Which of the following options best describes the average gender gap in Stamola? Men earn 2x more than women Is Not Selected
Or How does your income compare to that of men in Stamola? I earn more than 90% of men Is Not Selected
Or How does your income compare to that of women in Stamola? I earn more than 99% of women Is Not Selected

EmbeddedData
FailedComprehension = 1

Block: 2M:1F 70th Male (DC) (12 Questions)

Branch: New Branch
If
If The video below will give you some information about the economy and your own financial situation... Is Displayed
AndIf
If Which of the following options best describes the average gender gap in Stamola? Men earn 2x more than women Is Not Selected

Or How does your income compare to that of men in Stamola? I earn more than 70% of men Is Not Selected
Or How does your income compare to that of women in Stamola? I earn more than 95% of women Is Not Selected

EmbeddedData
FailedComprehension = 1

Block: 2M:1F 50th Male (DC) (12 Questions)

Branch: New Branch
If
If The video below will give you some information about the economy and your own financial situation... Is Displayed
AndIf
If Which of the following options best describes the average gender gap in Stamola? Men earn 2x more than women Is Not Selected
Or How does your income compare to that of men in Stamola? I earn more than 50% of men Is Not Selected
Or How does your income compare to that of women in Stamola? I earn more than 75% of women Is Not Selected

EmbeddedData
FailedComprehension = 1

Block: 2M:1F 30th Male (DC) (12 Questions)

Branch: New Branch
If
If The video below will give you some information about the economy and your own financial situation... Is Displayed
AndIf
If Which of the following options best describes the average gender gap in Stamola? Men earn 2x more than women Is Not Selected
Or How does your income compare to that of men in Stamola? I earn more than 30% of men Is Not Selected
Or How does your income compare to that of women in Stamola? I earn more than 55% of women Is Not Selected

EmbeddedData
FailedComprehension = 1

Block: 2M:1F 10th Male (DC) (12 Questions)

Branch: New Branch
If
If The video below will give you some information about the economy and your own financial situation... Is Displayed

AndIf
If Which of the following options best describes the average gender gap in Stamola? Men earn 2x more than women Is Not Selected
Or How does your income compare to that of men in Stamola? I earn more than 10% of men Is Not Selected
Or How does your income compare to that of women in Stamola? I earn more than 35% of women Is Not Selected

EmbeddedData
FailedComprehension = 1

Block: 2M:1F 90th Female (DC) (12 Questions)

Branch: New Branch
If
If The video below will give you some information about the economy and your own financial situation... Is Displayed
AndIf
If Which of the following options best describes the average gender gap in Stamola? Men earn 2x more than women Is Not Selected
Or How does your income compare to that of men in Stamola? I earn more than 60% of men Is Not Selected
Or How does your income compare to that of women in Stamola? I earn more than 90% of women Is Not Selected

EmbeddedData
FailedComprehension = 1

Block: 2M:1F 70th Female (DC) (12 Questions)

Branch: New Branch
If
If The video below will give you some information about the economy and your own financial situation... Is Displayed
AndIf
If Which of the following options best describes the average gender gap in Stamola? Men earn 2x more than women Is Not Selected
Or How does your income compare to that of men in Stamola? I earn more than 40% of men Is Not Selected
Or How does your income compare to that of women in Stamola? I earn more than 70% of women Is Not Selected

EmbeddedData
FailedComprehension = 1

Block: 2M:1F 50th Female (DC) (12 Questions)

Branch: New Branch

If

If The video below will give you some information about the economy and your own financial situation... Is Displayed

AndIf

If Which of the following options best describes the average gender gap in Stamola? Men earn 2x more than women Is Not Selected

Or How does your income compare to that of men in Stamola? I earn more than 20% of men Is Not Selected

Or How does your income compare to that of women in Stamola? I earn more than 50% of women Is Not Selected

EmbeddedData

FailedComprehension = 1

Block: 2M:1F 30th Female (DC) (12 Questions)

Branch: New Branch

If

If The video below will give you some information about the economy and your own financial situation... Is Displayed

AndIf

If Which of the following options best describes the average gender gap in Stamola? Men earn 2x more than women Is Not Selected

Or How does your income compare to that of men in Stamola? I earn more than 5% of men Is Not Selected

Or How does your income compare to that of women in Stamola? I earn more than 30% of women Is Not Selected

EmbeddedData

FailedComprehension = 1

Block: 2M:1F 10th Female (DC) (12 Questions)

Branch: New Branch

If

If The video below will give you some information about the economy and your own financial situation... Is Displayed

AndIf

If Which of the following options best describes the average gender gap in Stamola? Men earn 2x more than women Is Not Selected

Or How does your income compare to that of men in Stamola? I earn more than <1% of men Is Not Selected

Or How does your income compare to that of women in Stamola? I earn more than 10% of women Is Not Selected

EmbeddedData

FailedComprehension = 1

Block: 3M:2F 90th Male (DC) (12 Questions)

Branch: New Branch
If
If The video below will give you some information about the economy and your own financial situation... Is Displayed
AndIf
If Which of the following options best describes the average gender gap in Stamola? Men earn 1.5x more than women Is Not Selected
Or How does your income compare to that of men in Stamola? I earn more than 90% of men Is Not Selected
Or How does your income compare to that of women in Stamola? I earn more than 99% of women Is Not Selected

EmbeddedData
FailedComprehension = 1

Block: 3M:2F 70th Male (DC) (12 Questions)

Branch: New Branch
If
If The video below will give you some information about the economy and your own financial situation... Is Displayed
AndIf
If Which of the following options best describes the average gender gap in Stamola? Men earn 1.5x more than women Is Not Selected
Or How does your income compare to that of men in Stamola? I earn more than 70% of men Is Not Selected
Or How does your income compare to that of women in Stamola? I earn more than 85% of women Is Not Selected

EmbeddedData
FailedComprehension = 1

Block: 3M:2F 50th Male (DC) (12 Questions)

Branch: New Branch
If
If The video below will give you some information about the economy and your own financial situation... Is Displayed
AndIf
If Which of the following options best describes the average gender gap in Stamola? Men earn 1.5x more than women Is Not Selected
Or How does your income compare to that of men in Stamola? I earn more than 50% of men Is Not Selected

Or How does your income compare to that of women in Stamola? I earn more than 65% of women Is Not Selected

EmbeddedData
FailedComprehension = 1

Block: 3M:2F 30th Male (DC) (12 Questions)

Branch: New Branch
If
If The video below will give you some information about the economy and your own financial situation... Is Displayed
AndIf
If Which of the following options best describes the average gender gap in Stamola? Men earn 1.5x more than women Is Not Selected
Or How does your income compare to that of men in Stamola? I earn more than 30% of men Is Not Selected
Or How does your income compare to that of women in Stamola? I earn more than 45% of women Is Not Selected

EmbeddedData
FailedComprehension = 1

Block: 3M:2F 10th Male (DC) (12 Questions)

Branch: New Branch
If
If The video below will give you some information about the economy and your own financial situation... Is Displayed
AndIf
If Which of the following options best describes the average gender gap in Stamola? Men earn 1.5x more than women Is Not Selected
Or How does your income compare to that of men in Stamola? I earn more than 10% of men Is Not Selected
Or How does your income compare to that of women in Stamola? I earn more than 25% of women Is Not Selected

EmbeddedData
FailedComprehension = 1

Block: 3M:2F 90th Female (DC) (12 Questions)

Branch: New Branch
If
If The video below will give you some information about the economy and your own financial situation... Is Displayed
AndIf

If Which of the following options best describes the average gender gap in Stamola? Men earn 1.5x more than women Is Not Selected
Or How does your income compare to that of men in Stamola? I earn more than 70% of men Is Not Selected
Or How does your income compare to that of women in Stamola? I earn more than 90% of women Is Not Selected

EmbeddedData
FailedComprehension = 1

Block: 3M:2F 70th Female (DC) (12 Questions)

Branch: New Branch
If
If The video below will give you some information about the economy and your own financial situation... Is Displayed
AndIf
If Which of the following options best describes the average gender gap in Stamola? Men earn 1.5x more than women Is Not Selected
Or How does your income compare to that of men in Stamola? I earn more than 50% of men Is Not Selected
Or How does your income compare to that of women in Stamola? I earn more than 70% of women Is Not Selected

EmbeddedData
FailedComprehension = 1

Block: 3M:2F 50th Female (DC) (12 Questions)

Branch: New Branch
If
If The video below will give you some information about the economy and your own financial situation... Is Displayed
AndIf
If Which of the following options best describes the average gender gap in Stamola? Men earn 1.5x more than women Is Not Selected
Or How does your income compare to that of men in Stamola? I earn more than 30% of men Is Not Selected
Or How does your income compare to that of women in Stamola? I earn more than 50% of women Is Not Selected

EmbeddedData
FailedComprehension = 1

Block: 3M:2F 30th Female (DC) (12 Questions)

Branch: New Branch
If

If The video below will give you some information about the economy and your own financial situation... Is Displayed
AndIf
If Which of the following options best describes the average gender gap in Stamola? Men earn 1.5x more than women Is Not Selected
Or How does your income compare to that of men in Stamola? I earn more than 10% of men Is Not Selected
Or How does your income compare to that of women in Stamola? I earn more than 30% of women Is Not Selected

EmbeddedData
FailedComprehension = 1

Block: 3M:2F 10th Female (DC) (12 Questions)

Branch: New Branch
If
If The video below will give you some information about the economy and your own financial situation... Is Displayed
AndIf
If Which of the following options best describes the average gender gap in Stamola? Men earn 1.5x more than women Is Not Selected
Or How does your income compare to that of men in Stamola? I earn more than <1% of men Is Not Selected
Or How does your income compare to that of women in Stamola? I earn more than 10% of women Is Not Selected

EmbeddedData
FailedComprehension = 1

Block: 1M:1F 90th (DC) (12 Questions)

Branch: New Branch
If
If The video below will give you some information about the economy and your own financial situation... Is Displayed
AndIf
If Which of the following options best describes the average gender gap in Stamola? There is no difference between the average earnings of men and women Is Not Selected
Or How does your income compare to that of men in Stamola? I earn more than 90% of men Is Not Selected
Or How does your income compare to that of women in Stamola? I earn more than 90% of women Is Not Selected

EmbeddedData
FailedComprehension = 1

Block: 1M:1F 90th (DC) (12 Questions)

Branch: New Branch
If
If The video below will give you some information about the economy and your own financial situation... Is Displayed
AndIf
If Which of the following options best describes the average gender gap in Stamola? There is no difference between the average earnings of men and women Is Not Selected
Or How does your income compare to that of men in Stamola? I earn more than 90% of men Is Not Selected
Or How does your income compare to that of women in Stamola? I earn more than 90% of women Is Not Selected

EmbeddedData
FailedComprehension = 1

Block: 1M:1F 70th (DC) (12 Questions)

Branch: New Branch
If
If The video below will give you some information about the economy and your own financial situation... Is Displayed
AndIf
If Which of the following options best describes the average gender gap in Stamola? There is no difference between the average earnings of men and women Is Not Selected
Or How does your income compare to that of men in Stamola? I earn more than 70% of men Is Not Selected
Or How does your income compare to that of women in Stamola? I earn more than 70% of women Is Not Selected

EmbeddedData
FailedComprehension = 1

Block: 1M:1F 70th (DC) (12 Questions)

Branch: New Branch
If
If The video below will give you some information about the economy and your own financial situation... Is Displayed
AndIf
If Which of the following options best describes the average gender gap in Stamola? There is no difference between the average earnings of men and women Is Not Selected

Or How does your income compare to that of men in Stamola? I earn more than 70% of men Is Not Selected

Or How does your income compare to that of women in Stamola? I earn more than 70% of women Is Not Selected

EmbeddedData

FailedComprehension = 1

Block: 1M:1F 50th (DC) (12 Questions)

Branch: New Branch

If

If The video below will give you some information about the economy and your own financial situation... Is Displayed

AndIf

If Which of the following options best describes the average gender gap in Stamola? There is no difference between the average earnings of men and women Is Not Selected

Or How does your income compare to that of men in Stamola? I earn more than 50% of men Is Not Selected

Or How does your income compare to that of women in Stamola? I earn more than 50% of women Is Not Selected

EmbeddedData

FailedComprehension = 1

Block: 1M:1F 50th (DC) (12 Questions)

Branch: New Branch

If

If The video below will give you some information about the economy and your own financial situation... Is Displayed

AndIf

If Which of the following options best describes the average gender gap in Stamola? There is no difference between the average earnings of men and women Is Not Selected

Or How does your income compare to that of men in Stamola? I earn more than 50% of men Is Not Selected

Or How does your income compare to that of women in Stamola? I earn more than 50% of women Is Not Selected

EmbeddedData

FailedComprehension = 1

Block: 1M:1F 30th (DC) (12 Questions)

Branch: New Branch

If

If The video below will give you some information about the economy and your own financial situation... Is Displayed
AndIf
If Which of the following options best describes the average gender gap in Stamola? There is no difference between the average earnings of men and women Is Not Selected
Or How does your income compare to that of men in Stamola? I earn more than 30% of men Is Not Selected
Or How does your income compare to that of women in Stamola? I earn more than 30% of women Is Not Selected

EmbeddedData
FailedComprehension = 1

Block: 1M:1F 30th (DC) (12 Questions)

Branch: New Branch
If
If The video below will give you some information about the economy and your own financial situation... Is Displayed
AndIf
If Which of the following options best describes the average gender gap in Stamola? There is no difference between the average earnings of men and women Is Not Selected
Or How does your income compare to that of men in Stamola? I earn more than 30% of men Is Not Selected
Or How does your income compare to that of women in Stamola? I earn more than 30% of women Is Not Selected

EmbeddedData
FailedComprehension = 1

Block: 1M:1F 10th (DC) (12 Questions)

Branch: New Branch
If
If The video below will give you some information about the economy and your own financial situation... Is Displayed
AndIf
If Which of the following options best describes the average gender gap in Stamola? There is no difference between the average earnings of men and women Is Not Selected
Or How does your income compare to that of men in Stamola? I earn more than 10% of men Is Not Selected
Or How does your income compare to that of women in Stamola? I earn more than 10% of women Is Not Selected

EmbeddedData
FailedComprehension = 1

Block: 1M:1F 10th (DC) (12 Questions)

Branch: New Branch
If
If The video below will give you some information about the economy and your own financial situation... Is Displayed
AndIf
If Which of the following options best describes the average gender gap in Stamola? There is no difference between the average earnings of men and women Is Not Selected
Or How does your income compare to that of men in Stamola? I earn more than 10% of men Is Not Selected
Or How does your income compare to that of women in Stamola? I earn more than 10% of women Is Not Selected

EmbeddedData
FailedComprehension = 1

Block: 2M:3F 90th Male (DC) (12 Questions)

Branch: New Branch
If
If The video below will give you some information about the economy and your own financial situation... Is Displayed
AndIf
If Which of the following options best describes the average gender gap in Stamola? Women earn 1.5x more than men Is Not Selected
Or How does your income compare to that of men in Stamola? I earn more than 90% of men Is Not Selected
Or How does your income compare to that of women in Stamola? I earn more than 70% of women Is Not Selected

EmbeddedData
FailedComprehension = 1

Block: 2M:3F 70th Male (DC) (12 Questions)

Branch: New Branch
If
If The video below will give you some information about the economy and your own financial situation... Is Displayed
AndIf
If Which of the following options best describes the average gender gap in Stamola? Women earn 1.5x more than men Is Not Selected

Or How does your income compare to that of men in Stamola? I earn more than 70% of men Is Not Selected
Or How does your income compare to that of women in Stamola? I earn more than 50% of women Is Not Selected

EmbeddedData
FailedComprehension = 1

Block: 2M:3F 50th Male (DC) (12 Questions)

Branch: New Branch
If
If The video below will give you some information about the economy and your own financial situation... Is Displayed
AndIf
If Which of the following options best describes the average gender gap in Stamola? Women earn 1.5x more than men Is Not Selected
Or How does your income compare to that of men in Stamola? I earn more than 50% of men Is Not Selected
Or How does your income compare to that of women in Stamola? I earn more than 30% of women Is Not Selected

EmbeddedData
FailedComprehension = 1

Block: 2M:3F 30th Male (DC) (12 Questions)

Branch: New Branch
If
If The video below will give you some information about the economy and your own financial situation... Is Displayed
AndIf
If Which of the following options best describes the average gender gap in Stamola? Women earn 1.5x more than men Is Not Selected
Or How does your income compare to that of men in Stamola? I earn more than 30% of men Is Not Selected
Or How does your income compare to that of women in Stamola? I earn more than 10% of women Is Not Selected

EmbeddedData
FailedComprehension = 1

Block: 2M:3F 10th Male (DC) (12 Questions)

Branch: New Branch
If
If The video below will give you some information about the economy and your own financial situation... Is Displayed

AndIf

If Which of the following options best describes the average gender gap in Stamola? Women earn 1.5x more than men Is Not Selected

Or How does your income compare to that of men in Stamola? I earn more than 10% of men Is Not Selected

Or How does your income compare to that of women in Stamola? I earn more than <1% of women Is Not Selected

EmbeddedData

FailedComprehension = 1

Block: 2M:3F 90th Female (DC) (12 Questions)

Branch: New Branch

If

If The video below will give you some information about the economy and your own financial situation... Is Displayed

AndIf

If Which of the following options best describes the average gender gap in Stamola? Women earn 1.5x more than men Is Not Selected

Or How does your income compare to that of men in Stamola? I earn more than 99% of men Is Not Selected

Or How does your income compare to that of women in Stamola? I earn more than 90% of women Is Not Selected

EmbeddedData

FailedComprehension = 1

Block: 2M:3F 70th Female (DC) (12 Questions)

Branch: New Branch

If

If The video below will give you some information about the economy and your own financial situation... Is Displayed

AndIf

If Which of the following options best describes the average gender gap in Stamola? Women earn 1.5x more than men Is Not Selected

Or How does your income compare to that of men in Stamola? I earn more than 85% of men Is Not Selected

Or How does your income compare to that of women in Stamola? I earn more than 70% of women Is Not Selected

EmbeddedData

FailedComprehension = 1

Block: 2M:3F 50th Female (DC) (12 Questions)

Branch: New Branch

If

If The video below will give you some information about the economy and your own financial situation... Is Displayed

AndIf

If Which of the following options best describes the average gender gap in Stamola? Women earn 1.5x more than men Is Not Selected

Or How does your income compare to that of men in Stamola? I earn more than 65% of men Is Not Selected

Or How does your income compare to that of women in Stamola? I earn more than 50% of women Is Not Selected

EmbeddedData

FailedComprehension = 1

Block: 2M:3F 30th Female (DC) (12 Questions)

Branch: New Branch

If

If The video below will give you some information about the economy and your own financial situation... Is Displayed

AndIf

If Which of the following options best describes the average gender gap in Stamola? Women earn 1.5x more than men Is Not Selected

Or How does your income compare to that of men in Stamola? I earn more than 45% of men Is Not Selected

Or How does your income compare to that of women in Stamola? I earn more than 30% of women Is Not Selected

EmbeddedData

FailedComprehension = 1

Block: 2M:3F 10th Female (DC) (12 Questions)

Branch: New Branch

If

If The video below will give you some information about the economy and your own financial situation... Is Displayed

AndIf

If Which of the following options best describes the average gender gap in Stamola? Women earn 1.5x more than men Is Not Selected

Or How does your income compare to that of men in Stamola? I earn more than 25% of men Is Not Selected

Or How does your income compare to that of women in Stamola? I earn more than 10% of women Is Not Selected

EmbeddedData

FailedComprehension = 1

Block: 1M:2F 90th Male (DC) (12 Questions)

Branch: New Branch
If
If The video below will give you some information about the economy and your own financial situation... Is Displayed
AndIf
If Which of the following options best describes the average gender gap in Stamola? Women earn 2x more than men Is Not Selected
Or How does your income compare to that of men in Stamola? I earn more than 90% of men Is Not Selected
Or How does your income compare to that of women in Stamola? I earn more than 60% of women Is Not Selected

EmbeddedData
FailedComprehension = 1

Block: 1M:2F 70th Male (DC) (12 Questions)

Branch: New Branch
If
If The video below will give you some information about the economy and your own financial situation... Is Displayed
AndIf
If Which of the following options best describes the average gender gap in Stamola? Women earn 2x more than men Is Not Selected
Or How does your income compare to that of men in Stamola? I earn more than 70% of men Is Not Selected
Or How does your income compare to that of women in Stamola? I earn more than 40% of women Is Not Selected

EmbeddedData
FailedComprehension = 1

Block: 1M:2F 50th Male (DC) (12 Questions)

Branch: New Branch
If
If The video below will give you some information about the economy and your own financial situation... Is Displayed
AndIf
If Which of the following options best describes the average gender gap in Stamola? Women earn 2x more than men Is Not Selected
Or How does your income compare to that of men in Stamola? I earn more than 50% of men Is Not Selected

Or How does your income compare to that of women in Stamola? I earn more than 20% of women Is Not Selected

EmbeddedData
FailedComprehension = 1

Block: 1M:2F 30th Male (DC) (12 Questions)

Branch: New Branch
If
If The video below will give you some information about the economy and your own financial situation... Is Displayed
AndIf
If Which of the following options best describes the average gender gap in Stamola? Women earn 2x more than men Is Not Selected
Or How does your income compare to that of men in Stamola? I earn more than 30% of men Is Not Selected
Or How does your income compare to that of women in Stamola? I earn more than 5% of women Is Not Selected

EmbeddedData
FailedComprehension = 1

Block: 1M:2F 10th Male (DC) (12 Questions)

Branch: New Branch
If
If The video below will give you some information about the economy and your own financial situation... Is Displayed
AndIf
If Which of the following options best describes the average gender gap in Stamola? Women earn 2x more than men Is Not Selected
Or How does your income compare to that of men in Stamola? I earn more than 10% of men Is Not Selected
Or How does your income compare to that of women in Stamola? I earn more than <1% of women Is Not Selected

EmbeddedData
FailedComprehension = 1

Block: 1M:2F 90th Female (DC) (12 Questions)

Branch: New Branch
If
If The video below will give you some information about the economy and your own financial situation... Is Displayed
AndIf

If Which of the following options best describes the average gender gap in Stamola? Women earn 2x more than men Is Not Selected
Or How does your income compare to that of men in Stamola? I earn more than 99% of men Is Not Selected
Or How does your income compare to that of women in Stamola? I earn more than 90% of women Is Not Selected

EmbeddedData
FailedComprehension = 1

Block: 1M:2F 70th Female (DC) (12 Questions)

Branch: New Branch
If
If The video below will give you some information about the economy and your own financial situation... Is Displayed
AndIf
If Which of the following options best describes the average gender gap in Stamola? Women earn 2x more than men Is Not Selected
Or How does your income compare to that of men in Stamola? I earn more than 95% of men Is Not Selected
Or How does your income compare to that of women in Stamola? I earn more than 70% of women Is Not Selected

EmbeddedData
FailedComprehension = 1

Block: 1M:2F 50th Female (DC) (12 Questions)

Branch: New Branch
If
If The video below will give you some information about the economy and your own financial situation... Is Displayed
AndIf
If Which of the following options best describes the average gender gap in Stamola? Women earn 2x more than men Is Not Selected
Or How does your income compare to that of men in Stamola? I earn more than 75% of men Is Not Selected
Or How does your income compare to that of women in Stamola? I earn more than 50% of women Is Not Selected

EmbeddedData
FailedComprehension = 1

Block: 1M:2F 30th Female (DC) (12 Questions)

Branch: New Branch
If

If The video below will give you some information about the economy and your own financial situation... Is Displayed
AndIf
If Which of the following options best describes the average gender gap in Stamola? Women earn 2x more than men Is Not Selected
Or How does your income compare to that of men in Stamola? I earn more than 55% of men Is Not Selected
Or How does your income compare to that of women in Stamola? I earn more than 30% of women Is Not Selected

EmbeddedData
FailedComprehension = 1

Block: 1M:2F 10th Female (DC) (12 Questions)

Branch: New Branch
If
If The video below will give you some information about the economy and your own financial situation... Is Displayed
AndIf
If Which of the following options best describes the average gender gap in Stamola? Women earn 2x more than men Is Not Selected
Or How does your income compare to that of men in Stamola? I earn more than 35% of men Is Not Selected
Or How does your income compare to that of women in Stamola? I earn more than 10% of women Is Not Selected

EmbeddedData
FailedComprehension = 1

Standard: Manipulation Strengthener (1 Question)
Standard: Mate Preferences 1 (3 Questions)

Branch: New Branch
If
If The video below will give you some information about the economy and your own financial situation... Is Displayed

Block: 2M:1F 90th Male - Manipulation Reminder (3 Questions)

Branch: New Branch
If
If The video below will give you some information about the economy and your own financial situation... Is Displayed

Standard: 2M:1F 90th Female - Manipulation Reminder (3 Questions)

Branch: New Branch

If

If The video below will give you some information about the economy and your own financial situation... Is Displayed

Standard: 2M:1F 70th Male - Manipulation Reminder (3 Questions)

Branch: New Branch

If

If The video below will give you some information about the economy and your own financial situation... Is Displayed

Standard: 2M:1F 70th Female - Manipulation Reminder (3 Questions)

Branch: New Branch

If

If The video below will give you some information about the economy and your own financial situation... Is Displayed

Standard: 2M:1F 50th Male - Manipulation Reminder (3 Questions)

Branch: New Branch

If

If The video below will give you some information about the economy and your own financial situation... Is Displayed

Standard: 2M:1F 50th Female - Manipulation Reminder (3 Questions)

Branch: New Branch

If

If The video below will give you some information about the economy and your own financial situation... Is Displayed

Standard: 2M:1F 30th Male - Manipulation Reminder (3 Questions)

Branch: New Branch

If

If The video below will give you some information about the economy and your own financial situation... Is Displayed

Standard: 2M:1F 30th Female - Manipulation Reminder (3 Questions)

Branch: New Branch

If

If The video below will give you some information about the economy and your own financial situation... Is Displayed

Standard: 2M:1F 10th Male - Manipulation Reminder (3 Questions)

Branch: New Branch

If

If The video below will give you some information about the economy and your own financial situation... Is Displayed

Standard: 2M:1F 10th Female - Manipulation Reminder (3 Questions)

Branch: New Branch
If
If The video below will give you some information about the economy and your own financial situation... Is Displayed

Standard: 3M:2F 90th Male - Manipulation Reminder (3 Questions)

Branch: New Branch
If
If The video below will give you some information about the economy and your own financial situation... Is Displayed

Standard: 3M:2F 90th Female - Manipulation Reminder (3 Questions)

Branch: New Branch
If
If The video below will give you some information about the economy and your own financial situation... Is Displayed

Standard: 3M:2F 70th Male - Manipulation Reminder (3 Questions)

Branch: New Branch
If
If The video below will give you some information about the economy and your own financial situation... Is Displayed

Standard: 3M:2F 70th Female - Manipulation Reminder (3 Questions)

Branch: New Branch
If
If The video below will give you some information about the economy and your own financial situation... Is Displayed

Standard: 3M:2F 50th Male - Manipulation Reminder (3 Questions)

Branch: New Branch
If
If The video below will give you some information about the economy and your own financial situation... Is Displayed

Standard: 3M:2F 50th Female - Manipulation Reminder (3 Questions)

Branch: New Branch
If

If The video below will give you some information about the economy and your own financial situation... Is Displayed

Standard: 3M:2F 30th Male - Manipulation Reminder (3 Questions)

Branch: New Branch
If
If The video below will give you some information about the economy and your own financial situation... Is Displayed

Standard: 3M:2F 30th Female - Manipulation Reminder (3 Questions)

Branch: New Branch
If
If The video below will give you some information about the economy and your own financial situation... Is Displayed

Standard: 3M:2F 10th Male - Manipulation Reminder (3 Questions)

Branch: New Branch
If
If The video below will give you some information about the economy and your own financial situation... Is Displayed

Standard: 3M:2F 10th Female - Manipulation Reminder (3 Questions)

Branch: New Branch
If
If The video below will give you some information about the economy and your own financial situation... Is Displayed

Standard: 1M:1F 90th - Manipulation Reminder (3 Questions)

Branch: New Branch
If
If The video below will give you some information about the economy and your own financial situation... Is Displayed

Standard: 1M:1F 70th - Manipulation Reminder (3 Questions)

Branch: New Branch
If
If The video below will give you some information about the economy and your own financial situation... Is Displayed

Standard: 1M:1F 50th - Manipulation Reminder (3 Questions)

Branch: New Branch
If

If The video below will give you some information about the economy and your own financial situation... Is Displayed

Standard: 1M:1F 30th - Manipulation Reminder (3 Questions)

Branch: New Branch
If
If The video below will give you some information about the economy and your own financial situation... Is Displayed

Standard: 1M:1F 10th - Manipulation Reminder (3 Questions)

Branch: New Branch
If
If The video below will give you some information about the economy and your own financial situation... Is Displayed

Standard: 2M:3F 90th Male - Manipulation Reminder (3 Questions)

Branch: New Branch
If
If The video below will give you some information about the economy and your own financial situation... Is Displayed

Standard: 2M:3F 90th Female - Manipulation Reminder (3 Questions)

Branch: New Branch
If
If The video below will give you some information about the economy and your own financial situation... Is Displayed

Standard: 2M:3F 70th Male - Manipulation Reminder (3 Questions)

Branch: New Branch
If
If The video below will give you some information about the economy and your own financial situation... Is Displayed

Standard: 2M:3F 70th Female - Manipulation Reminder (3 Questions)

Branch: New Branch
If
If The video below will give you some information about the economy and your own financial situation... Is Displayed

Standard: 2M:3F 50th Male - Manipulation Reminder (3 Questions)

Branch: New Branch
If

If The video below will give you some information about the economy and your own financial situation... Is Displayed

Standard: 2M:3F 50th Female - Manipulation Reminder (3 Questions)

Branch: New Branch

If

If The video below will give you some information about the economy and your own financial situation... Is Displayed

Standard: 2M:3F 30th Male - Manipulation Reminder (3 Questions)

Branch: New Branch

If

If The video below will give you some information about the economy and your own financial situation... Is Displayed

Standard: 2M:3F 30th Female - Manipulation Reminder (3 Questions)

Branch: New Branch

If

If The video below will give you some information about the economy and your own financial situation... Is Displayed

Standard: 2M:3F 10th Male - Manipulation Reminder (3 Questions)

Branch: New Branch

If

If The video below will give you some information about the economy and your own financial situation... Is Displayed

Standard: 2M:3F 10th Female - Manipulation Reminder (3 Questions)

Branch: New Branch

If

If The video below will give you some information about the economy and your own financial situation... Is Displayed

Standard: 1M:2F 90th Male - Manipulation Reminder (3 Questions)

Branch: New Branch

If

If The video below will give you some information about the economy and your own financial situation... Is Displayed

Standard: 1M:2F 90th Female - Manipulation Reminder (3 Questions)

Branch: New Branch

If

If The video below will give you some information about the economy and your own financial situation... Is Displayed

Standard: 1M:2F 70th Male - Manipulation Reminder (3 Questions)

Branch: New Branch
If
If The video below will give you some information about the economy and your own financial situation... Is Displayed

Block: 1M:2F 70th Female - Manipulation Reminder (3 Questions)

Branch: New Branch
If
If The video below will give you some information about the economy and your own financial situation... Is Displayed

Standard: 1M:2F 50th Male - Manipulation Reminder (3 Questions)

Branch: New Branch
If
If The video below will give you some information about the economy and your own financial situation... Is Displayed

Standard: 1M:2F 50th Female - Manipulation Reminder (3 Questions)

Branch: New Branch
If
If The video below will give you some information about the economy and your own financial situation... Is Displayed

Standard: 1M:2F 30th Male - Manipulation Reminder (3 Questions)

Branch: New Branch
If
If The video below will give you some information about the economy and your own financial situation... Is Displayed

Standard: 1M:2F 30th Female - Manipulation Reminder (3 Questions)

Branch: New Branch
If
If The video below will give you some information about the economy and your own financial situation... Is Displayed

Standard: 1M:2F 10th Male - Manipulation Reminder (3 Questions)

Branch: New Branch
If

If The video below will give you some information about the economy and your own financial situation... Is Displayed

Standard: 1M:2F 10th Female - Manipulation Reminder (3 Questions)

Standard: Mate Preferences 2 (2 Questions)
Standard: Mate-seeking Questionnaire (1 Question)
Standard: Debrief (1 Question)

EndSurvey: Advanced

Page Break

Start of Block: Pre-screening

prolific_id **Please enter your Prolific ID** *Please note that this response should auto-fill with the correct ID*

____

Page Break

age What is your age in years?

▼ 18 (18) ... 100 (100)

Page Break

english Are you fluent in English?

- Yes (1)
- No (2)

Page Break

captcha Complete the CAPTCHA below.

End of Block: Pre-screening

Start of Block: Consent

REDACTED FOR PEER REVIEW

- ◯ I agree, start questionnaire (1)
- ◯ I do not agree (2)

End of Block: Consent

Start of Block: Demographics

sexuality How would you describe your sexuality?

- ◯ Exclusively attracted to men (1)
- ◯ Predominantly attracted to men, only incidentally attracted to women (2)
- ◯ Predominantly attracted to men, but more than incidentally attracted to women (3)
- ◯ Equally attracted to men and women (4)
- ◯ Predominantly attracted to women, but more than incidentally attracted to men (5)
- ◯ Predominantly attracted to women, only incidentally attracted to men (6)
- ◯ Exclusively attracted to women (7)
- ◯ Asexual (No socio-sexual contacts or reactions) (8)
- ◯ Pansexual (Attracted toward people regardless of sex or gender identity) (9)
- ◯ Other (Please specify) (10) ____

Page Break

gender Please indicate your gender identity

○ Man (1)

○ Woman (2)

○ Non-binary (3)

○ I use a different term (Please enter term below) (4)
____

○ Prefer not to say (5)

Page Break

X→

sex Please indicate your biological sex at birth

- ○ Male (1)
- ○ Female (2)
- ○ Intersex (3)
- ○ Other (Please specify) (4) ____
- ○ Prefer not to say (5)

End of Block: Demographics

Start of Block: Pre-Stamola Wake-up Call

Pre In this study, we will need you to pay attention to some important information. In order for you to do so, it is important that you are paying full attention to the study, and not listening to music, or driving a car, or doing anything else that takes up your attention. Please make sure you can focus just on this study while you complete it. Thank you.

End of Block: Pre-Stamola Wake-up Call

Start of Block: Stamola Intro

welcome Stamola is a new virtual society and in this study, you will become a citizen of Stamola. You will start a new life there and become a member of Stamolean society.

End of Block: Stamola Intro

Start of Block: 2M:1F 90th Male (DC)

timer Timing
First Click (1)
Last Click (2)
Page Submit (3)
Click Count (4)

2M1F90MInstruct The video below will give you some information about the economy and your own financial situation in Stamola. Please watch the video carefully, because attention checks are included on the following page. These attention checks will be used to confirm whether you've understood, and remembered, key details about the economy in Stamola and your own financial situation. A button will appear in the lower right hand corner of the screen once the video is finished, and you will be able to proceed to the next part of the survey.

2M1F90MVideo

Page Break

ratio2M1F90M Which of the following options best describes **the average gender gap** in Stamola**?**

- ○ Men earn 2x more than women (1)
- ○ Men earn 1.5x more than women (2)
- ○ There is no difference between the average earnings of men and women (3)
- ○ Women earn 1.5x more than men (4)
- ○ Women earn 2x more than men (5)

---

wpercent2M1F90M How does **your** income compare to that of **women** in Stamola?

- ○ I earn more than 35% of women (1)
- ○ I earn more than 55% of women (2)
- ○ I earn more than 75% of women (3)
- ○ I earn more than 95% of women (4)
- ○ I earn more than 99% of women (5)

---

mpercent2M1F90M How does **your** income compare to that of **men** in Stamola?

- I earn more than 10% of men (1)
- I earn more than 30% of men (2)
- I earn more than 50% of men (3)
- I earn more than 70% of men (4)
- I earn more than 90% of men (5)

Page Break

gap2M1F90M How **gender unequal** is income in Stamola?

- 1 Not at all gender unequal (1)
- 2 (2)
- 3 (3)
- 4 Moderately gender unequal (4)
- 5 (5)
- 6 (6)
- 7 Extremely gender unequal (7)

---

wwealth2M1F90M Compared to **women** in Stamola, how **wealthy** are **you**?

- 1 Extremely poor (1)
- 2 (2)
- 3 (3)
- 4 Neither poor nor wealthy (4)
- 5 (5)
- 6 (8)
- 7 Extremely wealthy (9)

---

mwealth2M1F90M Compared **men** in Stamola, how **wealthy** are **you**?

- 1 Extremely poor (1)
- 2 (2)
- 3 (3)
- 4 Neither poor nor wealthy (4)
- 5 (5)
- 6 (8)
- 7 Extremely wealthy (9)

Page Break

2M1F90Mstr

Q1080 **Think about your financial situation in Stamola.** Given how much money ${e://Field/opgenpeople} make, and how much money you make relative to others, **what would your romantic relationships with ${e://Field/opgenpeople} be like in Stamolean society?** Specifically, what would you want in a partner? **Reflect on these questions for 30 seconds, and record your thoughts using the text box below.** As you progress throughout the survey, answer any questions with these thoughts in mind. After 30 seconds, a button will appear in the lower right hand corner of the screen and you may proceed to the next part of the survey.

____

____

____

____

____

Q1081 Timing
First Click (1)
Last Click (2)
Page Submit (3)
Click Count (4)

End of Block: 2M:1F 90th Male (DC)

Start of Block: 2M:1F 70th Male (DC)

Q2081 Timing
First Click (1)
Last Click (2)
Page Submit (3)
Click Count (4)

2M1F70Minstruct The video below will give you some information about the economy and your own financial situation in Stamola. Please watch the video carefully, because attention checks

are included on the following page. These attention checks will be used to confirm whether you've understood, and remembered, key details about the economy in Stamola and your own financial situation. A button will appear in the lower right hand corner of the screen once the video is finished, and you will be able to proceed to the next part of the survey.

2M1F70Mvideo

Page Break

ratio2M1F70M Which of the following options best describes **the average gender gap** in Stamola**?**

- Men earn 2x more than women (1)
- Men earn 1.5x more than women (2)
- There is no difference between the average earnings of men and women (3)
- Women earn 1.5x more than men (4)
- Women earn 2x more than men (5)

---

wpercent2M1F70M How does **your** income compare to that of **women** in Stamola?

- I earn more than 35% of women (1)
- I earn more than 55% of women (2)
- I earn more than 75% of women (3)
- I earn more than 95% of women (4)
- I earn more than 99% of women (5)

---

mpercent2M1F70M How does **your** income compare to that of **men** in Stamola?

- ○ I earn more than 10% of men (1)
- ○ I earn more than 30% of men (2)
- ○ I earn more than 50% of men (3)
- ○ I earn more than 70% of men (4)
- ○ I earn more than 90% of men (5)

Page Break

gap2M1F70M How **gender unequal** is income in Stamola?

- 1 Not at all gender unequal (1)
- 2 (2)
- 3 (3)
- 4 Moderately gender unequal (4)
- 5 (5)
- 6 (6)
- 7 Extremely gender unequal (7)

---

wwealth2M1F70M Compared to **women** in Stamola, how **wealthy** are **you**?

- 1 Extremely poor (1)
- 2 (2)
- 3 (3)
- 4 Neither poor nor wealthy (4)
- 5 (5)
- 6 (8)
- 7 Extremely wealthy (9)

---

mwealth2M1F70M Compared to **men** in Stamola, how **wealthy** are **you**?

- 1 Extremely poor (1)
- 2 (2)
- 3 (3)
- 4 Neither poor nor wealthy (4)
- 5 (5)
- 6 (8)
- 7 Extremely wealthy (9)

Page Break

Q1129

Q1128 **Think about your financial situation in Stamola.** Given how much money ${e://Field/opgenpeople} make, and how much money you make relative to others, **what would your romantic relationships with ${e://Field/opgenpeople} be like in Stamolean society?** Specifically, what would you want in a partner? **Reflect on these questions for 30 seconds, and record your thoughts using the text box below.** As you progress throughout the survey, answer any questions with these thoughts in mind. After 30 seconds, a button will appear in the lower right hand corner of the screen and you may proceed to the next part of the survey.

____

____

____

____

____

Q1174 Timing
First Click (1)
Last Click (2)
Page Submit (3)
Click Count (4)

End of Block: 2M:1F 70th Male (DC)

Start of Block: 2M:1F 50th Male (DC)

Q2090 Timing
First Click (1)
Last Click (2)
Page Submit (3)
Click Count (4)

instruct2M1F50M The video below will give you some information about the economy and your own financial situation in Stamola. Please watch the video carefully, because attention checks

are included on the following page. These attention checks will be used to confirm whether you've understood, and remembered, key details about the economy in Stamola and your own financial situation. A button will appear in the lower right hand corner of the screen once the video is finished, and you will be able to proceed to the next part of the survey.

---

video2M1F50M

Page Break

ratio2M1F50M Which of the following options best describes **the average gender gap** in Stamola?

- Men earn 2x more than women (1)
- Men earn 1.5x more than women (2)
- There is no difference between the average earnings of men and women (3)
- Women earn 1.5x more than men (4)
- Women earn 2x more than men (5)

---

wpercent2M1F50M How does **your** income compare to that of **women** in Stamola?

- I earn more than 35% of women (1)
- I earn more than 55% of women (2)
- I earn more than 75% of women (3)
- I earn more than 95% of women (4)
- I earn more than 99% of women (5)

---

mpercent2M1F50M How does **your** income compare to that of **men** in Stamola?

- ○ I earn more than 10% of men (1)
- ○ I earn more than 30% of men (2)
- ○ I earn more than 50% of men (3)
- ○ I earn more than 70% of men (4)
- ○ I earn more than 90% of men (5)

Page Break

gap2M1F50M How **gender unequal** is income in Stamola?

- 1 Not at all gender unequal (1)
- 2 (2)
- 3 (3)
- 4 Moderately gender unequal (4)
- 5 (5)
- 6 (6)
- 7 Extremely gender unequal (7)

---

wwealth2M1F50M Compared to **women** in Stamola, how **wealthy** are **you**?

- 1 Extremely poor (1)
- 2 (2)
- 3 (3)
- 4 Neither poor nor wealthy (4)
- 5 (5)
- 6 (8)
- 7 Extremely wealthy (9)

---

mwealth2M1F50M Compared to **men** in Stamola, how **wealthy** are **you**?

- 1 Extremely poor (1)
- 2 (2)
- 3 (3)
- 4 Neither poor nor wealthy (4)
- 5 (5)
- 6 (8)
- 7 Extremely wealthy (9)

Page Break

Q1086

Q1130 **Think about your financial situation in Stamola.** Given how much money ${e://Field/opgenpeople} make, and how much money you make relative to others, **what would your romantic relationships with ${e://Field/opgenpeople} be like in Stamolean society?** Specifically, what would you want in a partner? **Reflect on these questions for 30 seconds, and record your thoughts using the text box below.** As you progress throughout the survey, answer any questions with these thoughts in mind. After 30 seconds, a button will appear in the lower right hand corner of the screen and you may proceed to the next part of the survey.

____

____

____

____

____

Q1175 Timing
First Click (1)
Last Click (2)
Page Submit (3)
Click Count (4)

End of Block: 2M:1F 50th Male (DC)

Start of Block: 2M:1F 30th Male (DC)

Q2099 Timing
First Click (1)
Last Click (2)
Page Submit (3)
Click Count (4)

instruct2M1F30M The video below will give you some information about the economy and your own financial situation in Stamola. Please watch the video carefully, because attention checks

are included on the following page. These attention checks will be used to confirm whether you've understood, and remembered, key details about the economy in Stamola and your own financial situation. A button will appear in the lower right hand corner of the screen once the video is finished, and you will be able to proceed to the next part of the survey.

video2M1F30M

Page Break

ratio2M1F30M Which of the following options best describes **the average gender gap** in Stamola**?**

- Men earn 2x more than women (1)
- Men earn 1.5x more than women (2)
- There is no difference between the average earnings of men and women (3)
- Women earn 1.5x more than men (4)
- Women earn 2x more than men (5)

---

wpercent2M1F30M How does **your** income compare to that of **women** in Stamola?

- I earn more than 35% of women (1)
- I earn more than 55% of women (2)
- I earn more than 75% of women (3)
- I earn more than 95% of women (4)
- I earn more than 99% of women (5)

---

mpercent2M1F30M How does **your** income compare to that of **men** in Stamola?

- I earn more than 10% of men (1)
- I earn more than 30% of men (2)
- I earn more than 50% of men (3)
- I earn more than 70% of men (4)
- I earn more than 90% of men (5)

Page Break

gap2M1F30M How **gender unequal** is income in Stamola?

- 1 Not at all gender unequal (1)
- 2 (2)
- 3 (3)
- 4 Moderately gender unequal (4)
- 5 (5)
- 6 (6)
- 7 Extremely gender unequal (7)

---

wwealth2M1F30M Compared to **women** in Stamola, how **wealthy** are **you**?

- 1 Extremely poor (1)
- 2 (2)
- 3 (3)
- 4 Neither poor nor wealthy (4)
- 5 (5)
- 6 (8)
- 7 Extremely wealthy (9)

---

mwealth2M1F30M Compared to **men** in Stamola, how **wealthy** are **you**?

- 1 Extremely poor (1)
- 2 (2)
- 3 (3)
- 4 Neither poor nor wealthy (4)
- 5 (5)
- 6 (8)
- 7 Extremely wealthy (9)

Page Break

Q1088

Q1131 **Think about your financial situation in Stamola.** Given how much money ${e://Field/opgenpeople} make, and how much money you make relative to others, **what would your romantic relationships with ${e://Field/opgenpeople} be like in Stamolean society?** Specifically, what would you want in a partner? **Reflect on these questions for 30 seconds, and record your thoughts using the text box below.** As you progress throughout the survey, answer any questions with these thoughts in mind. After 30 seconds, a button will appear in the lower right hand corner of the screen and you may proceed to the next part of the survey.

____

____

____

____

____

Q1176 Timing
First Click (1)
Last Click (2)
Page Submit (3)
Click Count (4)

End of Block: 2M:1F 30th Male (DC)

Start of Block: 2M:1F 10th Male (DC)

Q2108 Timing
First Click (1)
Last Click (2)
Page Submit (3)
Click Count (4)

instruct2M1F10M The video below will give you some information about the economy and your own financial situation in Stamola. Please watch the video carefully, because attention checks

are included on the following page. These attention checks will be used to confirm whether you've understood, and remembered, key details about the economy in Stamola and your own financial situation. A button will appear in the lower right hand corner of the screen once the video is finished, and you will be able to proceed to the next part of the survey.

video2M1F10M

Page Break

ratio2M1F10M Which of the following options best describes **the average gender gap** in Stamola**?**

- Men earn 2x more than women (1)
- Men earn 1.5x more than women (2)
- There is no difference between the average earnings of men and women (3)
- Women earn 1.5x more than men (4)
- Women earn 2x more than men (5)

---

wpercent2M1F10M How does **your** income compare to that of **women** in Stamola?

- I earn more than 35% of women (1)
- I earn more than 55% of women (2)
- I earn more than 75% of women (3)
- I earn more than 95% of women (4)
- I earn more than 99% of women (5)

---

mpercent2M1F10M How does **your** income compare to that of **men** in Stamola?

- I earn more than 10% of men (1)
- I earn more than 30% of men (2)
- I earn more than 50% of men (3)
- I earn more than 70% of men (4)
- I earn more than 90% of men (5)

Page Break

gap2M1F10M How **gender unequal** is income in Stamola?

○ 1 Not at all gender unequal (1)

○ 2 (2)

○ 3 (3)

○ 4 Moderately gender unequal (4)

○ 5 (5)

○ 6 (6)

○ 7 Extremely gender unequal (7)

---

wwealth2M1F10M Compared to **women** in Stamola, how **wealthy** are **you**?

○ 1 Extremely poor (1)

○ 2 (2)

○ 3 (3)

○ 4 Neither poor nor wealthy (4)

○ 5 (5)

○ 5 (8)

○ 7 Extremely wealthy (9)

---

mwealth2M1F10M Compared to **men** in Stamola, how **wealthy** are **you**?

- 1 Extremely poor (1)
- 2 (2)
- 3 (3)
- 4 Neither poor nor wealthy (4)
- 5 (5)
- 6 (8)
- 7 Extremely wealthy (9)

Page Break

Q1090

Q1132 **Think about your financial situation in Stamola.** Given how much money ${e://Field/opgenpeople} make, and how much money you make relative to others, **what would your romantic relationships with ${e://Field/opgenpeople} be like in Stamolean society?** Specifically, what would you want in a partner? **Reflect on these questions for 30 seconds, and record your thoughts using the text box below.** As you progress throughout the survey, answer any questions with these thoughts in mind. After 30 seconds, a button will appear in the lower right hand corner of the screen and you may proceed to the next part of the survey.

____

____

____

____

____

Q1177 Timing
First Click (1)
Last Click (2)
Page Submit (3)
Click Count (4)

End of Block: 2M:1F 10th Male (DC)

Start of Block: 3M:2F 90th Male (DC)

Q2162 Timing
First Click (1)
Last Click (2)
Page Submit (3)
Click Count (4)

instruct3M2F90M The video below will give you some information about the economy and your own financial situation in Stamola. Please watch the video carefully, because attention checks

are included on the following page. These attention checks will be used to confirm whether you've understood, and remembered, key details about the economy in Stamola and your own financial situation. A button will appear in the lower right hand corner of the screen once the video is finished, and you will be able to proceed to the next part of the survey.

video3M2F90M

Page Break

ratio3M2F90M Which of the following options best describes **the average gender gap** in Stamola**?**

- Men earn 2x more than women (1)
- Men earn 1.5x more than women (2)
- There is no difference between the average earnings of men and women (3)
- Women earn 1.5x more than men (4)
- Women earn 2x more than men (5)

---

wpercent3M2F90M How does **your** income compare to that of **women** in Stamola?

- I earn more than 25% of women (1)
- I earn more than 45% of women (2)
- I earn more than 65% of women (3)
- I earn more than 85% of women (4)
- I earn more than 99% of women (5)

---

mpercent3M2F90M How does **your** income compare to that of **men** in Stamola?

- I earn more than 10% of men (1)
- I earn more than 30% of men (2)
- I earn more than 50% of men (3)
- I earn more than 70% of men (4)
- I earn more than 90% of men (5)

Page Break

gap3M2F90M How **gender unequal** is income in Stamola?

- 1 Not at all gender unequal (1)
- 2 (2)
- 3 (3)
- 4 Moderately gender unequal (4)
- 5 (5)
- 6 (6)
- 7 Extremely gender unequal (7)

---

wwealth3M2F90M Compared to **women** in Stamola, how **wealthy** are **you**?

- 1 Extremely poor (1)
- 2 (2)
- 3 (3)
- 4 Neither poor nor wealthy (4)
- 5 (5)
- 6 (8)
- 7 Extremely wealthy (9)

---

mwealth3M2F90M Compared to **men** in Stamola, how **wealthy** are **you**?

- 1 Extremely poor (1)
- 2 (2)
- 3 (3)
- 4 Neither poor nor wealthy (4)
- 5 (5)
- 6 (8)
- 7 Extremely wealthy (9)

Page Break

Q1092

Q1133 **Think about your financial situation in Stamola.** Given how much money ${e://Field/opgenpeople} make, and how much money you make relative to others, **what would your romantic relationships with ${e://Field/opgenpeople} be like in Stamolean society?** Specifically, what would you want in a partner? **Reflect on these questions for 30 seconds, and record your thoughts using the text box below.** As you progress throughout the survey, answer any questions with these thoughts in mind. After 30 seconds, a button will appear in the lower right hand corner of the screen and you may proceed to the next part of the survey.

____

____

____

____

____

Q1178 Timing
First Click (1)
Last Click (2)
Page Submit (3)
Click Count (4)

End of Block: 3M:2F 90th Male (DC)

Start of Block: 3M:2F 70th Male (DC)

Q2171 Timing
First Click (1)
Last Click (2)
Page Submit (3)
Click Count (4)

instruct3M2F70M The video below will give you some information about the economy and your own financial situation in Stamola. Please watch the video carefully, because attention checks

are included on the following page. These attention checks will be used to confirm whether you've understood, and remembered, key details about the economy in Stamola and your own financial situation. A button will appear in the lower right hand corner of the screen once the video is finished, and you will be able to proceed to the next part of the survey.

video3M2F70M

Page Break

ratio3M2F70M Which of the following options best describes **the average gender gap** in Stamola**?**

- Men earn 2x more than women (1)
- Men earn 1.5x more than women (2)
- There is no difference between the average earnings of men and women (3)
- Women earn 1.5x more than men (4)
- Women earn 2x more than men (5)

---

wpercent3M2F70M How does **your** income compare to that of **women** in Stamola?

- I earn more than 25% of women (1)
- I earn more than 45% of women (2)
- I earn more than 65% of women (3)
- I earn more than 85% of women (4)
- I earn more than 99% of women (5)

---

mpercent3M2F70M How does **your** income compare to that of **men** in Stamola?

- I earn more than 10% of men (1)
- I earn more than 30% of men (2)
- I earn more than 50% of men (3)
- I earn more than 70% of men (4)
- I earn more than 90% of men (5)

Page Break

gap3M2F70M How **gender unequal** is income in Stamola?

- 1 Not at all gender unequal (1)
- 2 (2)
- 3 (3)
- 4 Moderately gender unequal (4)
- 5 (5)
- 6 (6)
- 7 Extremely gender unequal (7)

---

wwealth3M2F70M Compared to **women** in Stamola, how **wealthy** are **you**?

- 1 Extremely poor (1)
- 2 (2)
- 3 (3)
- 4 Neither poor nor wealthy (4)
- 5 (5)
- 6 (8)
- 7 Extremely wealthy (9)

---

mwealth3M2F70M Compared to **men** in Stamola, how **wealthy** are **you**?

- ○ 1 Extremely poor (1)
- ○ 2 (2)
- ○ 3 (3)
- ○ 4 Neither poor nor wealthy (4)
- ○ 5 (5)
- ○ 6 (8)
- ○ 7 Extremely wealthy (9)

Page Break

Q1094

Q1134 **Think about your financial situation in Stamola.** Given how much money ${e://Field/opgenpeople} make, and how much money you make relative to others, **what would your romantic relationships with ${e://Field/opgenpeople} be like in Stamolean society?** Specifically, what would you want in a partner? **Reflect on these questions for 30 seconds, and record your thoughts using the text box below.** As you progress throughout the survey, answer any questions with these thoughts in mind. After 30 seconds, a button will appear in the lower right hand corner of the screen and you may proceed to the next part of the survey.

____

____

____

____

____

Q1179 Timing
First Click (1)
Last Click (2)
Page Submit (3)
Click Count (4)

End of Block: 3M:2F 70th Male (DC)

Start of Block: 3M:2F 50th Male (DC)

Q2180 Timing
First Click (1)
Last Click (2)
Page Submit (3)
Click Count (4)

instruct3M2F50M The video below will give you some information about the economy and your own financial situation in Stamola. Please watch the video carefully, because attention checks

are included on the following page. These attention checks will be used to confirm whether you've understood, and remembered, key details about the economy in Stamola and your own financial situation. A button will appear in the lower right hand corner of the screen once the video is finished, and you will be able to proceed to the next part of the survey.

---

video3M2F50M

Page Break

ratio3M2F50M Which of the following options best describes **the average gender gap** in Stamola**?**

- ◯ Men earn 2x more than women (1)
- ◯ Men earn 1.5x more than women (2)
- ◯ There is no difference between the average earnings of men and women (3)
- ◯ Women earn 1.5x more than men (4)
- ◯ Women earn 2x more than men (5)

---

wpercent3M2F50M How does **your** income compare to that of **women** in Stamola?

- ◯ I earn more than 25% of women (1)
- ◯ I earn more than 45% of women (2)
- ◯ I earn more than 65% of women (3)
- ◯ I earn more than 85% of women (4)
- ◯ I earn more than 99% of women (5)

---

mpercent3M2F50M How does **your** income compare to that of **men** in Stamola?

- I earn more than 10% of men (1)
- I earn more than 30% of men (2)
- I earn more than 50% of men (3)
- I earn more than 70% of men (4)
- I earn more than 90% of men (5)

Page Break

gap3M2F50M How **gender unequal** is income in Stamola?

- 1 Not at all gender unequal (1)
- 2 (2)
- 3 (3)
- 4 Moderately gender unequal (4)
- 5 (5)
- 6 (6)
- 7 Extremely gender unequal (7)

---

wwealth3M2F50M Compared to **women** in Stamola, how **wealthy** are **you**?

- 1 Extremely poor (1)
- 2 (2)
- 3 (3)
- 4 Neither poor nor wealthy (4)
- 5 (5)
- 6 (8)
- 7 Extremely wealthy (9)

---

mwealth3M2F50M Compared to **men** in Stamola, how **wealthy** are **you**?

- 1 Extremely poor (1)
- 2 (2)
- 3 (3)
- 4 Neither poor nor wealthy (4)
- 5 (5)
- 6 (8)
- 7 Extremely wealthy (9)

Page Break

Q1096

Q1135 **Think about your financial situation in Stamola.** Given how much money ${e://Field/opgenpeople} make, and how much money you make relative to others, **what would your romantic relationships with ${e://Field/opgenpeople} be like in Stamolean society?** Specifically, what would you want in a partner? **Reflect on these questions for 30 seconds, and record your thoughts using the text box below.** As you progress throughout the survey, answer any questions with these thoughts in mind. After 30 seconds, a button will appear in the lower right hand corner of the screen and you may proceed to the next part of the survey.

____

____

____

____

____

Q1180 Timing
First Click (1)
Last Click (2)
Page Submit (3)
Click Count (4)

End of Block: 3M:2F 50th Male (DC)

Start of Block: 3M:2F 30th Male (DC)

Q2189 Timing
First Click (1)
Last Click (2)
Page Submit (3)
Click Count (4)

---

instruct3M2F30M The video below will give you some information about the economy and your own financial situation in Stamola. Please watch the video carefully, because attention checks

are included on the following page. These attention checks will be used to confirm whether you've understood, and remembered, key details about the economy in Stamola and your own financial situation. A button will appear in the lower right hand corner of the screen once the video is finished, and you will be able to proceed to the next part of the survey.

video3M2F30M

Page Break

ratio3M2F30M Which of the following options best describes **the average gender gap** in Stamola**?**

- ◯ Men earn 2x more than women (1)
- ◯ Men earn 1.5x more than women (2)
- ◯ There is no difference between the average earnings of men and women (3)
- ◯ Women earn 1.5x more than men (4)
- ◯ Women earn 2x more than men (5)

---

wpercent3M2F30M How does **your** income compare to that of **women** in Stamola?

- ◯ I earn more than 25% of women (1)
- ◯ I earn more than 45% of women (2)
- ◯ I earn more than 65% of women (3)
- ◯ I earn more than 85% of women (4)
- ◯ I earn more than 99% of women (5)

---

mpercent3M2F30M How does **your** income compare to that of **men** in Stamola?

- I earn more than 10% of men (1)
- I earn more than 30% of men (2)
- I earn more than 50% of men (3)
- I earn more than 70% of men (4)
- I earn more than 90% of men (5)

Page Break

gap3M2F30M How **gender unequal** is income in Stamola?

- 1 Not at all gender unequal (1)
- 2 (2)
- 3 (3)
- 4 Moderately gender unequal (4)
- 5 (5)
- 6 (6)
- 7 Extremely gender unequal (7)

---

wwealth3M2F30M Compared to **women** in Stamola, how **wealthy** are **you**?

- 1 Extremely poor (1)
- 2 (2)
- 3 (3)
- 4 Neither poor nor wealthy (4)
- 5 (5)
- 6 (8)
- 7 Extremely wealthy (9)

---

mwealth3M2F30M Compared to **men** in Stamola, how **wealthy** are **you**?

- ◯ 1 Extremely poor (1)
- ◯ 2 (2)
- ◯ 3 (3)
- ◯ 4 Neither poor nor wealthy (4)
- ◯ 5 (5)
- ◯ 6 (8)
- ◯ 7 Extremely wealthy (9)

Page Break

Q1098

Q1136 **Think about your financial situation in Stamola.** Given how much money ${e://Field/opgenpeople} make, and how much money you make relative to others, **what would your romantic relationships with ${e://Field/opgenpeople} be like in Stamolean society?** Specifically, what would you want in a partner? **Reflect on these questions for 30 seconds, and record your thoughts using the text box below.** As you progress throughout the survey, answer any questions with these thoughts in mind. After 30 seconds, a button will appear in the lower right hand corner of the screen and you may proceed to the next part of the survey.

____

____

____

____

____

Q1181 Timing
First Click (1)
Last Click (2)
Page Submit (3)
Click Count (4)

End of Block: 3M:2F 30th Male (DC)

Start of Block: 3M:2F 10th Male (DC)

Q2198 Timing
First Click (1)
Last Click (2)
Page Submit (3)
Click Count (4)

instruct3M2F10M The video below will give you some information about the economy and your own financial situation in Stamola. Please watch the video carefully, because attention checks

are included on the following page. These attention checks will be used to confirm whether you've understood, and remembered, key details about the economy in Stamola and your own financial situation. A button will appear in the lower right hand corner of the screen once the video is finished, and you will be able to proceed to the next part of the survey.

video3M2F10M

Page Break

ratio3M2F10M Which of the following options best describes **the average gender gap** in Stamola**?**

- Men earn 2x more than women (1)
- Men earn 1.5x more than women (2)
- There is no difference between the average earnings of men and women (3)
- Women earn 1.5x more than men (4)
- Women earn 2x more than men (5)

---

wpercent3M2F10M How does **your** income compare to that of **women** in Stamola?

- I earn more than 25% of women (1)
- I earn more than 45% of women (2)
- I earn more than 65% of women (3)
- I earn more than 85% of women (4)
- I earn more than 99% of women (5)

---

mpercent3M2F10M How does **your** income compare to that of **men** in Stamola?

- I earn more than 10% of men (1)
- I earn more than 30% of men (2)
- I earn more than 50% of men (3)
- I earn more than 70% of men (4)
- I earn more than 90% of men (5)

Page Break

gap3M2F10M How **gender unequal** is income in Stamola?

- 1 Not at all gender unequal (1)
- 2 (2)
- 3 (3)
- 4 Moderately gender unequal (4)
- 5 (5)
- 6 (6)
- 7 Extremely gender unequal (7)

---

wwealth3M2F10M Compared to **women** in Stamola, how **wealthy** are **you**?

- 1 Extremely poor (1)
- 2 (2)
- 3 (3)
- 4 Neither poor nor wealthy (4)
- 5 (5)
- 6 (8)
- 7 Extremely wealthy (9)

---

mwealth3M2F10M Compared to **men** in Stamola, how **wealthy** are **you**?

- 1 Extremely poor (1)
- 2 (2)
- 3 (3)
- 4 Neither poor nor wealthy (4)
- 5 (5)
- 6 (8)
- 7 Extremely wealthy (9)

Page Break

Q1100

Q1137 **Think about your financial situation in Stamola.** Given how much money ${e://Field/opgenpeople} make, and how much money you make relative to others, **what would your romantic relationships with ${e://Field/opgenpeople} be like in Stamolean society?** Specifically, what would you want in a partner? **Reflect on these questions for 30 seconds, and record your thoughts using the text box below.** As you progress throughout the survey, answer any questions with these thoughts in mind. After 30 seconds, a button will appear in the lower right hand corner of the screen and you may proceed to the next part of the survey.

____

____

____

____

____

Q1182 Timing
First Click (1)
Last Click (2)
Page Submit (3)
Click Count (4)

End of Block: 3M:2F 10th Male (DC)

Start of Block: 1M:1F 90th (DC)

Q2252 Timing
First Click (1)
Last Click (2)
Page Submit (3)
Click Count (4)

---

instruct1M1F90 The video below will give you some information about the economy and your own financial situation in Stamola. Please watch the video carefully, because attention checks

are included on the following page. These attention checks will be used to confirm whether you've understood, and remembered, key details about the economy in Stamola and your own financial situation. A button will appear in the lower right hand corner of the screen once the video is finished, and you will be able to proceed to the next part of the survey.

video1M1F90

Page Break

ratio1M1F90 Which of the following options best describes **the average gender gap** in Stamola**?**

- ○ Men earn 2x more than women (1)
- ○ Men earn 1.5x more than women (2)
- ○ There is no difference between the average earnings of men and women (3)
- ○ Women earn 1.5x more than men (4)
- ○ Women earn 2x more than men (5)

---

wpercent1M1F90 How does **your** income compare to that of **women** in Stamola?

- ○ I earn more than 10% of women (1)
- ○ I earn more than 30% of women (2)
- ○ I earn more than 50% of women (3)
- ○ I earn more than 70% of women (4)
- ○ I earn more than 90% of women (5)

---

mpercent1M1F90 How does **your** income compare to that of **men** in Stamola?

- I earn more than 10% of men (1)
- I earn more than 30% of men (2)
- I earn more than 50% of men (3)
- I earn more than 70% of men (4)
- I earn more than 90% of men (5)

Page Break

gap1M1F90 How **gender unequal** is income in Stamola?

- 1 Not at all gender unequal (1)
- 2 (2)
- 3 (3)
- 4 Moderately gender unequal (4)
- 5 (5)
- 6 (6)
- 7 Extremely gender unequal (7)

---

wwealth1M1F90 Compared to **women** in Stamola, how **wealthy** are **you**?

- 1 Extremely poor (1)
- 2 (2)
- 3 (3)
- 4 Neither poor nor wealthy (4)
- 5 (5)
- 6 (8)
- 7 Extremely wealthy (9)

---

mwealth1M1F90 Compared to **men** in Stamola, how **wealthy** are **you**?

- 1 Extremely poor (1)
- 2 (2)
- 3 (3)
- 4 Neither poor nor wealthy (4)
- 5 (5)
- 6 (8)
- 7 Extremely wealthy (9)

Page Break

Q1102

Q1138 **Think about your financial situation in Stamola.** Given how much money ${e://Field/opgenpeople} make, and how much money you make relative to others, **what would your romantic relationships with ${e://Field/opgenpeople} be like in Stamolean society?** Specifically, what would you want in a partner? **Reflect on these questions for 30 seconds, and record your thoughts using the text box below.** As you progress throughout the survey, answer any questions with these thoughts in mind. After 30 seconds, a button will appear in the lower right hand corner of the screen and you may proceed to the next part of the survey.

____

____

____

____

____

Q1183 Timing
First Click (1)
Last Click (2)
Page Submit (3)
Click Count (4)

End of Block: 1M:1F 90th (DC)

Start of Block: 1M:1F 70th (DC)

Q2261 Timing
First Click (1)
Last Click (2)
Page Submit (3)
Click Count (4)

instruct1M1F70 The video below will give you some information about the economy and your own financial situation in Stamola. Please watch the video carefully, because attention checks

are included on the following page. These attention checks will be used to confirm whether you've understood, and remembered, key details about the economy in Stamola and your own financial situation. A button will appear in the lower right hand corner of the screen once the video is finished, and you will be able to proceed to the next part of the survey.

video1M1F70

Page Break

ratio1M1F70 Which of the following options best describes **the average gender gap** in Stamola**?**

- Men earn 2x more than women (1)
- Men earn 1.5x more than women (2)
- There is no difference between the average earnings of men and women (3)
- Women earn 1.5x more than men (4)
- Women earn 2x more than men (5)

---

wpercent1M1F70 How does **your** income compare to that of **women** in Stamola?

- I earn more than 10% of women (1)
- I earn more than 30% of women (2)
- I earn more than 50% of women (3)
- I earn more than 70% of women (4)
- I earn more than 90% of women (5)

---

mpercent1M1F70 How does **your** income compare to that of **men** in Stamola?

- I earn more than 10% of men (1)
- I earn more than 30% of men (2)
- I earn more than 50% of men (3)
- I earn more than 70% of men (4)
- I earn more than 90% of men (5)

Page Break

gap1M1F70 How **gender unequal** is income in Stamola?

- 1 Not at all gender unequal (1)
- 2 (2)
- 3 (3)
- 4 Moderately gender unequal (4)
- 5 (5)
- 6 (6)
- 7 Extremely gender unequal (7)

---

wwealth1M1F70 Compared to **women** in Stamola, how **wealthy** are **you**?

- 1 Extremely poor (1)
- 2 (2)
- 3 (3)
- 4 Neither poor nor wealthy (4)
- 5 (5)
- 6 (8)
- 7 Extremely wealthy (9)

---

mwealth1M1F70 Compared to **men** in Stamola, how **wealthy** are **you**?

- 1 Extremely poor (1)
- 2 (2)
- 3 (3)
- 4 Neither poor nor wealthy (4)
- 5 (5)
- 6 (8)
- 7 Extremely wealthy (9)

Page Break

Q1103

Q1139 **Think about your financial situation in Stamola.** Given how much money ${e://Field/opgenpeople} make, and how much money you make relative to others, **what would your romantic relationships with ${e://Field/opgenpeople} be like in Stamolean society?** Specifically, what would you want in a partner? **Reflect on these questions for 30 seconds, and record your thoughts using the text box below.** As you progress throughout the survey, answer any questions with these thoughts in mind. After 30 seconds, a button will appear in the lower right hand corner of the screen and you may proceed to the next part of the survey.

____

____

____

____

____

Q1184 Timing
First Click (1)
Last Click (2)
Page Submit (3)
Click Count (4)

End of Block: 1M:1F 70th (DC)

Start of Block: 1M:1F 50th (DC)

Q2270 Timing
First Click (1)
Last Click (2)
Page Submit (3)
Click Count (4)

---

instruct1M1F50 The video below will give you some information about the economy and your own financial situation in Stamola. Please watch the video carefully, because attention checks

are included on the following page. These attention checks will be used to confirm whether you've understood, and remembered, key details about the economy in Stamola and your own financial situation. A button will appear in the lower right hand corner of the screen once the video is finished, and you will be able to proceed to the next part of the survey.

video1M1F50

Page Break

ratio1M1F50 Which of the following options best describes **the average gender gap** in Stamola**?**

- ◯ Men earn 2x more than women (1)
- ◯ Men earn 1.5x more than women (2)
- ◯ There is no difference between the average earnings of men and women (3)
- ◯ Women earn 1.5x more than men (4)
- ◯ Women earn 2x more than men (5)

---

wpercent1M1F50 How does **your** income compare to that of **women** in Stamola?

- ◯ I earn more than 10% of women (1)
- ◯ I earn more than 30% of women (2)
- ◯ I earn more than 50% of women (3)
- ◯ I earn more than 70% of women (4)
- ◯ I earn more than 90% of women (5)

---

mpercent1M1F50 How does **your** income compare to that of **men** in Stamola?

- I earn more than 10% of men (1)
- I earn more than 30% of men (2)
- I earn more than 50% of men (3)
- I earn more than 70% of men (4)
- I earn more than 90% of men (5)

Page Break

gap1M1F50 How **gender unequal** is income in Stamola?

- 1 Not at all gender unequal (1)
- 2 (2)
- 3 (3)
- 4 Moderately gender unequal (4)
- 5 (5)
- 6 (6)
- 7 Extremely gender unequal (7)

---

wwealth1M1F50 Compared to **women** in Stamola, how **wealthy** are **you**?

- 1 Extremely poor (1)
- 2 (2)
- 3 (3)
- 4 Neither poor nor wealthy (4)
- 5 (5)
- 6 (8)
- 7 Extremely wealthy (9)

---

mwealth1M1f50 Compared to **men** in Stamola, how **wealthy** are **you**?

- 1 Extremely poor (1)
- 2 (2)
- 3 (3)
- 4 Neither poor nor wealthy (4)
- 5 (5)
- 6 (8)
- 7 Extremely wealthy (9)

Page Break

Q1104

Q1140 **Think about your financial situation in Stamola.** Given how much money ${e://Field/opgenpeople} make, and how much money you make relative to others, **what would your romantic relationships with ${e://Field/opgenpeople} be like in Stamolean society?** Specifically, what would you want in a partner? **Reflect on these questions for 30 seconds, and record your thoughts using the text box below.** As you progress throughout the survey, answer any questions with these thoughts in mind. After 30 seconds, a button will appear in the lower right hand corner of the screen and you may proceed to the next part of the survey.

____

____

____

____

____

Q1185 Timing
First Click (1)
Last Click (2)
Page Submit (3)
Click Count (4)

End of Block: 1M:1F 50th (DC)

Start of Block: 1M:1F 30th (DC)

Q2279 Timing
First Click (1)
Last Click (2)
Page Submit (3)
Click Count (4)

instruct1M1F30 The video below will give you some information about the economy and your own financial situation in Stamola. Please watch the video carefully, because attention checks

are included on the following page. These attention checks will be used to confirm whether you've understood, and remembered, key details about the economy in Stamola and your own financial situation. A button will appear in the lower right hand corner of the screen once the video is finished, and you will be able to proceed to the next part of the survey.

---

video1M1F30

Page Break

ratio1M1F30 Which of the following options best describes **the average gender gap** in Stamola**?**

- Men earn 2x more than women (1)
- Men earn 1.5x more than women (2)
- There is no difference between the average earnings of men and women (3)
- Women earn 1.5x more than men (4)
- Women earn 2x more than men (5)

---

wpercent1M1F30 How does **your** income compare to that of **women** in Stamola?

- I earn more than 10% of women (1)
- I earn more than 30% of women (2)
- I earn more than 50% of women (3)
- I earn more than 70% of women (4)
- I earn more than 90% of women (5)

---

mpercent1M1F30 How does **your** income compare to that of **men** in Stamola?

- I earn more than 10% of men (1)
- I earn more than 30% of men (2)
- I earn more than 50% of men (3)
- I earn more than 70% of men (4)
- I earn more than 90% of men (5)

Page Break

gap1M1F30 How **gender unequal** is income in Stamola?

- 1 Not at all gender unequal (1)
- 2 (2)
- 3 (3)
- 4 Moderately gender unequal (4)
- 5 (5)
- 6 (6)
- 7 Extremely gender unequal (7)

---

wwealth1M1F30 Compared to **women** in Stamola, how **wealthy** are **you**?

- 1 Extremely poor (1)
- 2 (2)
- 3 (3)
- 4 Neither poor nor wealthy (4)
- 5 (5)
- 6 (8)
- 7 Extremely wealthy (9)

---

mwealth1M1F30 Compared to **men** in Stamola, how **wealthy** are **you**?

- ○ 1 Extremely poor (1)
- ○ 2 (2)
- ○ 3 (3)
- ○ 4 Neither poor nor wealthy (4)
- ○ 5 (5)
- ○ 6 (8)
- ○ 7 Extremely wealthy (9)

Page Break

Q1105

Q1141 **Think about your financial situation in Stamola.** Given how much money ${e://Field/opgenpeople} make, and how much money you make relative to others, **what would your romantic relationships with ${e://Field/opgenpeople} be like in Stamolean society?** Specifically, what would you want in a partner? **Reflect on these questions for 30 seconds, and record your thoughts using the text box below.** As you progress throughout the survey, answer any questions with these thoughts in mind. After 30 seconds, a button will appear in the lower right hand corner of the screen and you may proceed to the next part of the survey.

____

____

____

____

____

Q1186 Timing
First Click (1)
Last Click (2)
Page Submit (3)
Click Count (4)

End of Block: 1M:1F 30th (DC)

Start of Block: 1M:1F 10th (DC)

Q2288 Timing
First Click (1)
Last Click (2)
Page Submit (3)
Click Count (4)

instruct1M1F10 The video below will give you some information about the economy and your own financial situation in Stamola. Please watch the video carefully, because attention checks

are included on the following page. These attention checks will be used to confirm whether you've understood, and remembered, key details about the economy in Stamola and your own financial situation. A button will appear in the lower right hand corner of the screen once the video is finished, and you will be able to proceed to the next part of the survey.

---

video1M1F10

Page Break

ratio1M1F10 Which of the following options best describes **the average gender gap** in Stamola**?**

- Men earn 2x more than women (1)
- Men earn 1.5x more than women (2)
- There is no difference between the average earnings of men and women (3)
- Women earn 1.5x more than men (4)
- Women earn 2x more than men (5)

---

wpercent1M1F10 How does **your** income compare to that of **women** in Stamola?

- I earn more than 10% of women (1)
- I earn more than 30% of women (2)
- I earn more than 50% of women (3)
- I earn more than 70% of women (4)
- I earn more than 90% of women (5)

---

mpercent1M1F10 How does **your** income compare to that of **men** in Stamola?

- I earn more than 10% of men (1)
- I earn more than 30% of men (2)
- I earn more than 50% of men (3)
- I earn more than 70% of men (4)
- I earn more than 90% of men (5)

Page Break

gap1M1F10 How **gender unequal** is income in Stamola?

- 1 Not at all gender unequal (1)
- 2 (2)
- 3 (3)
- 4 Moderately gender unequal (4)
- 5 (5)
- 6 (6)
- 7 Extremely gender unequal (7)

---

wwealth1M1F10 Compared to **women** in Stamola, how **wealthy** are **you**?

- 1 Extremely poor (1)
- 2 (2)
- 3 (3)
- 4 Neither poor nor wealthy (4)
- 5 (5)
- 6 (8)
- 7 Extremely wealthy (9)

---

mwealth1M1F10 Compared to **men** in Stamola, how **wealthy** are **you**?

- 1 Extremely poor (1)
- 2 (2)
- 3 (3)
- 4 Neither poor nor wealthy (4)
- 5 (5)
- 6 (8)
- 7 Extremely wealthy (9)

Page Break

Q1106

Q1142 **Think about your financial situation in Stamola.** Given how much money ${e://Field/opgenpeople} make, and how much money you make relative to others, **what would your romantic relationships with ${e://Field/opgenpeople} be like in Stamolean society?** Specifically, what would you want in a partner? **Reflect on these questions for 30 seconds, and record your thoughts using the text box below.** As you progress throughout the survey, answer any questions with these thoughts in mind. After 30 seconds, a button will appear in the lower right hand corner of the screen and you may proceed to the next part of the survey.

____

____

____

____

____

Q1187 Timing
First Click (1)
Last Click (2)
Page Submit (3)
Click Count (4)

End of Block: 1M:1F 10th (DC)

Start of Block: 2M:3F 90th Male (DC)

Q2297 Timing
First Click (1)
Last Click (2)
Page Submit (3)
Click Count (4)

instruct2M3F90M The video below will give you some information about the economy and your own financial situation in Stamola. Please watch the video carefully, because attention checks

are included on the following page. These attention checks will be used to confirm whether you've understood, and remembered, key details about the economy in Stamola and your own financial situation. A button will appear in the lower right hand corner of the screen once the video is finished, and you will be able to proceed to the next part of the survey.

---

video2M3F90M

Page Break

ratio2M3F90M Which of the following options best describes **the average gender gap** in Stamola**?**

- Men earn 2x more than women (1)
- Men earn 1.5x more than women (2)
- There is no difference between the average earnings of men and women (3)
- Women earn 1.5x more than men (4)
- Women earn 2x more than men (5)

---

wpercent2M3F90M How does **your** income compare to that of **women** in Stamola?

- I earn more than <1% of women (1)
- I earn more than 10% of women (2)
- I earn more than 30% of women (3)
- I earn more than 50% of women (4)
- I earn more than 70% of women (5)

---

mpercent2M3F90M How does **your** income compare to that of **men** in Stamola?

- I earn more than 10% of men (1)
- I earn more than 30% of men (2)
- I earn more than 50% of men (3)
- I earn more than 70% of men (4)
- I earn more than 90% of men (5)

Page Break

gap2M3F90M How **gender unequal** is income in Stamola?

- 1 Not at all gender unequal (1)
- 2 (2)
- 3 (3)
- 4 Moderately gender unequal (4)
- 5 (5)
- 6 (6)
- 7 Extremely gender unequal (7)

---

wwealth2M3F90M Compared to **women** in Stamola, how **wealthy** are **you**?

- 1 Extremely poor (1)
- 2 (2)
- 3 (3)
- 4 Neither poor nor wealthy (4)
- 5 (5)
- 6 (8)
- 7 Extremely wealthy (9)

---

mwealth2M3F90M Compared to **men** in Stamola, how **wealthy** are **you**?

- 1 Extremely poor (1)
- 2 (2)
- 3 (3)
- 4 Neither poor nor wealthy (4)
- 5 (5)
- 6 (8)
- 7 Extremely wealthy (9)

Page Break

Q1107

Q1143 **Think about your financial situation in Stamola.** Given how much money ${e://Field/opgenpeople} make, and how much money you make relative to others, **what would your romantic relationships with ${e://Field/opgenpeople} be like in Stamolean society?** Specifically, what would you want in a partner? **Reflect on these questions for 30 seconds, and record your thoughts using the text box below.** As you progress throughout the survey, answer any questions with these thoughts in mind. After 30 seconds, a button will appear in the lower right hand corner of the screen and you may proceed to the next part of the survey.

____

____

____

____

____

Q1188 Timing
First Click (1)
Last Click (2)
Page Submit (3)
Click Count (4)

End of Block: 2M:3F 90th Male (DC)

Start of Block: 2M:3F 70th Male (DC)

Q2306 Timing
First Click (1)
Last Click (2)
Page Submit (3)
Click Count (4)

instruct2M3F70M The video below will give you some information about the economy and your own financial situation in Stamola. Please watch the video carefully, because attention checks

are included on the following page. These attention checks will be used to confirm whether you've understood, and remembered, key details about the economy in Stamola and your own financial situation. A button will appear in the lower right hand corner of the screen once the video is finished, and you will be able to proceed to the next part of the survey.

---

video2M3F70M

Page Break

ratio2M3F70M Which of the following options best describes **the average gender gap** in Stamola**?**

- Men earn 2x more than women (1)
- Men earn 1.5x more than women (2)
- There is no difference between the average earnings of men and women (3)
- Women earn 1.5x more than men (4)
- Women earn 2x more than men (5)

---

wpercent2M3F70M How does **your** income compare to that of **women** in Stamola?

- I earn more than <1% of women (1)
- I earn more than 10% of women (2)
- I earn more than 30% of women (3)
- I earn more than 50% of women (4)
- I earn more than 70% of women (5)

---

mpercent2M3F70M How does **your** income compare to that of **men** in Stamola?

- I earn more than 10% of men (1)
- I earn more than 30% of men (2)
- I earn more than 50% of men (3)
- I earn more than 70% of men (4)
- I earn more than 90% of men (5)

Page Break

gap2M3F70M How **gender unequal** is income in Stamola?

- 1 Not at all gender unequal (1)
- 2 (2)
- 3 (3)
- 4 Moderately gender unequal (4)
- 5 (5)
- 6 (6)
- 7 Extremely gender unequal (7)

---

wwealth2M3F70M Compared to **women** in Stamola, how **wealthy** are **you**?

- 1 Extremely poor (1)
- 2 (2)
- 3 (3)
- 4 Neither poor nor wealthy (4)
- 5 (5)
- 6 (8)
- 7 Extremely wealthy (9)

---

mwealth2M3F70M Compared to **men** in Stamola, how **wealthy** are **you**?

- ○ 1 Extremely poor (1)
- ○ 2 (2)
- ○ 3 (3)
- ○ 4 Neither poor nor wealthy (4)
- ○ 5 (5)
- ○ 6 (8)
- ○ 7 Extremely wealthy (9)

Page Break

Q1110

Q1144 **Think about your financial situation in Stamola.** Given how much money ${e://Field/opgenpeople} make, and how much money you make relative to others, **what would your romantic relationships with ${e://Field/opgenpeople} be like in Stamolean society?** Specifically, what would you want in a partner? **Reflect on these questions for 30 seconds, and record your thoughts using the text box below.** As you progress throughout the survey, answer any questions with these thoughts in mind. After 30 seconds, a button will appear in the lower right hand corner of the screen and you may proceed to the next part of the survey.

____

____

____

____

____

Q1189 Timing
First Click (1)
Last Click (2)
Page Submit (3)
Click Count (4)

End of Block: 2M:3F 70th Male (DC)

Start of Block: 2M:3F 50th Male (DC)

Q2315 Timing
First Click (1)
Last Click (2)
Page Submit (3)
Click Count (4)

instruct2M3F50M The video below will give you some information about the economy and your own financial situation in Stamola. Please watch the video carefully, because attention checks

are included on the following page. These attention checks will be used to confirm whether you've understood, and remembered, key details about the economy in Stamola and your own financial situation. A button will appear in the lower right hand corner of the screen once the video is finished, and you will be able to proceed to the next part of the survey.

---

video2M3F50M

Page Break

ratio2M3F50M Which of the following options best describes **the average gender gap** in Stamola**?**

- Men earn 2x more than women (1)
- Men earn 1.5x more than women (2)
- There is no difference between the average earnings of men and women (3)
- Women earn 1.5x more than men (4)
- Women earn 2x more than men (5)

---

wpercent2M3F50M How does **your** income compare to that of **women** in Stamola?

- I earn more than <1% of women (1)
- I earn more than 10% of women (2)
- I earn more than 30% of women (3)
- I earn more than 50% of women (4)
- I earn more than 70% of women (5)

---

mpercent2M3F50M How does **your** income compare to that of **men** in Stamola?

- ○ I earn more than 10% of men (1)
- ○ I earn more than 30% of men (2)
- ○ I earn more than 50% of men (3)
- ○ I earn more than 70% of men (4)
- ○ I earn more than 90% of men (5)

Page Break

gap2M3F50M How **gender unequal** is income in Stamola?

- 1 Not at all gender unequal (1)
- 2 (2)
- 3 (3)
- 4 Moderately gender unequal (4)
- 5 (5)
- 6 (6)
- 7 Extremely gender unequal (7)

---

wwealth2M3F50M Compared to **women** in Stamola, how **wealthy** are **you**?

- 1 Extremely poor (1)
- 2 (2)
- 3 (3)
- 4 Neither poor nor wealthy (4)
- 5 (5)
- 6 (8)
- 7 Extremely wealthy (9)

---

mwealth2M3F50M Compared to **men** in Stamola, how **wealthy** are **you**?

- 1 Extremely poor (1)
- 2 (2)
- 3 (3)
- 4 Neither poor nor wealthy (4)
- 5 (5)
- 6 (8)
- 7 Extremely wealthy (9)

Page Break

Q1112

Q1145 **Think about your financial situation in Stamola.** Given how much money ${e://Field/opgenpeople} make, and how much money you make relative to others, **what would your romantic relationships with ${e://Field/opgenpeople} be like in Stamolean society?** Specifically, what would you want in a partner? **Reflect on these questions for 30 seconds, and record your thoughts using the text box below.** As you progress throughout the survey, answer any questions with these thoughts in mind. After 30 seconds, a button will appear in the lower right hand corner of the screen and you may proceed to the next part of the survey.

____

____

____

____

____

Q1190 Timing
First Click (1)
Last Click (2)
Page Submit (3)
Click Count (4)

End of Block: 2M:3F 50th Male (DC)

Start of Block: 2M:3F 30th Male (DC)

Q2324 Timing
First Click (1)
Last Click (2)
Page Submit (3)
Click Count (4)

---

instruct2M3F30M The video below will give you some information about the economy and your own financial situation in Stamola. Please watch the video carefully, because attention checks

are included on the following page. These attention checks will be used to confirm whether you've understood, and remembered, key details about the economy in Stamola and your own financial situation. A button will appear in the lower right hand corner of the screen once the video is finished, and you will be able to proceed to the next part of the survey.

---

video2M3F30M

Page Break

ratio2M3F30M Which of the following options best describes **the average gender gap** in Stamola**?**

- Men earn 2x more than women (1)
- Men earn 1.5x more than women (2)
- There is no difference between the average earnings of men and women (3)
- Women earn 1.5x more than men (4)
- Women earn 2x more than men (5)

---

wpercent2M3F30M How does **your** income compare to that of **women** in Stamola?

- I earn more than <1% of women (1)
- I earn more than 10% of women (2)
- I earn more than 30% of women (3)
- I earn more than 50% of women (4)
- I earn more than 70% of women (5)

---

mpercent2M3F30M How does **your** income compare to that of **men** in Stamola?

- I earn more than 10% of men (1)
- I earn more than 30% of men (2)
- I earn more than 50% of men (3)
- I earn more than 70% of men (4)
- I earn more than 90% of men (5)

Page Break

gap2M3F30M How **gender unequal** is income in Stamola?

- 1 Not at all gender unequal (1)
- 2 (2)
- 3 (3)
- 4 Moderately gender unequal (4)
- 5 (5)
- 6 (6)
- 7 Extremely gender unequal (7)

---

wwealth2M3F30M Compared to **women** in Stamola, how **wealthy** are **you**?

- 1 Extremely poor (1)
- 2 (2)
- 3 (3)
- 4 Neither poor nor wealthy (4)
- 5 (5)
- 6 (8)
- 7 Extremely wealthy (9)

---

mwealth2M3F30M Compared to **men** in Stamola, how **wealthy** are **you**?

- 1 Extremely poor (1)
- 2 (2)
- 3 (3)
- 4 Neither poor nor wealthy (4)
- 5 (5)
- 6 (8)
- 7 Extremely wealthy (9)

Page Break

Q1114

Q1146 **Think about your financial situation in Stamola.** Given how much money ${e://Field/opgenpeople} make, and how much money you make relative to others, **what would your romantic relationships with ${e://Field/opgenpeople} be like in Stamolean society?** Specifically, what would you want in a partner? **Reflect on these questions for 30 seconds, and record your thoughts using the text box below.** As you progress throughout the survey, answer any questions with these thoughts in mind. After 30 seconds, a button will appear in the lower right hand corner of the screen and you may proceed to the next part of the survey.

____

____

____

____

____

Q1191 Timing
First Click (1)
Last Click (2)
Page Submit (3)
Click Count (4)

End of Block: 2M:3F 30th Male (DC)

Start of Block: 2M:3F 10th Male (DC)

Q2333 Timing
First Click (1)
Last Click (2)
Page Submit (3)
Click Count (4)

instruct2M3F10M The video below will give you some information about the economy and your own financial situation in Stamola. Please watch the video carefully, because attention checks

are included on the following page. These attention checks will be used to confirm whether you've understood, and remembered, key details about the economy in Stamola and your own financial situation. A button will appear in the lower right hand corner of the screen once the video is finished, and you will be able to proceed to the next part of the survey.

---

video2M3F10M

Page Break

ratio2M3F10M Which of the following options best describes **the average gender gap** in Stamola**?**

- Men earn 2x more than women (1)
- Men earn 1.5x more than women (2)
- There is no difference between the average earnings of men and women (3)
- Women earn 1.5x more than men (4)
- Women earn 2x more than men (5)

---

wpercent2M3F10M How does **your** income compare to that of **women** in Stamola?

- I earn more than <1% of women (1)
- I earn more than 10% of women (2)
- I earn more than 30% of women (3)
- I earn more than 50% of women (4)
- I earn more than 70% of women (5)

---

mpercent2M3F10M How does **your** income compare to that of **men** in Stamola?

- ○ I earn more than 10% of men (1)
- ○ I earn more than 30% of men (2)
- ○ I earn more than 50% of men (3)
- ○ I earn more than 70% of men (4)
- ○ I earn more than 90% of men (5)

Page Break

gap2M3F10M How **gender unequal** is income in Stamola?

- 1 Not at all gender unequal (1)
- 2 (2)
- 3 (3)
- 4 Moderately gender unequal (4)
- 5 (5)
- 6 (6)
- 7 Extremely gender unequal (7)

---

wwealth2M3F10M Compared to **women** in Stamola, how **wealthy** are **you**?

- 1 Extremely poor (1)
- 2 (2)
- 3 (3)
- 4 Neither poor nor wealthy (4)
- 5 (5)
- 6 (8)
- 7 Extremely wealthy (9)

---

mwealth2M3F10M Compared to **men** in Stamola, how **wealthy** are **you**?

- 1 Extremely poor (1)
- 2 (2)
- 3 (3)
- 4 Neither poor nor wealthy (4)
- 5 (5)
- 6 (8)
- 7 Extremely wealthy (9)

Page Break

Q1116

Q1147 **Think about your financial situation in Stamola.** Given how much money ${e://Field/opgenpeople} make, and how much money you make relative to others, **what would your romantic relationships with ${e://Field/opgenpeople} be like in Stamolean society?** Specifically, what would you want in a partner? **Reflect on these questions for 30 seconds, and record your thoughts using the text box below.** As you progress throughout the survey, answer any questions with these thoughts in mind. After 30 seconds, a button will appear in the lower right hand corner of the screen and you may proceed to the next part of the survey.

____

____

____

____

____

Q1192 Timing
First Click (1)
Last Click (2)
Page Submit (3)
Click Count (4)

End of Block: 2M:3F 10th Male (DC)

Start of Block: 1M:2F 90th Male (DC)

Q2387 Timing
First Click (1)
Last Click (2)
Page Submit (3)
Click Count (4)

---

instruct1M2F90M The video below will give you some information about the economy and your own financial situation in Stamola. Please watch the video carefully, because attention checks

are included on the following page. These attention checks will be used to confirm whether you've understood, and remembered, key details about the economy in Stamola and your own financial situation. A button will appear in the lower right hand corner of the screen once the video is finished, and you will be able to proceed to the next part of the survey.

video1M2F90M

Page Break

ratio1M2F90M Which of the following options best describes **the average gender gap** in Stamola**?**

- ◯ Men earn 2x more than women (1)
- ◯ Men earn 1.5x more than women (2)
- ◯ There is no difference between the average earnings of men and women (3)
- ◯ Women earn 1.5x more than men (4)
- ◯ Women earn 2x more than men (5)

---

wpercent1M2F90M How does **your** income compare to that of **women** in Stamola?

- ◯ I earn more than <1% of women (1)
- ◯ I earn more than 5% of women (2)
- ◯ I earn more than 20% of women (3)
- ◯ I earn more than 40% of women (4)
- ◯ I earn more than 60% of women (5)

---

mpercent1M2F90M How does **your** income compare to that of **men** in Stamola?

- I earn more than 10% of men (1)
- I earn more than 30% of men (2)
- I earn more than 50% of men (3)
- I earn more than 70% of men (4)
- I earn more than 90% of men (5)

Page Break

gap1M2F90M How **gender unequal** is income in Stamola?

- 1 Not at all gender unequal (1)
- 2 (2)
- 3 (3)
- 4 Moderately gender unequal (4)
- 5 (5)
- 6 (6)
- 7 Extremely gender unequal (7)

---

wwealth1M2F90M Compared to **women** in Stamola, how **wealthy** are **you**?

- 1 Extremely poor (1)
- 2 (2)
- 3 (3)
- 4 Neither poor nor wealthy (4)
- 5 (5)
- 6 (8)
- 7 Extremely wealthy (9)

---

mwealth1M2F90M Compared to **men** in Stamola, how **wealthy** are **you**?

- ○ 1 Extremely poor (1)
- ○ 2 (2)
- ○ 3 (3)
- ○ 4 Neither poor nor wealthy (4)
- ○ 5 (5)
- ○ 6 (8)
- ○ 7 Extremely wealthy (9)

Page Break

Q1118

Q1148 **Think about your financial situation in Stamola.** Given how much money ${e://Field/opgenpeople} make, and how much money you make relative to others, **what would your romantic relationships with ${e://Field/opgenpeople} be like in Stamolean society?** Specifically, what would you want in a partner? **Reflect on these questions for 30 seconds, and record your thoughts using the text box below.** As you progress throughout the survey, answer any questions with these thoughts in mind. After 30 seconds, a button will appear in the lower right hand corner of the screen and you may proceed to the next part of the survey.

____

____

____

____

____

Q1193 Timing
First Click (1)
Last Click (2)
Page Submit (3)
Click Count (4)

End of Block: 1M:2F 90th Male (DC)

Start of Block: 1M:2F 70th Male (DC)

Q2396 Timing
First Click (1)
Last Click (2)
Page Submit (3)
Click Count (4)

instruct1M2F70M The video below will give you some information about the economy and your own financial situation in Stamola. Please watch the video carefully, because attention checks

are included on the following page. These attention checks will be used to confirm whether you've understood, and remembered, key details about the economy in Stamola and your own financial situation. A button will appear in the lower right hand corner of the screen once the video is finished, and you will be able to proceed to the next part of the survey.

video1M2F70M

Page Break

ratio1M2F70M Which of the following options best describes **the average gender gap** in Stamola**?**

- Men earn 2x more than women (1)
- Men earn 1.5x more than women (2)
- There is no difference between the average earnings of men and women (3)
- Women earn 1.5x more than men (4)
- Women earn 2x more than men (5)

---

wpercent1M2F70M How does **your** income compare to that of **women** in Stamola?

- I earn more than <1% of women (1)
- I earn more than 5% of women (2)
- I earn more than 20% of women (3)
- I earn more than 40% of women (4)
- I earn more than 60% of women (5)

---

mpercent1M2F70M How does **your** income compare to that of **men** in Stamola?

- I earn more than 10% of men (1)
- I earn more than 30% of men (2)
- I earn more than 50% of men (3)
- I earn more than 70% of men (4)
- I earn more than 90% of men (5)

Page Break

gap1M2F70M How **gender unequal** is income in Stamola?

- 1 Not at all gender unequal (1)
- 2 (2)
- 3 (3)
- 4 Moderately gender unequal (4)
- 5 (5)
- 6 (6)
- 7 Extremely gender unequal (7)

---

wwealth1M2F70M Compared to **women** in Stamola, how **wealthy** are **you**?

- 1 Extremely poor (1)
- 2 (2)
- 3 (3)
- 4 Neither poor nor wealthy (4)
- 5 (5)
- 6 (8)
- 7 Extremely wealthy (9)

---

mwealth1M2F70M Compared to **men** in Stamola, how **wealthy** are **you**?

- 1 Extremely poor (1)
- 2 (2)
- 3 (3)
- 4 Neither poor nor wealthy (4)
- 5 (5)
- 6 (8)
- 7 Extremely wealthy (9)

Page Break

Q1120

Q1149 **Think about your financial situation in Stamola.** Given how much money ${e://Field/opgenpeople} make, and how much money you make relative to others, **what would your romantic relationships with ${e://Field/opgenpeople} be like in Stamolean society?** Specifically, what would you want in a partner? **Reflect on these questions for 30 seconds, and record your thoughts using the text box below.** As you progress throughout the survey, answer any questions with these thoughts in mind. After 30 seconds, a button will appear in the lower right hand corner of the screen and you may proceed to the next part of the survey.

____

____

____

____

____

Q1194 Timing
First Click (1)
Last Click (2)
Page Submit (3)
Click Count (4)

End of Block: 1M:2F 70th Male (DC)

Start of Block: 1M:2F 50th Male (DC)

Q2405 Timing
First Click (1)
Last Click (2)
Page Submit (3)
Click Count (4)

---

instruct1M2F50M The video below will give you some information about the economy and your own financial situation in Stamola. Please watch the video carefully, because attention checks

are included on the following page. These attention checks will be used to confirm whether you've understood, and remembered, key details about the economy in Stamola and your own financial situation. A button will appear in the lower right hand corner of the screen once the video is finished, and you will be able to proceed to the next part of the survey.

video1M2F50M

Page Break

ratio1M2F50M Which of the following options best describes **the average gender gap** in Stamola**?**

- ○ Men earn 2x more than women (1)
- ○ Men earn 1.5x more than women (2)
- ○ There is no difference between the average earnings of men and women (3)
- ○ Women earn 1.5x more than men (4)
- ○ Women earn 2x more than men (5)

---

wpercent1M2F50M How does **your** income compare to that of **women** in Stamola?

- ○ I earn more than <1% of women (1)
- ○ I earn more than 5% of women (2)
- ○ I earn more than 20% of women (3)
- ○ I earn more than 40% of women (4)
- ○ I earn more than 60% of women (5)

---

mpercent1M2F50M How does **your** income compare to that of **men** in Stamola?

- I earn more than 10% of men (1)
- I earn more than 30% of men (2)
- I earn more than 50% of men (3)
- I earn more than 70% of men (4)
- I earn more than 90% of men (5)

Page Break

gap1M2F50M How **gender unequal** is income in Stamola?

- 1 Not at all gender unequal (1)
- 2 (2)
- 3 (3)
- 4 Moderately gender unequal (4)
- 5 (5)
- 6 (6)
- 7 Extremely gender unequal (7)

---

wwealth1M2F50M Compared to **women** in Stamola, how **wealthy** are **you**?

- 1 Extremely poor (1)
- 2 (2)
- 3 (3)
- 4 Neither poor nor wealthy (4)
- 5 (5)
- 6 (8)
- 7 Extremely wealthy (9)

---

mwealth1M2F50M Compared to **men** in Stamola, how **wealthy** are **you**?

- 1 Extremely poor (1)
- 2 (2)
- 3 (3)
- 4 Neither poor nor wealthy (4)
- 5 (5)
- 6 (8)
- 7 Extremely wealthy (9)

Page Break

Q1122

Q1150 **Think about your financial situation in Stamola.** Given how much money ${e://Field/opgenpeople} make, and how much money you make relative to others, **what would your romantic relationships with ${e://Field/opgenpeople} be like in Stamolean society?** Specifically, what would you want in a partner? **Reflect on these questions for 30 seconds, and record your thoughts using the text box below.** As you progress throughout the survey, answer any questions with these thoughts in mind. After 30 seconds, a button will appear in the lower right hand corner of the screen and you may proceed to the next part of the survey.

____

____

____

____

____

Q1195 Timing
First Click (1)
Last Click (2)
Page Submit (3)
Click Count (4)

End of Block: 1M:2F 50th Male (DC)

Start of Block: 1M:2F 30th Male (DC)

Q2414 Timing
First Click (1)
Last Click (2)
Page Submit (3)
Click Count (4)

instruct1M2F30M The video below will give you some information about the economy and your own financial situation in Stamola. Please watch the video carefully, because attention checks

are included on the following page. These attention checks will be used to confirm whether you've understood, and remembered, key details about the economy in Stamola and your own financial situation. A button will appear in the lower right hand corner of the screen once the video is finished, and you will be able to proceed to the next part of the survey.

video1M2F30M

Page Break

ratio1M2F30M Which of the following options best describes **the average gender gap** in Stamola**?**

- Men earn 2x more than women (1)
- Men earn 1.5x more than women (2)
- There is no difference between the average earnings of men and women (3)
- Women earn 1.5x more than men (4)
- Women earn 2x more than men (5)

---

wpercent1M2F30M How does **your** income compare to that of **women** in Stamola?

- I earn more than <1% of women (1)
- I earn more than 5% of women (2)
- I earn more than 20% of women (3)
- I earn more than 40% of women (4)
- I earn more than 60% of women (5)

---

mpercent1M2F30M How does **your** income compare to that of **men** in Stamola?

- I earn more than 10% of men (1)
- I earn more than 30% of men (2)
- I earn more than 50% of men (3)
- I earn more than 70% of men (4)
- I earn more than 90% of men (5)

Page Break

gap1M2F30M How **gender unequal** is income in Stamola?

- 1 Not at all gender unequal (1)
- 2 (2)
- 3 (3)
- 4 Moderately gender unequal (4)
- 5 (5)
- 6 (6)
- 7 Extremely gender unequal (7)

---

wwealth1M2F30M Compared to **women** in Stamola, how **wealthy** are **you**?

- 1 Extremely poor (1)
- 2 (2)
- 3 (3)
- 4 Neither poor nor wealthy (4)
- 5 (5)
- 6 (8)
- 7 Extremely wealthy (9)

---

mwealth1M2F30M Compared to **men** in Stamola, how **wealthy** are **you**?

- 1 Extremely poor (1)
- 2 (2)
- 3 (3)
- 4 Neither poor nor wealthy (4)
- 5 (5)
- 6 (8)
- 7 Extremely wealthy (9)

Page Break

Q1124

Q1151 **Think about your financial situation in Stamola.** Given how much money ${e://Field/opgenpeople} make, and how much money you make relative to others, **what would your romantic relationships with ${e://Field/opgenpeople} be like in Stamolean society?** Specifically, what would you want in a partner? **Reflect on these questions for 30 seconds, and record your thoughts using the text box below.** As you progress throughout the survey, answer any questions with these thoughts in mind. After 30 seconds, a button will appear in the lower right hand corner of the screen and you may proceed to the next part of the survey.

____

____

____

____

____

Q1196 Timing
First Click (1)
Last Click (2)
Page Submit (3)
Click Count (4)

End of Block: 1M:2F 30th Male (DC)

Start of Block: 1M:2F 10th Male (DC)

Q2423 Timing
First Click (1)
Last Click (2)
Page Submit (3)
Click Count (4)

---

instruct1M2F10M The video below will give you some information about the economy and your own financial situation in Stamola. Please watch the video carefully, because attention checks

are included on the following page. These attention checks will be used to confirm whether you've understood, and remembered, key details about the economy in Stamola and your own financial situation. A button will appear in the lower right hand corner of the screen once the video is finished, and you will be able to proceed to the next part of the survey.

---

video1M2F10M

Page Break

ratio1M2F10M Which of the following options best describes **the average gender gap** in Stamola**?**

- Men earn 2x more than women (1)
- Men earn 1.5x more than women (2)
- There is no difference between the average earnings of men and women (3)
- Women earn 1.5x more than men (4)
- Women earn 2x more than men (5)

---

wpercent1M2F10M How does **your** income compare to that of **women** in Stamola?

- I earn more than <1% of women (1)
- I earn more than 5% of women (2)
- I earn more than 20% of women (3)
- I earn more than 40% of women (4)
- I earn more than 60% of women (5)

---

mpercent1M2F10M How does **your** income compare to that of **men** in Stamola?

- I earn more than 10% of men (1)
- I earn more than 30% of men (2)
- I earn more than 50% of men (3)
- I earn more than 70% of men (4)
- I earn more than 90% of men (5)

Page Break

gap1M2F10M How **gender unequal** is income in Stamola?

- 1 Not at all gender unequal (1)
- 2 (2)
- 3 (3)
- 4 Moderately gender unequal (4)
- 5 (5)
- 6 (6)
- 7 Extremely gender unequal (7)

---

wwealth1M2F10M Compared to **women** in Stamola, how **wealthy** are **you**?

- 1 Extremely poor (1)
- 2 (2)
- 3 (3)
- 4 Neither poor nor wealthy (4)
- 5 (5)
- 6 (8)
- 7 Extremely wealthy (9)

---

mwealth1M2F10M Compared to **men** in Stamola, how **wealthy** are **you**?

- ○ 1 Extremely poor (1)
- ○ 2 (2)
- ○ 3 (3)
- ○ 4 Neither poor nor wealthy (4)
- ○ 5 (5)
- ○ 6 (8)
- ○ 7 Extremely wealthy (9)

Page Break

Q1126

Q1152 **Think about your financial situation in Stamola.** Given how much money ${e://Field/opgenpeople} make, and how much money you make relative to others, **what would your romantic relationships with ${e://Field/opgenpeople} be like in Stamolean society?** Specifically, what would you want in a partner? **Reflect on these questions for 30 seconds, and record your thoughts using the text box below.** As you progress throughout the survey, answer any questions with these thoughts in mind. After 30 seconds, a button will appear in the lower right hand corner of the screen and you may proceed to the next part of the survey.

____

____

____

____

____

Q1197 Timing
First Click (1)
Last Click (2)
Page Submit (3)
Click Count (4)

End of Block: 1M:2F 10th Male (DC)

Start of Block: 2M:1F 90th Female (DC)

Q2117 Timing
First Click (1)
Last Click (2)
Page Submit (3)
Click Count (4)

---

instruct2M1F90F The video below will give you some information about the economy and your own financial situation in Stamola. Please watch the video carefully, because attention checks

are included on the following page. These attention checks will be used to confirm whether you've understood, and remembered, key details about the economy in Stamola and your own financial situation. A button will appear in the lower right hand corner of the screen once the video is finished, and you will be able to proceed to the next part of the survey.

video2M1F90F

Page Break

ratio2M1F90F Which of the following options best describes **the average gender gap** in Stamola**?**

- Men earn 2x more than women (1)
- Men earn 1.5x more than women (2)
- There is no difference between the average earnings of men and women (3)
- Women earn 1.5x more than men (4)
- Women earn 2x more than men (5)

---

wpercent2M1F90F How does **your** income compare to that of **women** in Stamola?

- I earn more than 10% of women (1)
- I earn more than 30% of women (2)
- I earn more than 50% of women (3)
- I earn more than 70% of women (4)
- I earn more than 90% of women (5)

---

mpercent2M1F90F How does **your** income compare to that of **men** in Stamola?

- I earn more than <1% of men (1)
- I earn more than 5% of men (2)
- I earn more than 20% of men (3)
- I earn more than 40% of men (4)
- I earn more than 60% of men (5)

Page Break

gap2M1F90F How **gender unequal** is income in Stamola?

- 1 Not at all gender unequal (1)
- 2 (2)
- 3 (3)
- 4 Moderately gender unequal (4)
- 5 (5)
- 6 (6)
- 7 Extremely gender unequal (7)

---

wwealth2M1F90F Compared to **women** in Stamola, how **wealthy** are **you**?

- 1 Extremely poor (1)
- 2 (2)
- 3 (3)
- 4 Neither poor nor wealthy (4)
- 5 (5)
- 6 (8)
- 7 Extremely wealthy (9)

---

mwealth2M1F90F Compared to **men** in Stamola, how **wealthy** are **you**?

- 1 Extremely poor (1)
- 2 (2)
- 3 (3)
- 4 Neither poor nor wealthy (4)
- 5 (5)
- 6 (8)
- 7 Extremely wealthy (9)

Page Break

Q1083

Q1153 **Think about your financial situation in Stamola.** Given how much money ${e://Field/opgenpeople} make, and how much money you make relative to others, **what would your romantic relationships with ${e://Field/opgenpeople} be like in Stamolean society?** Specifically, what would you want in a partner? **Reflect on these questions for 30 seconds, and record your thoughts using the text box below.** As you progress throughout the survey, answer any questions with these thoughts in mind. After 30 seconds, a button will appear in the lower right hand corner of the screen and you may proceed to the next part of the survey.

____

____

____

____

____

Q1198 Timing
First Click (1)
Last Click (2)
Page Submit (3)
Click Count (4)

End of Block: 2M:1F 90th Female (DC)

Start of Block: 2M:1F 70th Female (DC)

Q2126 Timing
First Click (1)
Last Click (2)
Page Submit (3)
Click Count (4)

instruct2M1F70F The video below will give you some information about the economy and your own financial situation in Stamola. Please watch the video carefully, because attention checks

are included on the following page. These attention checks will be used to confirm whether you've understood, and remembered, key details about the economy in Stamola and your own financial situation. A button will appear in the lower right hand corner of the screen once the video is finished, and you will be able to proceed to the next part of the survey.

video2M1F70F

Page Break

ratio2M1F70F Which of the following options best describes **the average gender gap** in Stamola**?**

- ○ Men earn 2x more than women (1)
- ○ Men earn 1.5x more than women (2)
- ○ There is no difference between the average earnings of men and women (3)
- ○ Women earn 1.5x more than men (4)
- ○ Women earn 2x more than men (5)

---

wpercent2M1F70F How does **your** income compare to that of **women** in Stamola?

- ○ I earn more than 10% of women (1)
- ○ I earn more than 30% of women (2)
- ○ I earn more than 50% of women (3)
- ○ I earn more than 70% of women (4)
- ○ I earn more than 90% of women (5)

---

mpercent2M1F70F How does **your** income compare to that of **men** in Stamola?

- I earn more than <1% of men (1)
- I earn more than 5% of men (2)
- I earn more than 20% of men (3)
- I earn more than 40% of men (4)
- I earn more than 60% of men (5)

Page Break

gap2M1F70F How **gender unequal** is income in Stamola?

- 1 Not at all gender unequal (1)
- 2 (2)
- 3 (3)
- 4 Moderately gender unequal (4)
- 5 (5)
- 6 (6)
- 7 Extremely gender unequal (7)

---

wwealth2M1F70F Compared to **women** in Stamola, how **wealthy** are **you**?

- 1 Extremely poor (1)
- 2 (2)
- 3 (3)
- 4 Neither poor nor wealthy (4)
- 5 (5)
- 6 (8)
- 7 Extremely wealthy (9)

---

mwealth2M1F70F Compared to **men** in Stamola, how **wealthy** are **you**?

- 1 Extremely poor (1)
- 2 (2)
- 3 (3)
- 4 Neither poor nor wealthy (4)
- 5 (5)
- 6 (8)
- 7 Extremely wealthy (9)

Page Break

Q1085

Q1154 **Think about your financial situation in Stamola.** Given how much money ${e://Field/opgenpeople} make, and how much money you make relative to others, **what would your romantic relationships with ${e://Field/opgenpeople} be like in Stamolean society?** Specifically, what would you want in a partner? **Reflect on these questions for 30 seconds, and record your thoughts using the text box below.** As you progress throughout the survey, answer any questions with these thoughts in mind. After 30 seconds, a button will appear in the lower right hand corner of the screen and you may proceed to the next part of the survey.

____

____

____

____

____

Q1199 Timing
First Click (1)
Last Click (2)
Page Submit (3)
Click Count (4)

End of Block: 2M:1F 70th Female (DC)

Start of Block: 2M:1F 50th Female (DC)

Q2135 Timing
First Click (1)
Last Click (2)
Page Submit (3)
Click Count (4)

instruct2M1F50F The video below will give you some information about the economy and your own financial situation in Stamola. Please watch the video carefully, because attention checks

are included on the following page. These attention checks will be used to confirm whether you've understood, and remembered, key details about the economy in Stamola and your own financial situation. A button will appear in the lower right hand corner of the screen once the video is finished, and you will be able to proceed to the next part of the survey.

video2M1F50F

Page Break

ratio2M1F50F Which of the following options best describes **the average gender gap** in Stamola**?**

- Men earn 2x more than women (1)
- Men earn 1.5x more than women (2)
- There is no difference between the average earnings of men and women (3)
- Women earn 1.5x more than men (4)
- Women earn 2x more than men (5)

---

wpercent2M1F50F How does **your** income compare to that of **women** in Stamola?

- I earn more than 10% of women (1)
- I earn more than 30% of women (2)
- I earn more than 50% of women (3)
- I earn more than 70% of women (4)
- I earn more than 90% of women (5)

---

mpercent2M1F50F How does **your** income compare to that of **men** in Stamola?

- I earn more than <1% of men (1)
- I earn more than 5% of men (2)
- I earn more than 20% of men (3)
- I earn more than 40% of men (4)
- I earn more than 60% of men (5)

Page Break

gap2M1F50F How **gender unequal** is income in Stamola?

- 1 Not at all gender unequal (1)
- 2 (2)
- 3 (3)
- 4 Moderately gender unequal (4)
- 5 (5)
- 6 (6)
- 7 Extremely gender unequal (7)

---

wwealth2M1F50F Compared to **women** in Stamola, how **wealthy** are **you**?

- 1 Extremely poor (1)
- 2 (2)
- 3 (3)
- 4 Neither poor nor wealthy (4)
- 5 (5)
- 6 (8)
- 7 Extremely wealthy (9)

---

mwealth2M1F50F Compared to **men** in Stamola, how **wealthy** are **you**?

- 1 Extremely poor (1)
- 2 (2)
- 3 (3)
- 4 Neither poor nor wealthy (4)
- 5 (5)
- 6 (8)
- 7 Extremely wealthy (9)

Page Break

Q1087

Q1155 **Think about your financial situation in Stamola.** Given how much money ${e://Field/opgenpeople} make, and how much money you make relative to others, **what would your romantic relationships with ${e://Field/opgenpeople} be like in Stamolean society?** Specifically, what would you want in a partner? **Reflect on these questions for 30 seconds, and record your thoughts using the text box below.** As you progress throughout the survey, answer any questions with these thoughts in mind. After 30 seconds, a button will appear in the lower right hand corner of the screen and you may proceed to the next part of the survey.

____

____

____

____

____

Q1200 Timing
First Click (1)
Last Click (2)
Page Submit (3)
Click Count (4)

End of Block: 2M:1F 50th Female (DC)

Start of Block: 2M:1F 30th Female (DC)

Q2144 Timing
First Click (1)
Last Click (2)
Page Submit (3)
Click Count (4)

instruct2M1F30F The video below will give you some information about the economy and your own financial situation in Stamola. Please watch the video carefully, because attention checks

are included on the following page. These attention checks will be used to confirm whether you've understood, and remembered, key details about the economy in Stamola and your own financial situation. A button will appear in the lower right hand corner of the screen once the video is finished, and you will be able to proceed to the next part of the survey.

video2M1F30F

Page Break

ratio2M1F30F Which of the following options best describes **the average gender gap** in Stamola**?**

- Men earn 2x more than women (1)
- Men earn 1.5x more than women (2)
- There is no difference between the average earnings of men and women (3)
- Women earn 1.5x more than men (4)
- Women earn 2x more than men (5)

---

wpercent2M1F30F How does **your** income compare to that of **women** in Stamola?

- I earn more than 10% of women (1)
- I earn more than 30% of women (2)
- I earn more than 50% of women (3)
- I earn more than 70% of women (4)
- I earn more than 90% of women (5)

---

mpercent2M1F30F How does **your** income compare to that of **men** in Stamola?

- I earn more than <1% of men (1)
- I earn more than 5% of men (2)
- I earn more than 20% of men (3)
- I earn more than 40% of men (4)
- I earn more than 60% of men (5)

Page Break

gap2M1F30F How **gender unequal** is income in Stamola?

- 1 Not at all gender unequal (1)
- 2 (2)
- 3 (3)
- 4 Moderately gender unequal (4)
- 5 (5)
- 6 (6)
- 7 Extremely gender unequal (7)

---

wwealth2M1F30F Compared to **women** in Stamola, how **wealthy** are **you**?

- 1 Extremely poor (1)
- 2 (2)
- 3 (3)
- 4 Neither poor nor wealthy (4)
- 5 (5)
- 6 (8)
- 7 Extremely wealthy (9)

---

mwealth2M1F30F Compared to **men** in Stamola, how **wealthy** are **you**?

- 1 Extremely poor (1)
- 2 (2)
- 3 (3)
- 4 Neither poor nor wealthy (4)
- 5 (5)
- 6 (8)
- 7 Extremely wealthy (9)

Page Break

Q1089

Q1156 **Think about your financial situation in Stamola.** Given how much money ${e://Field/opgenpeople} make, and how much money you make relative to others, **what would your romantic relationships with ${e://Field/opgenpeople} be like in Stamolean society?** Specifically, what would you want in a partner? **Reflect on these questions for 30 seconds, and record your thoughts using the text box below.** As you progress throughout the survey, answer any questions with these thoughts in mind. After 30 seconds, a button will appear in the lower right hand corner of the screen and you may proceed to the next part of the survey.

____

____

____

____

____

Q1201 Timing
First Click (1)
Last Click (2)
Page Submit (3)
Click Count (4)

End of Block: 2M:1F 30th Female (DC)

Start of Block: 2M:1F 10th Female (DC)

Q2153 Timing
First Click (1)
Last Click (2)
Page Submit (3)
Click Count (4)

instruct2M1F10F The video below will give you some information about the economy and your own financial situation in Stamola. Please watch the video carefully, because attention checks

are included on the following page. These attention checks will be used to confirm whether you've understood, and remembered, key details about the economy in Stamola and your own financial situation. A button will appear in the lower right hand corner of the screen once the video is finished, and you will be able to proceed to the next part of the survey.

video2M1F10F

Page Break

ratio2M1F10F Which of the following options best describes **the average gender gap** in Stamola**?**

- ◯ Men earn 2x more than women (1)
- ◯ Men earn 1.5x more than women (2)
- ◯ There is no difference between the average earnings of men and women (3)
- ◯ Women earn 1.5x more than men (4)
- ◯ Women earn 2x more than men (5)

---

wpercent2M1F10F How does **your** income compare to that of **women** in Stamola?

- ◯ I earn more than 10% of women (1)
- ◯ I earn more than 30% of women (2)
- ◯ I earn more than 50% of women (3)
- ◯ I earn more than 70% of women (4)
- ◯ I earn more than 90% of women (5)

---

mpercent2M1F10F How does **your** income compare to that of **men** in Stamola?

- I earn more than <1% of men (1)
- I earn more than 5% of men (2)
- I earn more than 20% of men (3)
- I earn more than 40% of men (4)
- I earn more than 60% of men (5)

Page Break

gap2M1F10F How **gender unequal** is income in Stamola?

- 1 Not at all gender unequal (1)
- 2 (2)
- 3 (3)
- 4 Moderately gender unequal (4)
- 5 (5)
- 6 (6)
- 7 Extremely gender unequal (7)

---

wwealth2M1F10F Compared to **women** in Stamola, how **wealthy** are **you**?

- 1 Extremely poor (1)
- 2 (2)
- 3 (3)
- 4 Neither poor nor wealthy (4)
- 5 (5)
- 6 (8)
- 7 Extremely wealthy (9)

---

mwealth2M1F10F Compared to **men** in Stamola, how **wealthy** are **you**?

- 1 Extremely poor (1)
- 2 (2)
- 3 (3)
- 4 Neither poor nor wealthy (4)
- 5 (5)
- 6 (8)
- 7 Extremely wealthy (9)

Page Break

Q1091

Q1157 **Think about your financial situation in Stamola.** Given how much money ${e://Field/opgenpeople} make, and how much money you make relative to others, **what would your romantic relationships with ${e://Field/opgenpeople} be like in Stamolean society?** Specifically, what would you want in a partner? **Reflect on these questions for 30 seconds, and record your thoughts using the text box below.** As you progress throughout the survey, answer any questions with these thoughts in mind. After 30 seconds, a button will appear in the lower right hand corner of the screen and you may proceed to the next part of the survey.

____

____

____

____

____

Q1202 Timing
First Click (1)
Last Click (2)
Page Submit (3)
Click Count (4)

End of Block: 2M:1F 10th Female (DC)

Start of Block: 3M:2F 90th Female (DC)

Q2207 Timing
First Click (1)
Last Click (2)
Page Submit (3)
Click Count (4)

instruct3M2F90F The video below will give you some information about the economy and your own financial situation in Stamola. Please watch the video carefully, because attention checks

are included on the following page. These attention checks will be used to confirm whether you've understood, and remembered, key details about the economy in Stamola and your own financial situation. A button will appear in the lower right hand corner of the screen once the video is finished, and you will be able to proceed to the next part of the survey.

video3M2F90F

Page Break

ratio3M2F90F Which of the following options best describes **the average gender gap** in Stamola**?**

- Men earn 2x more than women (1)
- Men earn 1.5x more than women (2)
- There is no difference between the average earnings of men and women (3)
- Women earn 1.5x more than men (4)
- Women earn 2x more than men (5)

---

wpercent3M2F90F How does **your** income compare to that of **women** in Stamola?

- I earn more than 10% of women (1)
- I earn more than 30% of women (2)
- I earn more than 50% of women (3)
- I earn more than 70% of women (4)
- I earn more than 90% of women (5)

---

mpercent3M2F90F How does **your** income compare to that of **men** in Stamola?

- I earn more than <1% of men (1)
- I earn more than 10% of men (2)
- I earn more than 30% of men (3)
- I earn more than 50% of men (4)
- I earn more than 70% of men (5)

Page Break

gap3M2F90F How **gender unequal** is income in Stamola?

- 1 Not at all gender unequal (1)
- 2 (2)
- 3 (3)
- 4 Moderately gender unequal (4)
- 5 (5)
- 6 (6)
- 7 Extremely gender unequal (7)

---

wwealth3M2F90F Compared to **women** in Stamola, how **wealthy** are **you**?

- 1 Extremely poor (1)
- 2 (2)
- 3 (3)
- 4 Neither poor nor wealthy (4)
- 5 (5)
- 6 (8)
- 7 Extremely wealthy (9)

---

mwealth3M2F90F Compared to **men** in Stamola, how **wealthy** are **you**?

- 1 Extremely poor (1)
- 2 (2)
- 3 (3)
- 4 Neither poor nor wealthy (4)
- 5 (5)
- 6 (8)
- 7 Extremely wealthy (9)

Page Break

Q1093

Q1158 **Think about your financial situation in Stamola.** Given how much money ${e://Field/opgenpeople} make, and how much money you make relative to others, **what would your romantic relationships with ${e://Field/opgenpeople} be like in Stamolean society?** Specifically, what would you want in a partner? **Reflect on these questions for 30 seconds, and record your thoughts using the text box below.** As you progress throughout the survey, answer any questions with these thoughts in mind. After 30 seconds, a button will appear in the lower right hand corner of the screen and you may proceed to the next part of the survey.

____

____

____

____

____

Q1203 Timing
First Click (1)
Last Click (2)
Page Submit (3)
Click Count (4)

End of Block: 3M:2F 90th Female (DC)

Start of Block: 3M:2F 70th Female (DC)

Q2216 Timing
First Click (1)
Last Click (2)
Page Submit (3)
Click Count (4)

---

instruct3M2F70F The video below will give you some information about the economy and your own financial situation in Stamola. Please watch the video carefully, because attention checks

are included on the following page. These attention checks will be used to confirm whether you've understood, and remembered, key details about the economy in Stamola and your own financial situation. A button will appear in the lower right hand corner of the screen once the video is finished, and you will be able to proceed to the next part of the survey.

---

video3M2F70F

Page Break

ratio3M2F70F Which of the following options best describes **the average gender gap** in Stamola**?**

- ○ Men earn 2x more than women (1)
- ○ Men earn 1.5x more than women (2)
- ○ There is no difference between the average earnings of men and women (3)
- ○ Women earn 1.5x more than men (4)
- ○ Women earn 2x more than men (5)

---

wpercent3M2F70F How does **your** income compare to that of **women** in Stamola?

- ○ I earn more than 10% of women (1)
- ○ I earn more than 30% of women (2)
- ○ I earn more than 50% of women (3)
- ○ I earn more than 70% of women (4)
- ○ I earn more than 90% of women (5)

---

mpercent3M2F70F How does **your** income compare to that of **men** in Stamola?

- I earn more than <1% of men (1)
- I earn more than 10% of men (2)
- I earn more than 30% of men (3)
- I earn more than 50% of men (4)
- I earn more than 70% of men (5)

Page Break

gap3M2F70F How **gender unequal** is income in Stamola?

- 1 Not at all gender unequal (1)
- 2 (2)
- 3 (3)
- 4 Moderately gender unequal (4)
- 5 (5)
- 6 (6)
- 7 Extremely gender unequal (7)

---

wwealth3M2F70F Compared to **women** in Stamola, how **wealthy** are **you**?

- 1 Extremely poor (1)
- 2 (2)
- 3 (3)
- 4 Neither poor nor wealthy (4)
- 5 (5)
- 6 (8)
- 7 Extremely wealthy (9)

---

mwealth3M2F70F Compared to **men** in Stamola, how **wealthy** are **you**?

- 1 Extremely poor (1)
- 2 (2)
- 3 (3)
- 4 Neither poor nor wealthy (4)
- 5 (5)
- 6 (8)
- 7 Extremely wealthy (9)

Page Break

Q1095

Q1159 **Think about your financial situation in Stamola.** Given how much money ${e://Field/opgenpeople} make, and how much money you make relative to others, **what would your romantic relationships with ${e://Field/opgenpeople} be like in Stamolean society?** Specifically, what would you want in a partner? **Reflect on these questions for 30 seconds, and record your thoughts using the text box below.** As you progress throughout the survey, answer any questions with these thoughts in mind. After 30 seconds, a button will appear in the lower right hand corner of the screen and you may proceed to the next part of the survey.

____

____

____

____

____

Q1204 Timing
First Click (1)
Last Click (2)
Page Submit (3)
Click Count (4)

End of Block: 3M:2F 70th Female (DC)

Start of Block: 3M:2F 50th Female (DC)

Q2225 Timing
First Click (1)
Last Click (2)
Page Submit (3)
Click Count (4)

instruct3M2F50F The video below will give you some information about the economy and your own financial situation in Stamola. Please watch the video carefully, because attention checks

are included on the following page. These attention checks will be used to confirm whether you've understood, and remembered, key details about the economy in Stamola and your own financial situation. A button will appear in the lower right hand corner of the screen once the video is finished, and you will be able to proceed to the next part of the survey.

video3M2F50F

Page Break

ratio3M2F50F Which of the following options best describes **the average gender gap** in Stamola**?**

- ○ Men earn 2x more than women (1)
- ○ Men earn 1.5x more than women (2)
- ○ There is no difference between the average earnings of men and women (3)
- ○ Women earn 1.5x more than men (4)
- ○ Women earn 2x more than men (5)

---

wpercent3M2F50F How does **your** income compare to that of **women** in Stamola?

- ○ I earn more than 10% of women (1)
- ○ I earn more than 30% of women (2)
- ○ I earn more than 50% of women (3)
- ○ I earn more than 70% of women (4)
- ○ I earn more than 90% of women (5)

---

mpercent3M2F50F How does **your** income compare to that of **men** in Stamola?

- I earn more than <1% of men (1)
- I earn more than 10% of men (2)
- I earn more than 30% of men (3)
- I earn more than 50% of men (4)
- I earn more than 70% of men (5)

Page Break

gap3M2F50F How **gender unequal** is income in Stamola?

- 1 Not at all gender unequal (1)
- 2 (2)
- 3 (3)
- 4 Moderately gender unequal (4)
- 5 (5)
- 6 (6)
- 7 Extremely gender unequal (7)

---

wwealth3M2F50F Compared to **women** in Stamola, how **wealthy** are **you**?

- 1 Extremely poor (1)
- 2 (2)
- 3 (3)
- 4 Neither poor nor wealthy (4)
- 5 (5)
- 6 (8)
- 7 Extremely wealthy (9)

---

mwealth3M2F50F Compared to **men** in Stamola, how **wealthy** are **you**?

- 1 Extremely poor (1)
- 2 (2)
- 3 (3)
- 4 Neither poor nor wealthy (4)
- 5 (5)
- 6 (8)
- 7 Extremely wealthy (9)

Page Break

Q1161

Q1160 **Think about your financial situation in Stamola.** Given how much money ${e://Field/opgenpeople} make, and how much money you make relative to others, **what would your romantic relationships with ${e://Field/opgenpeople} be like in Stamolean society?** Specifically, what would you want in a partner? **Reflect on these questions for 30 seconds, and record your thoughts using the text box below.** As you progress throughout the survey, answer any questions with these thoughts in mind. After 30 seconds, a button will appear in the lower right hand corner of the screen and you may proceed to the next part of the survey.

____

____

____

____

____

Q1205 Timing
First Click (1)
Last Click (2)
Page Submit (3)
Click Count (4)

End of Block: 3M:2F 50th Female (DC)

Start of Block: 3M:2F 30th Female (DC)

Q2234 Timing
First Click (1)
Last Click (2)
Page Submit (3)
Click Count (4)

---

instruct3M2F30F The video below will give you some information about the economy and your own financial situation in Stamola. Please watch the video carefully, because attention checks

are included on the following page. These attention checks will be used to confirm whether you've understood, and remembered, key details about the economy in Stamola and your own financial situation. A button will appear in the lower right hand corner of the screen once the video is finished, and you will be able to proceed to the next part of the survey.

---

video3M2F30F

Page Break

ratio3M2F30F Which of the following options best describes **the average gender gap** in Stamola**?**

- Men earn 2x more than women (1)
- Men earn 1.5x more than women (2)
- There is no difference between the average earnings of men and women (3)
- Women earn 1.5x more than men (4)
- Women earn 2x more than men (5)

---

wpercent3M2F30F How does **your** income compare to that of **women** in Stamola?

- I earn more than 10% of women (1)
- I earn more than 30% of women (2)
- I earn more than 50% of women (3)
- I earn more than 70% of women (4)
- I earn more than 90% of women (5)

---

mpercent3M2F30F How does **your** income compare to that of **men** in Stamola?

- I earn more than <1% of men (1)
- I earn more than 10% of men (2)
- I earn more than 30% of men (3)
- I earn more than 50% of men (4)
- I earn more than 70% of men (5)

Page Break

gap3M2F30F How **gender unequal** is income in Stamola?

- 1 Not at all gender unequal (1)
- 2 (2)
- 3 (3)
- 4 Moderately gender unequal (4)
- 5 (5)
- 6 (6)
- 7 Extremely gender unequal (7)

---

wwealth3M2F30F Compared to **women** in Stamola, how **wealthy** are **you**?

- 1 Extremely poor (1)
- 2 (2)
- 3 (3)
- 4 Neither poor nor wealthy (4)
- 5 (5)
- 6 (8)
- 7 Extremely wealthy (9)

---

mwealth3M2F30F Compared to **men** in Stamola, how **wealthy** are **you**?

- 1 Extremely poor (1)
- 2 (2)
- 3 (3)
- 4 Neither poor nor wealthy (4)
- 5 (5)
- 6 (8)
- 7 Extremely wealthy (9)

Page Break

Q1099

Q1162 **Think about your financial situation in Stamola.** Given how much money ${e://Field/opgenpeople} make, and how much money you make relative to others, **what would your romantic relationships with ${e://Field/opgenpeople} be like in Stamolean society?** Specifically, what would you want in a partner? **Reflect on these questions for 30 seconds, and record your thoughts using the text box below.** As you progress throughout the survey, answer any questions with these thoughts in mind. After 30 seconds, a button will appear in the lower right hand corner of the screen and you may proceed to the next part of the survey.

____

____

____

____

____

Q1206 Timing
First Click (1)
Last Click (2)
Page Submit (3)
Click Count (4)

End of Block: 3M:2F 30th Female (DC)

Start of Block: 3M:2F 10th Female (DC)

Q2243 Timing
First Click (1)
Last Click (2)
Page Submit (3)
Click Count (4)

---

instruct3M2F10F The video below will give you some information about the economy and your own financial situation in Stamola. Please watch the video carefully, because attention checks

are included on the following page. These attention checks will be used to confirm whether you've understood, and remembered, key details about the economy in Stamola and your own financial situation. A button will appear in the lower right hand corner of the screen once the video is finished, and you will be able to proceed to the next part of the survey.

video3M2F10F

Page Break

ratio3M2F10F Which of the following options best describes **the average gender gap** in Stamola**?**

- ○ Men earn 2x more than women (1)
- ○ Men earn 1.5x more than women (2)
- ○ There is no difference between the average earnings of men and women (3)
- ○ Women earn 1.5x more than men (4)
- ○ Women earn 2x more than men (5)

---

wpercent3M2F10F How does **your** income compare to that of **women** in Stamola?

- ○ I earn more than 10% of women (1)
- ○ I earn more than 30% of women (2)
- ○ I earn more than 50% of women (3)
- ○ I earn more than 70% of women (4)
- ○ I earn more than 90% of women (5)

---

mpercent3M2F10F How does **your** income compare to that of **men** in Stamola?

- I earn more than <1% of men (1)
- I earn more than 10% of men (2)
- I earn more than 30% of men (3)
- I earn more than 50% of men (4)
- I earn more than 70% of men (5)

Page Break

gap3M2F10F How **gender unequal** is income in Stamola?

- 1 Not at all gender unequal (1)
- 2 (2)
- 3 (3)
- 4 Moderately gender unequal (4)
- 5 (5)
- 6 (6)
- 7 Extremely gender unequal (7)

---

wwealth3M2F10F Compared to **women** in Stamola, how **wealthy** are **you**?

- 1 Extremely poor (1)
- 2 (2)
- 3 (3)
- 4 Neither poor nor wealthy (4)
- 5 (5)
- 6 (8)
- 7 Extremely wealthy (9)

---

mwealth3M2F10F Compared to **men** in Stamola, how **wealthy** are **you**?

- ○ 1 Extremely poor (1)
- ○ 2 (2)
- ○ 3 (3)
- ○ 4 Neither poor nor wealthy (4)
- ○ 5 (5)
- ○ 6 (8)
- ○ 7 Extremely wealthy (9)

Page Break

Q1101

Q1163 **Think about your financial situation in Stamola.** Given how much money ${e://Field/opgenpeople} make, and how much money you make relative to others, **what would your romantic relationships with ${e://Field/opgenpeople} be like in Stamolean society?** Specifically, what would you want in a partner? **Reflect on these questions for 30 seconds, and record your thoughts using the text box below.** As you progress throughout the survey, answer any questions with these thoughts in mind. After 30 seconds, a button will appear in the lower right hand corner of the screen and you may proceed to the next part of the survey.

____

____

____

____

____

Q1207 Timing
First Click (1)
Last Click (2)
Page Submit (3)
Click Count (4)

End of Block: 3M:2F 10th Female (DC)

Start of Block: 2M:3F 90th Female (DC)

Q2342 Timing
First Click (1)
Last Click (2)
Page Submit (3)
Click Count (4)

---

instruct2M3F90F The video below will give you some information about the economy and your own financial situation in Stamola. Please watch the video carefully, because attention checks

are included on the following page. These attention checks will be used to confirm whether you've understood, and remembered, key details about the economy in Stamola and your own financial situation. A button will appear in the lower right hand corner of the screen once the video is finished, and you will be able to proceed to the next part of the survey.

---

video2M3F90F

Page Break

ratio2M3F90F Which of the following options best describes **the average gender gap** in Stamola**?**

- ◯ Men earn 2x more than women (1)
- ◯ Men earn 1.5x more than women (2)
- ◯ There is no difference between the average earnings of men and women (3)
- ◯ Women earn 1.5x more than men (4)
- ◯ Women earn 2x more than men (5)

---

wpercent2M3F90F How does **your** income compare to that of **women** in Stamola?

- ◯ I earn more than 10% of women (1)
- ◯ I earn more than 30% of women (2)
- ◯ I earn more than 50% of women (3)
- ◯ I earn more than 70% of women (4)
- ◯ I earn more than 90% of women (5)

---

mpercent2M3F90F How does **your** income compare to that of **men** in Stamola?

- I earn more than 25% of men (1)
- I earn more than 45% of men (2)
- I earn more than 65% of men (3)
- I earn more than 85% of men (4)
- I earn more than 99% of men (5)

Page Break

gap2M3F90F How **gender unequal** is income in Stamola?

- 1 Not at all gender unequal (1)
- 2 (2)
- 3 (3)
- 4 Moderately gender unequal (4)
- 5 (5)
- 6 (6)
- 7 Extremely gender unequal (7)

---

wwealth2M3F90F Compared to **women** in Stamola, how **wealthy** are **you**?

- 1 Extremely poor (1)
- 2 (2)
- 3 (3)
- 4 Neither poor nor wealthy (4)
- 5 (5)
- 6 (8)
- 7 Extremely wealthy (9)

---

mwealth2M3F90F Compared to **men** in Stamola, how **wealthy** are **you**?

- 1 Extremely poor (1)
- 2 (2)
- 3 (3)
- 4 Neither poor nor wealthy (4)
- 5 (5)
- 6 (8)
- 7 Extremely wealthy (9)

Page Break

Q1109

Q1164 **Think about your financial situation in Stamola.** Given how much money ${e://Field/opgenpeople} make, and how much money you make relative to others, **what would your romantic relationships with ${e://Field/opgenpeople} be like in Stamolean society?** Specifically, what would you want in a partner? **Reflect on these questions for 30 seconds, and record your thoughts using the text box below.** As you progress throughout the survey, answer any questions with these thoughts in mind. After 30 seconds, a button will appear in the lower right hand corner of the screen and you may proceed to the next part of the survey.

____

____

____

____

____

Q1208 Timing
First Click (1)
Last Click (2)
Page Submit (3)
Click Count (4)

End of Block: 2M:3F 90th Female (DC)

Start of Block: 2M:3F 70th Female (DC)

Q2351 Timing
First Click (1)
Last Click (2)
Page Submit (3)
Click Count (4)

---

instruct2M3F70F The video below will give you some information about the economy and your own financial situation in Stamola. Please watch the video carefully, because attention checks

are included on the following page. These attention checks will be used to confirm whether you've understood, and remembered, key details about the economy in Stamola and your own financial situation. A button will appear in the lower right hand corner of the screen once the video is finished, and you will be able to proceed to the next part of the survey.

---

video2M3F70F

Page Break

ratio2M3F70F Which of the following options best describes **the average gender gap** in Stamola**?**

- Men earn 2x more than women (1)
- Men earn 1.5x more than women (2)
- There is no difference between the average earnings of men and women (3)
- Women earn 1.5x more than men (4)
- Women earn 2x more than men (5)

---

wpercent2M3F70F How does **your** income compare to that of **women** in Stamola?

- I earn more than 10% of women (1)
- I earn more than 30% of women (2)
- I earn more than 50% of women (3)
- I earn more than 70% of women (4)
- I earn more than 90% of women (5)

---

mpercent2M3F70F How does **your** income compare to that of **men** in Stamola?

- I earn more than 25% of men (1)
- I earn more than 45% of men (2)
- I earn more than 65% of men (3)
- I earn more than 85% of men (4)
- I earn more than 99% of men (5)

Page Break

gap2M3F70F How **gender unequal** is income in Stamola?

- 1 Not at all gender unequal (1)
- 2 (2)
- 3 (3)
- 4 Moderately gender unequal (4)
- 5 (5)
- 6 (6)
- 7 Extremely gender unequal (7)

---

wwealth2M3F70F Compared to **women** in Stamola, how **wealthy** are **you**?

- 1 Extremely poor (1)
- 2 (2)
- 3 (3)
- 4 Neither poor nor wealthy (4)
- 5 (5)
- 6 (8)
- 7 Extremely wealthy (9)

---

mwealth2M3F70F Compared to **men** in Stamola, how **wealthy** are **you**?

- 1 Extremely poor (1)
- 2 (2)
- 3 (3)
- 4 Neither poor nor wealthy (4)
- 5 (5)
- 6 (8)
- 7 Extremely wealthy (9)

Page Break

Q1111

Q1165 **Think about your financial situation in Stamola.** Given how much money ${e://Field/opgenpeople} make, and how much money you make relative to others, **what would your romantic relationships with ${e://Field/opgenpeople} be like in Stamolean society?** Specifically, what would you want in a partner? **Reflect on these questions for 30 seconds, and record your thoughts using the text box below.** As you progress throughout the survey, answer any questions with these thoughts in mind. After 30 seconds, a button will appear in the lower right hand corner of the screen and you may proceed to the next part of the survey.

____

____

____

____

____

Q1209 Timing
First Click (1)
Last Click (2)
Page Submit (3)
Click Count (4)

End of Block: 2M:3F 70th Female (DC)

Start of Block: 2M:3F 50th Female (DC)

Q2360 Timing
First Click (1)
Last Click (2)
Page Submit (3)
Click Count (4)

instruct2M3F50F The video below will give you some information about the economy and your own financial situation in Stamola. Please watch the video carefully, because attention checks

are included on the following page. These attention checks will be used to confirm whether you've understood, and remembered, key details about the economy in Stamola and your own financial situation. A button will appear in the lower right hand corner of the screen once the video is finished, and you will be able to proceed to the next part of the survey.

video2M3F50F

Page Break

ratio2M3F50F Which of the following options best describes **the average gender gap** in Stamola**?**

- Men earn 2x more than women (1)
- Men earn 1.5x more than women (2)
- There is no difference between the average earnings of men and women (3)
- Women earn 1.5x more than men (4)
- Women earn 2x more than men (5)

---

wpercent2M3F50F How does **your** income compare to that of **women** in Stamola?

- I earn more than 10% of women (1)
- I earn more than 30% of women (2)
- I earn more than 50% of women (3)
- I earn more than 70% of women (4)
- I earn more than 90% of women (5)

---

mpercent2M3F50F How does **your** income compare to that of **men** in Stamola?

- I earn more than 25% of men (1)
- I earn more than 45% of men (2)
- I earn more than 65% of men (3)
- I earn more than 85% of men (4)
- I earn more than 99% of men (5)

Page Break

gap2M3F50F How **gender unequal** is income in Stamola?

- 1 Not at all gender unequal (1)
- 2 (2)
- 3 (3)
- 4 Moderately gender unequal (4)
- 5 (5)
- 6 (6)
- 7 Extremely gender unequal (7)

---

wwealth2M3F50F Compared to **women** in Stamola, how **wealthy** are **you**?

- 1 Extremely poor (1)
- 2 (2)
- 3 (3)
- 4 Neither poor nor wealthy (4)
- 5 (5)
- 6 (8)
- 7 Extremely wealthy (9)

---

mwealth2M3F50F Compared to **men** in Stamola, how **wealthy** are **you**?

- 1 Extremely poor (1)
- 2 (2)
- 3 (3)
- 4 Neither poor nor wealthy (4)
- 5 (5)
- 6 (8)
- 7 Extremely wealthy (9)

Page Break

Q1113

Q1166 **Think about your financial situation in Stamola.** Given how much money ${e://Field/opgenpeople} make, and how much money you make relative to others, **what would your romantic relationships with ${e://Field/opgenpeople} be like in Stamolean society?** Specifically, what would you want in a partner? **Reflect on these questions for 30 seconds, and record your thoughts using the text box below.** As you progress throughout the survey, answer any questions with these thoughts in mind. After 30 seconds, a button will appear in the lower right hand corner of the screen and you may proceed to the next part of the survey.

____

____

____

____

____

Q1210 Timing
First Click (1)
Last Click (2)
Page Submit (3)
Click Count (4)

End of Block: 2M:3F 50th Female (DC)

Start of Block: 2M:3F 30th Female (DC)

Q2369 Timing
First Click (1)
Last Click (2)
Page Submit (3)
Click Count (4)

instruct2M3F30F The video below will give you some information about the economy and your own financial situation in Stamola. Please watch the video carefully, because attention checks

are included on the following page. These attention checks will be used to confirm whether you've understood, and remembered, key details about the economy in Stamola and your own financial situation. A button will appear in the lower right hand corner of the screen once the video is finished, and you will be able to proceed to the next part of the survey.

---

video2M3F30F

Page Break

ratio2M3F30F Which of the following options best describes **the average gender gap** in Stamola**?**

- ○ Men earn 2x more than women (1)
- ○ Men earn 1.5x more than women (2)
- ○ There is no difference between the average earnings of men and women (3)
- ○ Women earn 1.5x more than men (4)
- ○ Women earn 2x more than men (5)

---

wpercent2M3F30F How does **your** income compare to that of **women** in Stamola?

- ○ I earn more than 10% of women (1)
- ○ I earn more than 30% of women (2)
- ○ I earn more than 50% of women (3)
- ○ I earn more than 70% of women (4)
- ○ I earn more than 90% of women (5)

---

mpercent2M3F30F How does **your** income compare to that of **men** in Stamola?

- I earn more than 25% of men (1)
- I earn more than 45% of men (2)
- I earn more than 65% of men (3)
- I earn more than 85% of men (4)
- I earn more than 99% of men (5)

Page Break

gap2M3F30F How **gender unequal** is income in Stamola?

- 1 Not at all gender unequal (1)
- 2 (2)
- 3 (3)
- 4 Moderately gender unequal (4)
- 5 (5)
- 6 (6)
- 7 Extremely gender unequal (7)

---

wwealth2M3F30F Compared to **women** in Stamola, how **wealthy** are **you**?

- 1 Extremely poor (1)
- 2 (2)
- 3 (3)
- 4 Neither poor nor wealthy (4)
- 5 (5)
- 6 (8)
- 7 Extremely wealthy (9)

---

mwealth2M3F30F Compared to **men** in Stamola, how **wealthy** are **you**?

- 1 Extremely poor (1)
- 2 (2)
- 3 (3)
- 4 Neither poor nor wealthy (4)
- 5 (5)
- 6 (8)
- 7 Extremely wealthy (9)

Page Break

Q1115

Q1167 **Think about your financial situation in Stamola.** Given how much money ${e://Field/opgenpeople} make, and how much money you make relative to others, **what would your romantic relationships with ${e://Field/opgenpeople} be like in Stamolean society?** Specifically, what would you want in a partner? **Reflect on these questions for 30 seconds, and record your thoughts using the text box below.** As you progress throughout the survey, answer any questions with these thoughts in mind. After 30 seconds, a button will appear in the lower right hand corner of the screen and you may proceed to the next part of the survey.

____

____

____

____

____

Q1211 Timing
First Click (1)
Last Click (2)
Page Submit (3)
Click Count (4)

End of Block: 2M:3F 30th Female (DC)

Start of Block: 2M:3F 10th Female (DC)

Q2378 Timing
First Click (1)
Last Click (2)
Page Submit (3)
Click Count (4)

instruct2M3F10F The video below will give you some information about the economy and your own financial situation in Stamola. Please watch the video carefully, because attention checks

are included on the following page. These attention checks will be used to confirm whether you've understood, and remembered, key details about the economy in Stamola and your own financial situation. A button will appear in the lower right hand corner of the screen once the video is finished, and you will be able to proceed to the next part of the survey.

---

video2M3F10F

Page Break

ratio2M3F10F Which of the following options best describes **the average gender gap** in Stamola**?**

- Men earn 2x more than women (1)
- Men earn 1.5x more than women (2)
- There is no difference between the average earnings of men and women (3)
- Women earn 1.5x more than men (4)
- Women earn 2x more than men (5)

---

wpercent2M3F10F How does **your** income compare to that of **women** in Stamola?

- I earn more than 10% of women (1)
- I earn more than 30% of women (2)
- I earn more than 50% of women (3)
- I earn more than 70% of women (4)
- I earn more than 90% of women (5)

---

mpercent2M3F10F How does **your** income compare to that of **men** in Stamola?

- I earn more than 25% of men (1)
- I earn more than 45% of men (2)
- I earn more than 65% of men (3)
- I earn more than 85% of men (4)
- I earn more than 99% of men (5)

Page Break

gap2M3F10F How **gender unequal** is income in Stamola?

- 1 Not at all gender unequal (1)
- 2 (2)
- 3 (3)
- 4 Moderately gender unequal (4)
- 5 (5)
- 6 (6)
- 7 Extremely gender unequal (7)

---

wwealth2M3F10F Compared to **women** in Stamola, how **wealthy** are **you**?

- 1 Extremely poor (1)
- 2 (2)
- 3 (3)
- 4 Neither poor nor wealthy (4)
- 5 (5)
- 6 (8)
- 7 Extremely wealthy (9)

---

mwealth2M3F10F Compared to **men** in Stamola, how **wealthy** are **you**?

- 1 Extremely poor (1)
- 2 (2)
- 3 (3)
- 4 Neither poor nor wealthy (4)
- 5 (5)
- 6 (8)
- 7 Extremely wealthy (9)

Page Break

Q1117

Q1168 **Think about your financial situation in Stamola.** Given how much money ${e://Field/opgenpeople} make, and how much money you make relative to others, **what would your romantic relationships with ${e://Field/opgenpeople} be like in Stamolean society?** Specifically, what would you want in a partner? **Reflect on these questions for 30 seconds, and record your thoughts using the text box below.** As you progress throughout the survey, answer any questions with these thoughts in mind. After 30 seconds, a button will appear in the lower right hand corner of the screen and you may proceed to the next part of the survey.

____

____

____

____

____

Q1212 Timing
First Click (1)
Last Click (2)
Page Submit (3)
Click Count (4)

End of Block: 2M:3F 10th Female (DC)

Start of Block: 1M:2F 90th Female (DC)

Q2432 Timing
First Click (1)
Last Click (2)
Page Submit (3)
Click Count (4)

instruct1M2F90F The video below will give you some information about the economy and your own financial situation in Stamola. Please watch the video carefully, because attention checks

are included on the following page. These attention checks will be used to confirm whether you've understood, and remembered, key details about the economy in Stamola and your own financial situation. A button will appear in the lower right hand corner of the screen once the video is finished, and you will be able to proceed to the next part of the survey.

video1M2F90F

Page Break

ratio1M2F90F Which of the following options best describes **the average gender gap** in Stamola**?**

- Men earn 2x more than women (1)
- Men earn 1.5x more than women (2)
- There is no difference between the average earnings of men and women (3)
- Women earn 1.5x more than men (4)
- Women earn 2x more than men (5)

---

wpercent1M2F90F How does **your** income compare to that of **women** in Stamola?

- I earn more than 10% of women (1)
- I earn more than 30% of women (2)
- I earn more than 50% of women (3)
- I earn more than 70% of women (4)
- I earn more than 90% of women (5)

---

mpercent1M2F90F How does **your** income compare to that of **men** in Stamola?

- ○ I earn more than 35% of men (1)
- ○ I earn more than 55% of men (2)
- ○ I earn more than 75% of men (3)
- ○ I earn more than 95% of men (4)
- ○ I earn more than 99% of men (5)

Page Break

gap1M2F90F How **gender unequal** is income in Stamola?

- 1 Not at all gender unequal (1)
- 2 (2)
- 3 (3)
- 4 Moderately gender unequal (4)
- 5 (5)
- 6 (6)
- 7 Extremely gender unequal (7)

---

wwealth1M2F90F Compared to **women** in Stamola, how **wealthy** are **you**?

- 1 Extremely poor (1)
- 2 (2)
- 3 (3)
- 4 Neither poor nor wealthy (4)
- 5 (5)
- 6 (8)
- 7 Extremely wealthy (9)

---

mwealth1M2F90F Compared to **men** in Stamola, how **wealthy** are **you**?

- 1 Extremely poor (1)
- 2 (2)
- 3 (3)
- 4 Neither poor nor wealthy (4)
- 5 (5)
- 6 (8)
- 7 Extremely wealthy (9)

Page Break

Q1119

Q1169 **Think about your financial situation in Stamola.** Given how much money ${e://Field/opgenpeople} make, and how much money you make relative to others, **what would your romantic relationships with ${e://Field/opgenpeople} be like in Stamolean society?** Specifically, what would you want in a partner? **Reflect on these questions for 30 seconds, and record your thoughts using the text box below.** As you progress throughout the survey, answer any questions with these thoughts in mind. After 30 seconds, a button will appear in the lower right hand corner of the screen and you may proceed to the next part of the survey.

____

____

____

____

____

Q1213 Timing
First Click (1)
Last Click (2)
Page Submit (3)
Click Count (4)

End of Block: 1M:2F 90th Female (DC)

Start of Block: 1M:2F 70th Female (DC)

Q2441 Timing
First Click (1)
Last Click (2)
Page Submit (3)
Click Count (4)

instruct1M2F70F The video below will give you some information about the economy and your own financial situation in Stamola. Please watch the video carefully, because attention checks

are included on the following page. These attention checks will be used to confirm whether you've understood, and remembered, key details about the economy in Stamola and your own financial situation. A button will appear in the lower right hand corner of the screen once the video is finished, and you will be able to proceed to the next part of the survey.

video1M2F70F

Page Break

ratio1M2F70F Which of the following options best describes **the average gender gap** in Stamola**?**

- Men earn 2x more than women (1)
- Men earn 1.5x more than women (2)
- There is no difference between the average earnings of men and women (3)
- Women earn 1.5x more than men (4)
- Women earn 2x more than men (5)

---

wpercent1M2F70F How does **your** income compare to that of **women** in Stamola?

- I earn more than 10% of women (1)
- I earn more than 30% of women (2)
- I earn more than 50% of women (3)
- I earn more than 70% of women (4)
- I earn more than 90% of women (5)

---

mpercent1M2F70F How does **your** income compare to that of **men** in Stamola?

- I earn more than 35% of men (1)
- I earn more than 55% of men (2)
- I earn more than 75% of men (3)
- I earn more than 95% of men (4)
- I earn more than 99% of men (5)

Page Break

gap1M2F70F How **gender unequal** is income in Stamola?

- 1 Not at all gender unequal (1)
- 2 (2)
- 3 (3)
- 4 Moderately gender unequal (4)
- 5 (5)
- 6 (6)
- 7 Extremely gender unequal (7)

---

wwealth1M2F70F Compared to **women** in Stamola, how **wealthy** are **you**?

- 1 Extremely poor (1)
- 2 (2)
- 3 (3)
- 4 Neither poor nor wealthy (4)
- 5 (5)
- 6 (8)
- 7 Extremely wealthy (9)

---

mwealth1M2F70F Compared to **men** in Stamola, how **wealthy** are **you**?

○ 1 Extremely poor (1)

○ 2 (2)

○ 3 (3)

○ 4 Neither poor nor wealthy (4)

○ 5 (5)

○ 6 (8)

○ 7 Extremely wealthy (9)

Page Break

Q1121

Q1170 **Think about your financial situation in Stamola.** Given how much money ${e://Field/opgenpeople} make, and how much money you make relative to others, **what would your romantic relationships with ${e://Field/opgenpeople} be like in Stamolean society?** Specifically, what would you want in a partner? **Reflect on these questions for 30 seconds, and record your thoughts using the text box below.** As you progress throughout the survey, answer any questions with these thoughts in mind. After 30 seconds, a button will appear in the lower right hand corner of the screen and you may proceed to the next part of the survey.

____

____

____

____

____

Q1214 Timing
First Click (1)
Last Click (2)
Page Submit (3)
Click Count (4)

End of Block: 1M:2F 70th Female (DC)

Start of Block: 1M:2F 50th Female (DC)

Q2450 Timing
First Click (1)
Last Click (2)
Page Submit (3)
Click Count (4)

instruct1M2F50F The video below will give you some information about the economy and your own financial situation in Stamola. Please watch the video carefully, because attention checks

are included on the following page. These attention checks will be used to confirm whether you've understood, and remembered, key details about the economy in Stamola and your own financial situation. A button will appear in the lower right hand corner of the screen once the video is finished, and you will be able to proceed to the next part of the survey.

video1M2F50F

Page Break

ratio1M2F50F Which of the following options best describes **the average gender gap** in Stamola**?**

- ○ Men earn 2x more than women (1)
- ○ Men earn 1.5x more than women (2)
- ○ There is no difference between the average earnings of men and women (3)
- ○ Women earn 1.5x more than men (4)
- ○ Women earn 2x more than men (5)

---

wpercent1M2F50F How does **your** income compare to that of **women** in Stamola?

- ○ I earn more than 10% of women (1)
- ○ I earn more than 30% of women (2)
- ○ I earn more than 50% of women (3)
- ○ I earn more than 70% of women (4)
- ○ I earn more than 90% of women (5)

---

mpercent1M2F50F How does **your** income compare to that of **men** in Stamola?

- I earn more than 35% of men (1)
- I earn more than 55% of men (2)
- I earn more than 75% of men (3)
- I earn more than 95% of men (4)
- I earn more than 99% of men (5)

Page Break

gap1M2F50F How **gender unequal** is income in Stamola?

- 1 Not at all gender unequal (1)
- 2 (2)
- 3 (3)
- 4 Moderately gender unequal (4)
- 5 (5)
- 6 (6)
- 7 Extremely gender unequal (7)

---

wwealth1M2F50F Compared to **women** in Stamola, how **wealthy** are **you**?

- 1 Extremely poor (1)
- 2 (2)
- 3 (3)
- 4 Neither poor nor wealthy (4)
- 5 (5)
- 6 (8)
- 7 Extremely wealthy (9)

---

mwealth1M2F50F Compared to **men** in Stamola, how **wealthy** are **you**?

- 1 Extremely poor (1)
- 2 (2)
- 3 (3)
- 4 Neither poor nor wealthy (4)
- 5 (5)
- 6 (8)
- 7 Extremely wealthy (9)

Page Break

Q1123

Q1171 **Think about your financial situation in Stamola.** Given how much money ${e://Field/opgenpeople} make, and how much money you make relative to others, **what would your romantic relationships with ${e://Field/opgenpeople} be like in Stamolean society?** Specifically, what would you want in a partner? **Reflect on these questions for 30 seconds, and record your thoughts using the text box below.** As you progress throughout the survey, answer any questions with these thoughts in mind. After 30 seconds, a button will appear in the lower right hand corner of the screen and you may proceed to the next part of the survey.

____

____

____

____

____

Q1215 Timing
First Click (1)
Last Click (2)
Page Submit (3)
Click Count (4)

End of Block: 1M:2F 50th Female (DC)

Start of Block: 1M:2F 30th Female (DC)

Q2459 Timing
First Click (1)
Last Click (2)
Page Submit (3)
Click Count (4)

instruct1M2F30F The video below will give you some information about the economy and your own financial situation in Stamola. Please watch the video carefully, because attention checks

are included on the following page. These attention checks will be used to confirm whether you've understood, and remembered, key details about the economy in Stamola and your own financial situation. A button will appear in the lower right hand corner of the screen once the video is finished, and you will be able to proceed to the next part of the survey.

---

video1M2F30F

Page Break

ratio1M2F30F Which of the following options best describes **the average gender gap** in Stamola**?**

- Men earn 2x more than women (1)
- Men earn 1.5x more than women (2)
- There is no difference between the average earnings of men and women (3)
- Women earn 1.5x more than men (4)
- Women earn 2x more than men (5)

---

wpercent1M2F30F How does **your** income compare to that of **women** in Stamola?

- I earn more than 10% of women (1)
- I earn more than 30% of women (2)
- I earn more than 50% of women (3)
- I earn more than 70% of women (4)
- I earn more than 90% of women (5)

---

mpercent1M2F30F How does **your** income compare to that of **men** in Stamola?

- ○ I earn more than 35% of men (1)
- ○ I earn more than 55% of men (2)
- ○ I earn more than 75% of men (3)
- ○ I earn more than 95% of men (4)
- ○ I earn more than 99% of men (5)

Page Break

gap1M2F30F How **gender unequal** is income in Stamola?

- 1 Not at all gender unequal (1)
- 2 (2)
- 3 (3)
- 4 Moderately gender unequal (4)
- 5 (5)
- 6 (6)
- 7 Extremely gender unequal (7)

---

wwealth1M2F30F Compared to **women** in Stamola, how **wealthy** are **you**?

- 1 Extremely poor (1)
- 2 (2)
- 3 (3)
- 4 Neither poor nor wealthy (4)
- 5 (5)
- 6 (8)
- 7 Extremely wealthy (9)

---

mwealth1M2F30F Compared to **men** in Stamola, how **wealthy** are **you**?

- 1 Extremely poor (1)
- 2 (2)
- 3 (3)
- 4 Neither poor nor wealthy (4)
- 5 (5)
- 6 (8)
- 7 Extremely wealthy (9)

Page Break

Q1125

Q1172 **Think about your financial situation in Stamola.** Given how much money ${e://Field/opgenpeople} make, and how much money you make relative to others, **what would your romantic relationships with ${e://Field/opgenpeople} be like in Stamolean society?** Specifically, what would you want in a partner? **Reflect on these questions for 30 seconds, and record your thoughts using the text box below.** As you progress throughout the survey, answer any questions with these thoughts in mind. After 30 seconds, a button will appear in the lower right hand corner of the screen and you may proceed to the next part of the survey.

____

____

____

____

____

Q1216 Timing
First Click (1)
Last Click (2)
Page Submit (3)
Click Count (4)

End of Block: 1M:2F 30th Female (DC)

Start of Block: 1M:2F 10th Female (DC)

Q2468 Timing
First Click (1)
Last Click (2)
Page Submit (3)
Click Count (4)

---

instruct1M2F10F The video below will give you some information about the economy and your own financial situation in Stamola. Please watch the video carefully, because attention checks

are included on the following page. These attention checks will be used to confirm whether you've understood, and remembered, key details about the economy in Stamola and your own financial situation. A button will appear in the lower right hand corner of the screen once the video is finished, and you will be able to proceed to the next part of the survey.

video1M2F10F

Page Break

ratio1M2F10F Which of the following options best describes **the average gender gap** in Stamola**?**

- Men earn 2x more than women (1)
- Men earn 1.5x more than women (2)
- There is no difference between the average earnings of men and women (3)
- Women earn 1.5x more than men (4)
- Women earn 2x more than men (5)

---

wpercent1M2F10F How does **your** income compare to that of **women** in Stamola?

- I earn more than 10% of women (1)
- I earn more than 30% of women (2)
- I earn more than 50% of women (3)
- I earn more than 70% of women (4)
- I earn more than 90% of women (5)

---

mpercent1M2F10F How does **your** income compare to that of **men** in Stamola?

- I earn more than 35% of men (1)
- I earn more than 55% of men (2)
- I earn more than 75% of men (3)
- I earn more than 95% of men (4)
- I earn more than 99% of men (5)

Page Break

gap1M2F10F How **gender unequal** is income in Stamola?

- 1 Not at all gender unequal (1)
- 2 (2)
- 3 (3)
- 4 Moderately gender unequal (4)
- 5 (5)
- 6 (6)
- 7 Extremely gender unequal (7)

---

wwealth1M2F10F Compared to **women** in Stamola, how **wealthy** are **you**?

- 1 Extremely poor (1)
- 2 (2)
- 3 (3)
- 4 Neither poor nor wealthy (4)
- 5 (5)
- 6 (8)
- 7 Extremely wealthy (9)

---

mwealth1M2F10F Compared to **men** in Stamola, how **wealthy** are **you**?

- 1 Extremely poor (1)
- 2 (2)
- 3 (3)
- 4 Neither poor nor wealthy (4)
- 5 (5)
- 6 (8)
- 7 Extremely wealthy (9)

Page Break

Q1127

Q1173 **Think about your financial situation in Stamola.** Given how much money ${e://Field/opgenpeople} make, and how much money you make relative to others, **what would your romantic relationships with ${e://Field/opgenpeople} be like in Stamolean society?** Specifically, what would you want in a partner? **Reflect on these questions for 30 seconds, and record your thoughts using the text box below.** As you progress throughout the survey, answer any questions with these thoughts in mind. After 30 seconds, a button will appear in the lower right hand corner of the screen and you may proceed to the next part of the survey.

____

____

____

____

____

Q1217 Timing
First Click (1)
Last Click (2)
Page Submit (3)
Click Count (4)

End of Block: 1M:2F 10th Female (DC)

Start of Block: Manipulation Strengthener

Q930 **When people join Stamola they are single, but can meet people and enter a long-term relationship. Because you have just joined Stamola, you are currently single.** In the remainder of the study, we will ask about your romantic preferences when choosing a ${e://Field/opgenperson} to be ${e://Field/ltr} in Stamola.

End of Block: Manipulation Strengthener

Start of Block: Mate Preferences 1

X→ X→

Hypergamy Some ${e://Field/samegenpeople} desire a romantic partner who makes more money than them or has more money than them. **When choosing a long-term partner** (e.g., ${e://Field/ltr}) **in Stamola, how important would it be for a ${e://Field/opgenperson} to be better off than you financially?**

- 0 **Not at all important** (1)
- 1 (2)
- 2 (3)
- 3 (4)
- 4 (5)
- 5 **Moderately important** (6)
- 6 (7)
- 7 (8)
- 8 (9)
- 9 (10)
- 10 **Extremely important** (11)

Page Break

AgeGap Some ${e://Field/samegenpeople} desire a romantic partner older than them, some ${e://Field/samegenpeople} desire a romantic partner younger than them, and some ${e://Field/samegenpeople} desire a romantic partner the same age as themselves. **Using the box below, please carefully select what would be the ideal age difference between you and a long-term partner** (e.g., ${e://Field/ltr}) **in Stamola.** ***When choosing a long-term partner in Stamola, if I had to choose, I would prefer a ${e://Field/opgenperson}...***

▼ 30 OR MORE years YOUNGER than me. (-30) ... 30 OR MORE years OLDER than me. (30)

Page Break

EastwickRating **Please rate the extent to which the following attributes would be desirable to you when choosing a long-term partner** (e.g., ${e://Field/ltr}) **in Stamola.**

	0 (1)	1 (2)	2 (3)	3 (4)	4 (5)	5 (6)	6 (7)	7 (8)	8 (9)	9 (10)	10 (11)
Attractive (Attractive)	○	○	○	○	○	○	○	○	○	○	○
Sporty and athletic (Sporty and athletic)	○	○	○	○	○	○	○	○	○	○	○
Financially secure (Financially secure)	○	○	○	○	○	○	○	○	○	○	○
Fun (Fun)	○	○	○	○	○	○	○	○	○	○	○
Open to new experiences, complex (Open to new experiences, complex)	○	○	○	○	○	○	○	○	○	○	○
Intelligent (Intelligent)	○	○	○	○	○	○	○	○	○	○	○
Good job (Good job)	○	○	○	○	○	○	○	○	○	○	○
Supportive (Supportive)	○	○	○	○	○	○	○	○	○	○	○
Religious (Religious)	○	○	○	○	○	○	○	○	○	○	○
Reserved, quiet (Reserved, quiet)	○	○	○	○	○	○	○	○	○	○	○
Ambitious (Ambitious)	○	○	○	○	○	○	○	○	○	○	○
Sexy (Sexy)	○	○	○	○	○	○	○	○	○	○	○

Adventurous (Adventurous)											
Anxious, easily upset (Anxious, easily upset)											
Smells good (Smells good)											
Understanding (Understanding)											
Confident (Confident)											
Successful (Successful)											
Dependable, self-disciplined (Dependable, self-disciplined)											
Conventional, uncreative (Conventional, uncreative)											
Honest (Honest)											
Nice body (Nice body)											
Loyal (Loyal)											
Critical, quarrelsome (Critical, quarrelsome)											
Calm, emotionally stable (Calm, emotionally stable)											

Considerate (Considerate)	○	○	○	○	○	○	○	○	○	○	○
A good lover (A good lover)	○	○	○	○	○	○	○	○	○	○	○
A good listener (A good listener)	○	○	○	○	○	○	○	○	○	○	○
Extraverted, enthusiastic (Extraverted, enthusiastic)	○	○	○	○	○	○	○	○	○	○	○
Disorganized, careless (Disorganized, careless)	○	○	○	○	○	○	○	○	○	○	○
Humorous (Humorous)	○	○	○	○	○	○	○	○	○	○	○
Sensitive (Sensitive)	○	○	○	○	○	○	○	○	○	○	○
Dresses well (Dresses well)	○	○	○	○	○	○	○	○	○	○	○
Patient (Patient)	○	○	○	○	○	○	○	○	○	○	○
Sympathetic, warm (Sympathetic, warm)	○	○	○	○	○	○	○	○	○	○	○

End of Block: Mate Preferences 1

Start of Block: 2M:1F 90th Male - Manipulation Reminder

2M:1F 90th Male Rem

Q2544 Think about how much you earn in Stamola compared to others again. Remember how your income compares to women and men.   Spend 15 seconds remembering what life in Stamola would be like if you earnt this much in Stamolean society.   How might it shape your romantic relationships with ${e://Field/opgenpeople}? How might it influence what you want in a partner?   As you progress throughout the survey, answer any questions with these thoughts in mind.   After 15 seconds, a button will appear in the lower right hand corner of the screen and you may proceed to the next part of the survey.

Q2545 Timing
First Click (1)
Last Click (2)
Page Submit (3)
Click Count (4)

End of Block: 2M:1F 90th Male - Manipulation Reminder

Start of Block: 2M:1F 90th Female - Manipulation Reminder

2M:1F 90th F Rem

Q2621 Think about how much you earn in Stamola compared to others again. Remember how your income compares to women and men.   Spend 15 seconds remembering what life in Stamola would be like if you earnt this much in Stamolean society.   How might it shape your romantic relationships with ${e://Field/opgenpeople}? How might it influence what you want in a partner?   As you progress throughout the survey, answer any questions with these thoughts in mind.   After 15 seconds, a button will appear in the lower right hand corner of the screen and you may proceed to the next part of the survey.

Q2622 Timing
First Click (1)
Last Click (2)
Page Submit (3)
Click Count (4)

End of Block: 2M:1F 90th Female - Manipulation Reminder

Start of Block: 2M:1F 70th Male - Manipulation Reminder

2M:1F 70th Male Rem

Q2549 Think about how much you earn in Stamola compared to others again. Remember how your income compares to women and men.   Spend 15 seconds remembering what life in Stamola would be like if you earnt this much in Stamolean society.   How might it shape your romantic relationships with ${e://Field/opgenpeople}? How might it influence what you want in a partner?   As you progress throughout the survey, answer any questions with these thoughts in mind.   After 15 seconds, a button will appear in the lower right hand corner of the screen and you may proceed to the next part of the survey.

Q2550 Timing
First Click (1)
Last Click (2)
Page Submit (3)
Click Count (4)

End of Block: 2M:1F 70th Male - Manipulation Reminder

Start of Block: 2M:1F 70th Female - Manipulation Reminder

2M:1F 70th F Rem

Q2624 Think about how much you earn in Stamola compared to others again. Remember how your income compares to women and men.   Spend 15 seconds remembering what life in Stamola would be like if you earnt this much in Stamolean society.   How might it shape your romantic relationships with ${e://Field/opgenpeople}? How might it influence what you want in a partner?   As you progress throughout the survey, answer any questions with these thoughts in mind.   After 15 seconds, a button will appear in the lower right hand corner of the screen and you may proceed to the next part of the survey.

Q2625 Timing
First Click (1)
Last Click (2)
Page Submit (3)
Click Count (4)

End of Block: 2M:1F 70th Female - Manipulation Reminder

Start of Block: 2M:1F 50th Male - Manipulation Reminder

2M:1F 50th Male Rem

Q2552 Think about how much you earn in Stamola compared to others again. Remember how your income compares to women and men. Spend 15 seconds remembering what life in Stamola would be like if you earnt this much in Stamolean society. How might it shape your romantic relationships with ${e://Field/opgenpeople}? How might it influence what you want in a partner? As you progress throughout the survey, answer any questions with these thoughts in mind. After 15 seconds, a button will appear in the lower right hand corner of the screen and you may proceed to the next part of the survey.

Q2553 Timing
First Click (1)
Last Click (2)
Page Submit (3)
Click Count (4)

End of Block: 2M:1F 50th Male - Manipulation Reminder

Start of Block: 2M:1F 50th Female - Manipulation Reminder

2M:1F 50th F Rem

Q2627 Think about how much you earn in Stamola compared to others again. Remember how your income compares to women and men. Spend 15 seconds remembering what life in Stamola would be like if you earnt this much in Stamolean society. How might it shape your romantic relationships with ${e://Field/opgenpeople}? How might it influence what you want in a partner? As you progress throughout the survey, answer any questions with these thoughts in

mind. After 15 seconds, a button will appear in the lower right hand corner of the screen and you may proceed to the next part of the survey.

Q2628 Timing
First Click (1)
Last Click (2)
Page Submit (3)
Click Count (4)

End of Block: 2M:1F 50th Female - Manipulation Reminder

Start of Block: 2M:1F 30th Male - Manipulation Reminder

2M1F 30th Male Rem

Q2555 Think about how much you earn in Stamola compared to others again. Remember how your income compares to women and men. Spend 15 seconds remembering what life in Stamola would be like if you earnt this much in Stamolean society. How might it shape your romantic relationships with ${e://Field/opgenpeople}? How might it influence what you want in a partner? As you progress throughout the survey, answer any questions with these thoughts in mind. After 15 seconds, a button will appear in the lower right hand corner of the screen and you may proceed to the next part of the survey.

Q2556 Timing
First Click (1)
Last Click (2)
Page Submit (3)
Click Count (4)

End of Block: 2M:1F 30th Male - Manipulation Reminder

Start of Block: 2M:1F 30th Female - Manipulation Reminder

2M:1F 30th F Rem

Q2630 Think about how much you earn in Stamola compared to others again. Remember how your income compares to women and men.   Spend 15 seconds remembering what life in Stamola would be like if you earnt this much in Stamolean society.   How might it shape your romantic relationships with ${e://Field/opgenpeople}? How might it influence what you want in a partner?   As you progress throughout the survey, answer any questions with these thoughts in mind.   After 15 seconds, a button will appear in the lower right hand corner of the screen and you may proceed to the next part of the survey.

Q2631 Timing
First Click (1)
Last Click (2)
Page Submit (3)
Click Count (4)

End of Block: 2M:1F 30th Female - Manipulation Reminder

Start of Block: 2M:1F 10th Male - Manipulation Reminder

2M:1F 10th Male Rem

Q2558 Think about how much you earn in Stamola compared to others again. Remember how your income compares to women and men.   Spend 15 seconds remembering what life in Stamola would be like if you earnt this much in Stamolean society.   How might it shape your romantic relationships with ${e://Field/opgenpeople}? How might it influence what you want in a partner?   As you progress throughout the survey, answer any questions with these thoughts in mind.   After 15 seconds, a button will appear in the lower right hand corner of the screen and you may proceed to the next part of the survey.

Q2559 Timing
First Click (1)
Last Click (2)
Page Submit (3)
Click Count (4)

End of Block: 2M:1F 10th Male - Manipulation Reminder

Start of Block: 2M:1F 10th Female - Manipulation Reminder

2M:1F 10th F Rem

Q2633 Think about how much you earn in Stamola compared to others again. Remember how your income compares to women and men.   Spend 15 seconds remembering what life in Stamola would be like if you earnt this much in Stamolean society.   How might it shape your romantic relationships with ${e://Field/opgenpeople}? How might it influence what you want in a partner?   As you progress throughout the survey, answer any questions with these thoughts in mind.   After 15 seconds, a button will appear in the lower right hand corner of the screen and you may proceed to the next part of the survey.

Q2634 Timing
First Click (1)
Last Click (2)
Page Submit (3)
Click Count (4)

End of Block: 2M:1F 10th Female - Manipulation Reminder

Start of Block: 3M:2F 90th Male - Manipulation Reminder

3M:2F 90th Male Rem

Q2561 Think about how much you earn in Stamola compared to others again. Remember how your income compares to women and men.   Spend 15 seconds remembering what life in Stamola would be like if you earnt this much in Stamolean society.   How might it shape your romantic relationships with ${e://Field/opgenpeople}? How might it influence what you want in a partner?   As you progress throughout the survey, answer any questions with these thoughts in mind.   After 15 seconds, a button will appear in the lower right hand corner of the screen and you may proceed to the next part of the survey.

Q2562 Timing
First Click (1)
Last Click (2)
Page Submit (3)
Click Count (4)

End of Block: 3M:2F 90th Male - Manipulation Reminder

Start of Block: 3M:2F 90th Female - Manipulation Reminder

3M:2F 90th F Rem

Q2636 Think about how much you earn in Stamola compared to others again. Remember how your income compares to women and men. Spend 15 seconds remembering what life in Stamola would be like if you earnt this much in Stamolean society. How might it shape your romantic relationships with ${e://Field/opgenpeople}? How might it influence what you want in a partner? As you progress throughout the survey, answer any questions with these thoughts in mind. After 15 seconds, a button will appear in the lower right hand corner of the screen and you may proceed to the next part of the survey.

Q2637 Timing
First Click (1)
Last Click (2)
Page Submit (3)
Click Count (4)

End of Block: 3M:2F 90th Female - Manipulation Reminder

Start of Block: 3M:2F 70th Male - Manipulation Reminder

3M:2F 70th Male Rem

Q2573 Think about how much you earn in Stamola compared to others again. Remember how your income compares to women and men. Spend 15 seconds remembering what life in Stamola would be like if you earnt this much in Stamolean society. How might it shape your romantic relationships with ${e://Field/opgenpeople}? How might it influence what you want in a partner? As you progress throughout the survey, answer any questions with these thoughts in

mind.   After 15 seconds, a button will appear in the lower right hand corner of the screen and you may proceed to the next part of the survey.

Q2574 Timing
First Click (1)
Last Click (2)
Page Submit (3)
Click Count (4)

End of Block: 3M:2F 70th Male - Manipulation Reminder

Start of Block: 3M:2F 70th Female - Manipulation Reminder

3M:2F 70th F Rem

Q2639 Think about how much you earn in Stamola compared to others again. Remember how your income compares to women and men.   Spend 15 seconds remembering what life in Stamola would be like if you earnt this much in Stamolean society.   How might it shape your romantic relationships with ${e://Field/opgenpeople}? How might it influence what you want in a partner?   As you progress throughout the survey, answer any questions with these thoughts in mind.   After 15 seconds, a button will appear in the lower right hand corner of the screen and you may proceed to the next part of the survey.

Q2640 Timing
First Click (1)
Last Click (2)
Page Submit (3)
Click Count (4)

End of Block: 3M:2F 70th Female - Manipulation Reminder

Start of Block: 3M:2F 50th Male - Manipulation Reminder

3M:2F 50th Male Rem

Q2564 Think about how much you earn in Stamola compared to others again. Remember how your income compares to women and men. Spend 15 seconds remembering what life in Stamola would be like if you earnt this much in Stamolean society. How might it shape your romantic relationships with ${e://Field/opgenpeople}? How might it influence what you want in a partner? As you progress throughout the survey, answer any questions with these thoughts in mind. After 15 seconds, a button will appear in the lower right hand corner of the screen and you may proceed to the next part of the survey.

Q2565 Timing
First Click (1)
Last Click (2)
Page Submit (3)
Click Count (4)

End of Block: 3M:2F 50th Male - Manipulation Reminder

Start of Block: 3M:2F 50th Female - Manipulation Reminder

3M:2F 50th F Rem

Q2642 Think about how much you earn in Stamola compared to others again. Remember how your income compares to women and men. Spend 15 seconds remembering what life in Stamola would be like if you earnt this much in Stamolean society. How might it shape your romantic relationships with ${e://Field/opgenpeople}? How might it influence what you want in a partner? As you progress throughout the survey, answer any questions with these thoughts in mind. After 15 seconds, a button will appear in the lower right hand corner of the screen and you may proceed to the next part of the survey.

Q2643 Timing
First Click (1)
Last Click (2)
Page Submit (3)
Click Count (4)

End of Block: 3M:2F 50th Female - Manipulation Reminder

Start of Block: 3M:2F 30th Male - Manipulation Reminder

3M:2F 30th Male Rem

Q2567 Think about how much you earn in Stamola compared to others again. Remember how your income compares to women and men.   Spend 15 seconds remembering what life in Stamola would be like if you earnt this much in Stamolean society.   How might it shape your romantic relationships with ${e://Field/opgenpeople}? How might it influence what you want in a partner?   As you progress throughout the survey, answer any questions with these thoughts in mind.   After 15 seconds, a button will appear in the lower right hand corner of the screen and you may proceed to the next part of the survey.

Q2568 Timing
First Click (1)
Last Click (2)
Page Submit (3)
Click Count (4)

End of Block: 3M:2F 30th Male - Manipulation Reminder

Start of Block: 3M:2F 30th Female - Manipulation Reminder

3M:2F 30th F Rem

Q2645 Think about how much you earn in Stamola compared to others again. Remember how your income compares to women and men.   Spend 15 seconds remembering what life in Stamola would be like if you earnt this much in Stamolean society.   How might it shape your romantic relationships with ${e://Field/opgenpeople}? How might it influence what you want in a partner?   As you progress throughout the survey, answer any questions with these thoughts in mind.   After 15 seconds, a button will appear in the lower right hand corner of the screen and you may proceed to the next part of the survey.

Q2646 Timing
First Click (1)
Last Click (2)
Page Submit (3)
Click Count (4)

End of Block: 3M:2F 30th Female - Manipulation Reminder

Start of Block: 3M:2F 10th Male - Manipulation Reminder

3M:2F 10th Male Rem

Q2570 Think about how much you earn in Stamola compared to others again. Remember how your income compares to women and men. Spend 15 seconds remembering what life in Stamola would be like if you earnt this much in Stamolean society. How might it shape your romantic relationships with ${e://Field/opgenpeople}? How might it influence what you want in a partner? As you progress throughout the survey, answer any questions with these thoughts in mind. After 15 seconds, a button will appear in the lower right hand corner of the screen and you may proceed to the next part of the survey.

Q2571 Timing
First Click (1)
Last Click (2)
Page Submit (3)
Click Count (4)

End of Block: 3M:2F 10th Male - Manipulation Reminder

Start of Block: 3M:2F 10th Female - Manipulation Reminder

3M:2F 10th F Rem

Q2648 Think about how much you earn in Stamola compared to others again. Remember how your income compares to women and men. Spend 15 seconds remembering what life in Stamola would be like if you earnt this much in Stamolean society. How might it shape your romantic relationships with ${e://Field/opgenpeople}? How might it influence what you want in a partner? As you progress throughout the survey, answer any questions with these thoughts in

mind. After 15 seconds, a button will appear in the lower right hand corner of the screen and you may proceed to the next part of the survey.

Q2649 Timing
First Click (1)
Last Click (2)
Page Submit (3)
Click Count (4)

End of Block: 3M:2F 10th Female - Manipulation Reminder

Start of Block: 1M:1F 90th - Manipulation Reminder

1M:1F 90th Rem

Q2576 Think about how much you earn in Stamola compared to others again. Remember how your income compares to women and men. Spend 15 seconds remembering what life in Stamola would be like if you earnt this much in Stamolean society. How might it shape your romantic relationships with ${e://Field/opgenpeople}? How might it influence what you want in a partner? As you progress throughout the survey, answer any questions with these thoughts in mind. After 15 seconds, a button will appear in the lower right hand corner of the screen and you may proceed to the next part of the survey.

Q2577 Timing
First Click (1)
Last Click (2)
Page Submit (3)
Click Count (4)

End of Block: 1M:1F 90th - Manipulation Reminder

Start of Block: 1M:1F 70th - Manipulation Reminder

1M:1F 70th Rem

Q2579 Think about how much you earn in Stamola compared to others again. Remember how your income compares to women and men.   Spend 15 seconds remembering what life in Stamola would be like if you earnt this much in Stamolean society.   How might it shape your romantic relationships with ${e://Field/opgenpeople}? How might it influence what you want in a partner?   As you progress throughout the survey, answer any questions with these thoughts in mind.   After 15 seconds, a button will appear in the lower right hand corner of the screen and you may proceed to the next part of the survey.

Q2580 Timing
First Click (1)
Last Click (2)
Page Submit (3)
Click Count (4)

End of Block: 1M:1F 70th - Manipulation Reminder

Start of Block: 1M:1F 50th - Manipulation Reminder

1M:1F 50th Rem

Q2582 Think about how much you earn in Stamola compared to others again. Remember how your income compares to women and men.   Spend 15 seconds remembering what life in Stamola would be like if you earnt this much in Stamolean society.   How might it shape your romantic relationships with ${e://Field/opgenpeople}? How might it influence what you want in a partner?   As you progress throughout the survey, answer any questions with these thoughts in mind.   After 15 seconds, a button will appear in the lower right hand corner of the screen and you may proceed to the next part of the survey.

Q2583 Timing
First Click (1)
Last Click (2)
Page Submit (3)
Click Count (4)

End of Block: 1M:1F 50th - Manipulation Reminder

Start of Block: 1M:1F 30th - Manipulation Reminder

1M:1F 30th Rem

Q2585 Think about how much you earn in Stamola compared to others again. Remember how your income compares to women and men.   Spend 15 seconds remembering what life in Stamola would be like if you earnt this much in Stamolean society.   How might it shape your romantic relationships with ${e://Field/opgenpeople}? How might it influence what you want in a partner?   As you progress throughout the survey, answer any questions with these thoughts in mind.   After 15 seconds, a button will appear in the lower right hand corner of the screen and you may proceed to the next part of the survey.

Q2586 Timing
First Click (1)
Last Click (2)
Page Submit (3)
Click Count (4)

End of Block: 1M:1F 30th - Manipulation Reminder

Start of Block: 1M:1F 10th - Manipulation Reminder

1M:1F 10th Rem

Q2588 Think about how much you earn in Stamola compared to others again. Remember how your income compares to women and men.   Spend 15 seconds remembering what life in Stamola would be like if you earnt this much in Stamolean society.   How might it shape your romantic relationships with ${e://Field/opgenpeople}? How might it influence what you want in a partner?   As you progress throughout the survey, answer any questions with these thoughts in mind.   After 15 seconds, a button will appear in the lower right hand corner of the screen and you may proceed to the next part of the survey.

Q2589 Timing
First Click (1)
Last Click (2)
Page Submit (3)
Click Count (4)

End of Block: 1M:1F 10th - Manipulation Reminder

Start of Block: 2M:3F 90th Male - Manipulation Reminder

2M:3F 90th Male Rem

Q2591 Think about how much you earn in Stamola compared to others again. Remember how your income compares to women and men. Spend 15 seconds remembering what life in Stamola would be like if you earnt this much in Stamolean society. How might it shape your romantic relationships with ${e://Field/opgenpeople}? How might it influence what you want in a partner? As you progress throughout the survey, answer any questions with these thoughts in mind. After 15 seconds, a button will appear in the lower right hand corner of the screen and you may proceed to the next part of the survey.

Q2592 Timing
First Click (1)
Last Click (2)
Page Submit (3)
Click Count (4)

End of Block: 2M:3F 90th Male - Manipulation Reminder

Start of Block: 2M:3F 90th Female - Manipulation Reminder

2M:3F 90th F Rem

Q2651 Think about how much you earn in Stamola compared to others again. Remember how your income compares to women and men. Spend 15 seconds remembering what life in Stamola would be like if you earnt this much in Stamolean society. How might it shape your romantic relationships with ${e://Field/opgenpeople}? How might it influence what you want in a partner? As you progress throughout the survey, answer any questions with these thoughts in

mind.   After 15 seconds, a button will appear in the lower right hand corner of the screen and you may proceed to the next part of the survey.

Q2652 Timing
First Click (1)
Last Click (2)
Page Submit (3)
Click Count (4)

End of Block: 2M:3F 90th Female - Manipulation Reminder

Start of Block: 2M:3F 70th Male - Manipulation Reminder

2M:3F 70th Male Rem

Q2594 Think about how much you earn in Stamola compared to others again. Remember how your income compares to women and men.   Spend 15 seconds remembering what life in Stamola would be like if you earnt this much in Stamolean society.   How might it shape your romantic relationships with ${e://Field/opgenpeople}? How might it influence what you want in a partner?   As you progress throughout the survey, answer any questions with these thoughts in mind.   After 15 seconds, a button will appear in the lower right hand corner of the screen and you may proceed to the next part of the survey.

Q2595 Timing
First Click (1)
Last Click (2)
Page Submit (3)
Click Count (4)

End of Block: 2M:3F 70th Male - Manipulation Reminder

Start of Block: 2M:3F 70th Female - Manipulation Reminder

2M:3F 70th F Rem

Q2654 Think about how much you earn in Stamola compared to others again. Remember how your income compares to women and men.   Spend 15 seconds remembering what life in Stamola would be like if you earnt this much in Stamolean society.   How might it shape your romantic relationships with ${e://Field/opgenpeople}? How might it influence what you want in a partner?   As you progress throughout the survey, answer any questions with these thoughts in mind.   After 15 seconds, a button will appear in the lower right hand corner of the screen and you may proceed to the next part of the survey.

Q2655 Timing
First Click (1)
Last Click (2)
Page Submit (3)
Click Count (4)

End of Block: 2M:3F 70th Female - Manipulation Reminder

Start of Block: 2M:3F 50th Male - Manipulation Reminder

2M:3F 50th Male Rem

Q2597 Think about how much you earn in Stamola compared to others again. Remember how your income compares to women and men.   Spend 15 seconds remembering what life in Stamola would be like if you earnt this much in Stamolean society.   How might it shape your romantic relationships with ${e://Field/opgenpeople}? How might it influence what you want in a partner?   As you progress throughout the survey, answer any questions with these thoughts in mind.   After 15 seconds, a button will appear in the lower right hand corner of the screen and you may proceed to the next part of the survey.

Q2598 Timing
First Click (1)
Last Click (2)
Page Submit (3)
Click Count (4)

End of Block: 2M:3F 50th Male - Manipulation Reminder

Start of Block: 2M:3F 50th Female - Manipulation Reminder

2M:3F 50th F Rem

Q2657 Think about how much you earn in Stamola compared to others again. Remember how your income compares to women and men. Spend 15 seconds remembering what life in Stamola would be like if you earnt this much in Stamolean society. How might it shape your romantic relationships with ${e://Field/opgenpeople}? How might it influence what you want in a partner? As you progress throughout the survey, answer any questions with these thoughts in mind. After 15 seconds, a button will appear in the lower right hand corner of the screen and you may proceed to the next part of the survey.

Q2658 Timing
First Click (1)
Last Click (2)
Page Submit (3)
Click Count (4)

End of Block: 2M:3F 50th Female - Manipulation Reminder

Start of Block: 2M:3F 30th Male - Manipulation Reminder

2M:3F 30th Male Rem

Q2600 Think about how much you earn in Stamola compared to others again. Remember how your income compares to women and men. Spend 15 seconds remembering what life in Stamola would be like if you earnt this much in Stamolean society. How might it shape your romantic relationships with ${e://Field/opgenpeople}? How might it influence what you want in a partner? As you progress throughout the survey, answer any questions with these thoughts in mind. After 15 seconds, a button will appear in the lower right hand corner of the screen and you may proceed to the next part of the survey.

Q2601 Timing
First Click (1)
Last Click (2)
Page Submit (3)
Click Count (4)

End of Block: 2M:3F 30th Male - Manipulation Reminder

Start of Block: 2M:3F 30th Female - Manipulation Reminder

2M:3F 30th F Rem

Q2660 Think about how much you earn in Stamola compared to others again. Remember how your income compares to women and men. Spend 15 seconds remembering what life in Stamola would be like if you earnt this much in Stamolean society. How might it shape your romantic relationships with ${e://Field/opgenpeople}? How might it influence what you want in a partner? As you progress throughout the survey, answer any questions with these thoughts in mind. After 15 seconds, a button will appear in the lower right hand corner of the screen and you may proceed to the next part of the survey.

Q2661 Timing
First Click (1)
Last Click (2)
Page Submit (3)
Click Count (4)

End of Block: 2M:3F 30th Female - Manipulation Reminder

Start of Block: 2M:3F 10th Male - Manipulation Reminder

2M:3F 10th Male Rem

Q2603 Think about how much you earn in Stamola compared to others again. Remember how your income compares to women and men. Spend 15 seconds remembering what life in Stamola would be like if you earnt this much in Stamolean society. How might it shape your romantic relationships with ${e://Field/opgenpeople}? How might it influence what you want in a partner? As you progress throughout the survey, answer any questions with these thoughts in

mind.   After 15 seconds, a button will appear in the lower right hand corner of the screen and you may proceed to the next part of the survey.

Q2604 Timing
First Click (1)
Last Click (2)
Page Submit (3)
Click Count (4)

End of Block: 2M:3F 10th Male - Manipulation Reminder

Start of Block: 2M:3F 10th Female - Manipulation Reminder

2M:3F 10th F Rem

Q2663 Think about how much you earn in Stamola compared to others again. Remember how your income compares to women and men.   Spend 15 seconds remembering what life in Stamola would be like if you earnt this much in Stamolean society.   How might it shape your romantic relationships with ${e://Field/opgenpeople}? How might it influence what you want in a partner?   As you progress throughout the survey, answer any questions with these thoughts in mind.   After 15 seconds, a button will appear in the lower right hand corner of the screen and you may proceed to the next part of the survey.

Q2664 Timing
First Click (1)
Last Click (2)
Page Submit (3)
Click Count (4)

End of Block: 2M:3F 10th Female - Manipulation Reminder

Start of Block: 1M:2F 90th Male - Manipulation Reminder

1M:2F 90th Male Rem

Q2606 Think about how much you earn in Stamola compared to others again. Remember how your income compares to women and men.   Spend 15 seconds remembering what life in Stamola would be like if you earnt this much in Stamolean society.   How might it shape your romantic relationships with ${e://Field/opgenpeople}? How might it influence what you want in a partner?   As you progress throughout the survey, answer any questions with these thoughts in mind.   After 15 seconds, a button will appear in the lower right hand corner of the screen and you may proceed to the next part of the survey.

Q2607 Timing
First Click (1)
Last Click (2)
Page Submit (3)
Click Count (4)

End of Block: 1M:2F 90th Male - Manipulation Reminder

Start of Block: 1M:2F 90th Female - Manipulation Reminder

1M:2F 90th F Rem

Q2666 Think about how much you earn in Stamola compared to others again. Remember how your income compares to women and men.   Spend 15 seconds remembering what life in Stamola would be like if you earnt this much in Stamolean society.   How might it shape your romantic relationships with ${e://Field/opgenpeople}? How might it influence what you want in a partner?   As you progress throughout the survey, answer any questions with these thoughts in mind.   After 15 seconds, a button will appear in the lower right hand corner of the screen and you may proceed to the next part of the survey.

Q2667 Timing
First Click (1)
Last Click (2)
Page Submit (3)
Click Count (4)

End of Block: 1M:2F 90th Female - Manipulation Reminder

Start of Block: 1M:2F 70th Male - Manipulation Reminder

1M:2F 70th Male Rem

Q2609 Think about how much you earn in Stamola compared to others again. Remember how your income compares to women and men.   Spend 15 seconds remembering what life in Stamola would be like if you earnt this much in Stamolean society.   How might it shape your romantic relationships with ${e://Field/opgenpeople}? How might it influence what you want in a partner?   As you progress throughout the survey, answer any questions with these thoughts in mind.   After 15 seconds, a button will appear in the lower right hand corner of the screen and you may proceed to the next part of the survey.

Q2610 Timing
First Click (1)
Last Click (2)
Page Submit (3)
Click Count (4)

End of Block: 1M:2F 70th Male - Manipulation Reminder

Start of Block: 1M:2F 70th Female - Manipulation Reminder

1M:2F 70th F Rem

Q2669 Think about how much you earn in Stamola compared to others again. Remember how your income compares to women and men.   Spend 15 seconds remembering what life in Stamola would be like if you earnt this much in Stamolean society.   How might it shape your romantic relationships with ${e://Field/opgenpeople}? How might it influence what you want in a partner?   As you progress throughout the survey, answer any questions with these thoughts in mind.   After 15 seconds, a button will appear in the lower right hand corner of the screen and you may proceed to the next part of the survey.

Q2670 Timing
First Click (1)
Last Click (2)
Page Submit (3)
Click Count (4)

End of Block: 1M:2F 70th Female - Manipulation Reminder

Start of Block: 1M:2F 50th Male - Manipulation Reminder

1M:2F 50th Male Rem

Q2612 Think about how much you earn in Stamola compared to others again. Remember how your income compares to women and men. Spend 15 seconds remembering what life in Stamola would be like if you earnt this much in Stamolean society. How might it shape your romantic relationships with ${e://Field/opgenpeople}? How might it influence what you want in a partner? As you progress throughout the survey, answer any questions with these thoughts in mind. After 15 seconds, a button will appear in the lower right hand corner of the screen and you may proceed to the next part of the survey.

Q2613 Timing
First Click (1)
Last Click (2)
Page Submit (3)
Click Count (4)

End of Block: 1M:2F 50th Male - Manipulation Reminder

Start of Block: 1M:2F 50th Female - Manipulation Reminder

1M:2F 50th F Rem

Q2672 Think about how much you earn in Stamola compared to others again. Remember how your income compares to women and men. Spend 15 seconds remembering what life in Stamola would be like if you earnt this much in Stamolean society. How might it shape your romantic relationships with ${e://Field/opgenpeople}? How might it influence what you want in a partner? As you progress throughout the survey, answer any questions with these thoughts in

mind.   After 15 seconds, a button will appear in the lower right hand corner of the screen and you may proceed to the next part of the survey.

Q2673 Timing
First Click (1)
Last Click (2)
Page Submit (3)
Click Count (4)

End of Block: 1M:2F 50th Female - Manipulation Reminder

Start of Block: 1M:2F 30th Male - Manipulation Reminder

1M:2F 30th Male Rem

Q2615 Think about how much you earn in Stamola compared to others again. Remember how your income compares to women and men.   Spend 15 seconds remembering what life in Stamola would be like if you earnt this much in Stamolean society.   How might it shape your romantic relationships with ${e://Field/opgenpeople}? How might it influence what you want in a partner?   As you progress throughout the survey, answer any questions with these thoughts in mind.   After 15 seconds, a button will appear in the lower right hand corner of the screen and you may proceed to the next part of the survey.

Q2616 Timing
First Click (1)
Last Click (2)
Page Submit (3)
Click Count (4)

End of Block: 1M:2F 30th Male - Manipulation Reminder

Start of Block: 1M:2F 30th Female - Manipulation Reminder

1M:2F 30th F Rem

Q2675 Think about how much you earn in Stamola compared to others again. Remember how your income compares to women and men.   Spend 15 seconds remembering what life in Stamola would be like if you earnt this much in Stamolean society.   How might it shape your romantic relationships with ${e://Field/opgenpeople}? How might it influence what you want in a partner?   As you progress throughout the survey, answer any questions with these thoughts in mind.   After 15 seconds, a button will appear in the lower right hand corner of the screen and you may proceed to the next part of the survey.

Q2676 Timing
First Click (1)
Last Click (2)
Page Submit (3)
Click Count (4)

End of Block: 1M:2F 30th Female - Manipulation Reminder

Start of Block: 1M:2F 10th Male - Manipulation Reminder

1M:2F 10th Male Rem

Q2618 Think about how much you earn in Stamola compared to others again. Remember how your income compares to women and men.   Spend 15 seconds remembering what life in Stamola would be like if you earnt this much in Stamolean society.   How might it shape your romantic relationships with ${e://Field/opgenpeople}? How might it influence what you want in a partner?   As you progress throughout the survey, answer any questions with these thoughts in mind.   After 15 seconds, a button will appear in the lower right hand corner of the screen and you may proceed to the next part of the survey.

Q2619 Timing
First Click (1)
Last Click (2)
Page Submit (3)
Click Count (4)

End of Block: 1M:2F 10th Male - Manipulation Reminder

Start of Block: 1M:2F 10th Female - Manipulation Reminder

1M:2F 10th F Rem

Q2678 Think about how much you earn in Stamola compared to others again. Remember how your income compares to women and men. Spend 15 seconds remembering what life in Stamola would be like if you earnt this much in Stamolean society. How might it shape your romantic relationships with ${e://Field/opgenpeople}? How might it influence what you want in a partner? As you progress throughout the survey, answer any questions with these thoughts in mind. After 15 seconds, a button will appear in the lower right hand corner of the screen and you may proceed to the next part of the survey.

Q2679 Timing
First Click (1)
Last Click (2)
Page Submit (3)
Click Count (4)

End of Block: 1M:2F 10th Female - Manipulation Reminder

Start of Block: Mate Preferences 2

EastwickRanking Below are listed a set of characteristics. **Please rank them on their desirability when choosing a long-term partner** (e.g., ${e://Field/ltr}) **in Stamola.** Click and drag to move items to their appropriate ranking, giving a "1" to the most desirable characteristic in a long-term partner; a "2" to the second most desirable characteristic in a long-term partner; a

"3" to the third most desirable characteristic, and so on, down to "14" for the 14th most desirable characteristic in a long-term partner. *This task may take a moment to complete. Take your time.*

_______ Financially secure (1)
_______ Ambitious (2)
_______ Good job (3)
_______ Smells good (4)
_______ Attractive (5)
_______ Confident (6)
_______ Open to new experiences, complex (7)
_______ Sexy (8)
_______ Dresses well (9)
_______ Sensitive (10)
_______ Nice body (11)
_______ Successful (12)
_______ Adventurous (13)
_______ Extraverted, enthusiastic (14)

Page Break

BussRating **Rate each of the following characteristics on how important or desirable it would be for you when choosing a long-term partner** (e.g., ${e://Field/ltr}) **in Stamola.** (0 = Irrelevant or unimportant, 3 = Indispensable)

	Irrelevant or unimportant 0 (1)	1 (2)	2 (3)	Indispensable 3 (4)
Good financial prospect (Good financial prospect)	○	○	○	○
Ambition-industriousness (Ambition-industriousness)	○	○	○	○
Dependable character (Dependable character)	○	○	○	○
Emotional stability (Emotional stability)	○	○	○	○
Pleasing disposition (Pleasing disposition)	○	○	○	○
Mutual attraction (Mutual attraction)	○	○	○	○
Good health (Good health)	○	○	○	○
Desire for home-children (Desire for home-children)	○	○	○	○
Refinement (Refinement)	○	○	○	○
Good cook-housekeeper (Good cook-housekeeper)	○	○	○	○

Chastity (Chastity)	○	○	○	○
Education-intelligence (Education-intelligence)	○	○	○	○
Sociability (Sociability)	○	○	○	○
Similar religious background (Similar religious background)	○	○	○	○
Good looks (Good looks)	○	○	○	○
Similar educational background (Similar educational background)	○	○	○	○
Favorable social status (Favorable social status)	○	○	○	○
Similar political background (Similar political background)	○	○	○	○

End of Block: Mate Preferences 2

Start of Block: Mate-seeking Questionnaire

X→

Mate-seeking **Think about your life in Stamola, and indicate the degree to which you agree or disagree with the following.** In Stamola…

	-3 (-3)	-2 (-2)	-1 (-1)	0 (0)	1 (1)	2 (2)	3 (3)
I would be interested in finding a long-term romantic partner. (Mate-seeking_1)	○	○	○	○	○	○	○
I would not be interested in meeting a long-term romantic partner. (Mate-seeking_2)	○	○	○	○	○	○	○
Starting a serious romantic relationship would not be a high priority for me. (Mate-seeking_3)	○	○	○	○	○	○	○
I would rarely think about finding a serious romantic partner. (Mate-seeking_4)	○	○	○	○	○	○	○

End of Block: Mate-seeking Questionnaire

Start of Block: Debrief

debrief Thank you for your participation. REDACTED FOR PEER REVIEW

End of Block: Debrief
